# A speedy route to sterically encumbered, benzene-fused derivatives of privileged, naturally occurring hexahydropyrrolo[1,2-*b*]isoquinoline

**DOI:** 10.3762/bjoc.13.138

**Published:** 2017-07-18

**Authors:** Olga Bakulina, Alexander Ivanov, Vitalii Suslonov, Dmitry Dar’in, Mikhail Krasavin

**Affiliations:** 1 Institute of Chemistry, Saint Petersburg State University, 26 Universitetsky prospekt, Peterhof 198504, Russia

**Keywords:** Castagnoli–Cushman reaction, diastereoselectivity, homophthalic anhydride, indolenines, lactam synthesis, multicomponent reactions

## Abstract

A series of 15 benzene-fused hexahydropyrrolo[1,2-*b*]isoquinolonic acids with substantial degree of steric encumbrance has been prepared via a novel variant of the Castagnoli–Cushman reaction of homophthalic anhydride (HPA) and various indolenines. The employment of a special kind of a cyclic imine component reaction allowed, for the first time, isolating a Mannich-type adduct between HPA and an imine component which has been postulated but never obtained in similar reactions.

## Introduction

**Figure 1:**
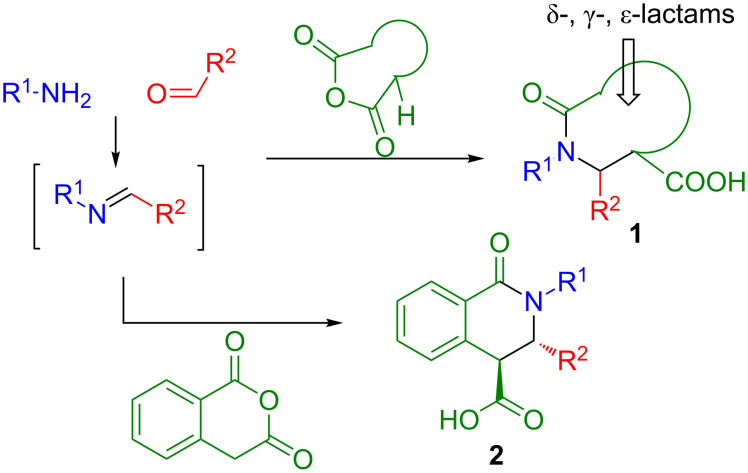
The Castagnoli–Cushman reaction (CCR).

The reaction of imines (prepared in a separate step or generated in situ) with α-C–H dicarboxylic acid anhydrides (known as the Castagnoli–Cushman reaction or CCR [1]) offers a direct entry into lactam frameworks **1** of various sizes (traditionally, δ- and γ- [2–3] and, more recently, ε-lactams [4–5]) containing a carboxylic acid functionality. Employment of homophthalic anhydride (HPA) in this reaction delivers medicinally important, most often *trans*-configured [6–7] tetrahydroisoquinolonic acids **2** (Figure 1) which have found utility as probe compounds or therapeutic agents in diverse areas such as neuroprotection [8], diabetes [9], and cancer [10].

**Figure 2:**
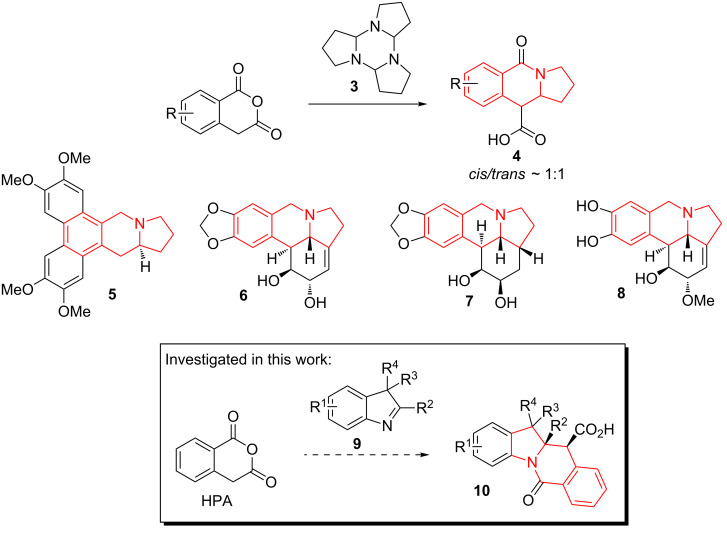
Assembly of hexahydropyrrolo[1,2-*b*]isoquinoline core via the CCR and its occurrence in natural and synthetic bioactive compounds.

The use of cyclic imines (or surrogates thereof such as isoquinoline [11]) in the CCR is quite scarce in the literature [12–14]. As an example, 1-pyrroline (in the form of its trimer **3**) has been reported by Smith and co-workers [15–17] to condense efficiently with HPA analogs to deliver hexahydropyrrolo[1,2-*b*]isoquinolones **4**. The hexahydropyrrolo[1,2-*b*]isoquinoline core in general is ubiquitous to many natural products exemplified by tylophorine (**5**) [18], lycorine (**6**) [19] and its entire family of alkaloids, including zephyranthine (**7**) [20] and galantine (**8**) [21]. Considering the plethora of biological activities displayed by the lycorine and tylophorine alkaloids (such as pro-apoptotic [22], antiviral [23], hypoxia-inducible factor-1 inhibitory [24]), the scaffold can be confidently regarded as privileged [25]. Recently, we [26] and others [27] reported the use of indolenines as non-classical inputs for the Joullié–Ugi reaction and for subsequent preparation [28] of sterically encumbered, constrained peptidomimetic frameworks. Diversely substituted indolenines **9** are easy to prepare via the Fischer indole synthesis [26] and their use in the CCR can be expected to result in hexahydropyrrolo[1,2-*b*]isoquinolone derivatives fused with benzene **10** that have pronounced three-dimensional features and potentially contain several quaternary carbon centers (Figure 2). The first aspect has been recently recognized [29] as a central principle in drug design ensuring effective interaction of small molecules with protein targets and lower off-target effects. The presence of quaternary carbons is characteristic of the natural products domain and is also gaining prominence in medicinal chemistry [30]. Herein, we disclose the results obtained and observations made in the course of our attempt to involve **9** in reactions with HPA. Notably, due to its non-planar tetracyclic character, the hexahydropyrrolo[1,2-*b*]isoquinolone fused with benzene scaffold (present in **10**) clearly appears related to (though topologically distinct from) the natural and synthetic camptothecin-like topoisomerase inhibitors [31].

## Results and Discussion

**Figure 3:**
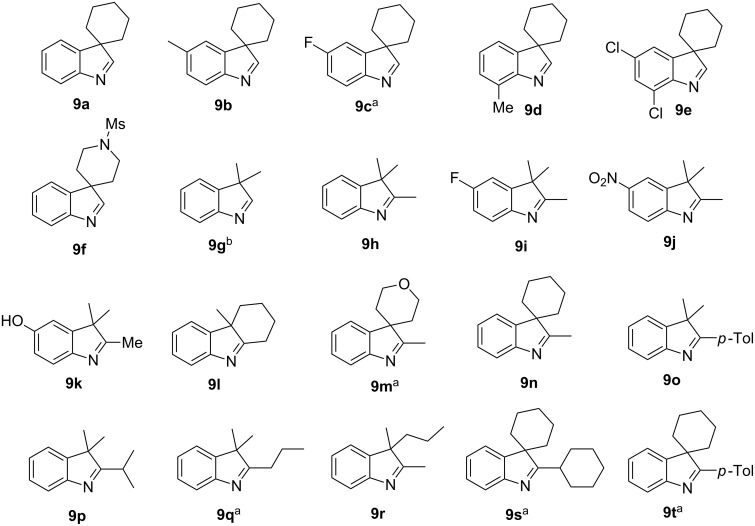
Indolenine substrates **9a–t** investigated in this work. ^a^Prepared for the first time (the rest are known compounds, see Experimental section). ^b^Unstable to isolation; was taken in the CCR step as a solution in chloroform.

The study commenced with the synthesis of set of 2-H as well as 2-substituted indolenines **9a–t** using a previously published procedure (Figure 3) [26]. Acetonitrile was previously found [32] to be an effective solvent promoting the CCR of HPA with acyclic amines at room temperature. As it also facilitated the isolation of the tetrahydroisoquinolonic acid product, it was chosen to study the CCR of **9a–t**. The latter tend to precipitate in a pure form (often as a single diastereomer) from the reaction mixture and can be conveniently isolated by filtration. This prior observation also held true for the reaction between HPA and most of indolenines **9**.

As shown in Table 1, all of the 2-unsubstituted indolenines **9a–g** and many 2-substituted one **9h–o** furnished the expected respective tetracyclic tetrahydroisoquinolonic acids **10** on treatment with HPA (1.0 equiv) in acetonitrile (2 mL/mmol). Notably, when the CCR with HPA was repeated for **9h** in other solvents (toluene, chloroform or DMF) at room temperature, this resulted in a similar product yield and diastereomeric ratio. However, the isolation of **10h** from the respective reaction mixtures was distinctly cumbersome, which only confirmed acetonitrile to be the solvent of choice for these reactions.

**Table 1:** Indolo[1,2-*b*]isoquinolonic acids **10** obtained via the CCR of indolenines **9**.

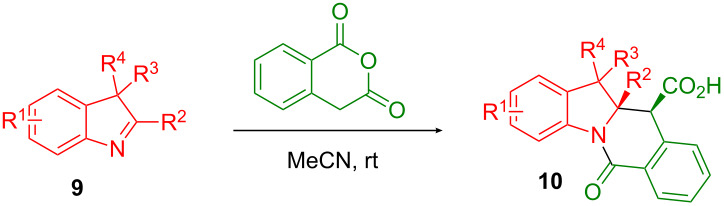					

Entry	**9**	Product **10**	Time	Isolated yield, %^a^	*anti*/*syn*

1	**9a**	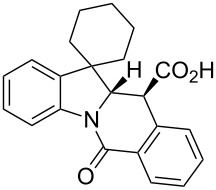 **10a**	2 h	56	>20:1^b^
2	**9b**	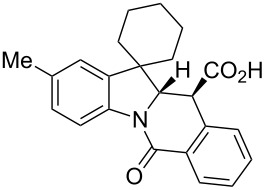 **10b**	2 h	73	>20:1^b^
3	**9c**	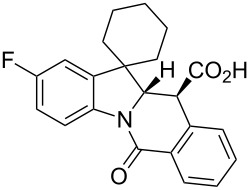 **10c**	24 h	66	3.3:1^c^
4	**9d**	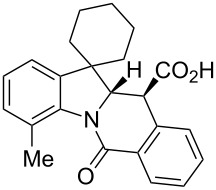 **10d**	3 h	84	>20:1
5	**9e**	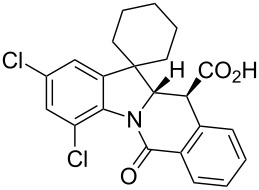 **10e**	48 h	66	0.8:1^d^
6	**9f**	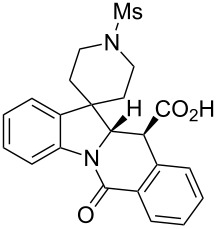 **10f**	72 h	79	2:1^d^
7	**9g**	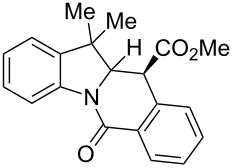 **10g‘**	16 h	87	1.4:1^d^
8	**9h**	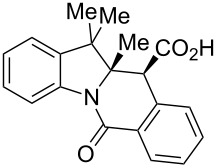 **10h**	16 h	86	4.3:1^c^
9	**9i**	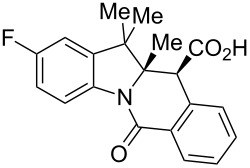 **10i**	48 h	72	3:1^d^
10	**9j**	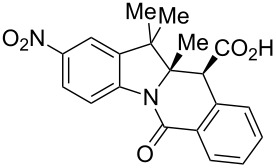 **10j**	48 h	73	>20:1
11	**9k**	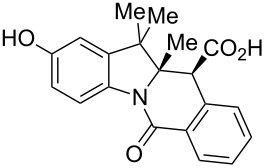 **10k**	48 h	57	6.5:1^c^
12	**9l**	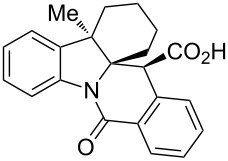 **10l**	48 h	81	>20:1
13	**9m**	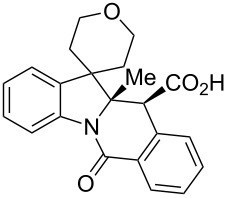 **10m**	72 h	79	6:1^c^
14	**9n**	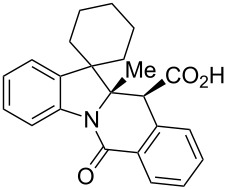 **10n**	25 d	93	>20:1
15	**9o**	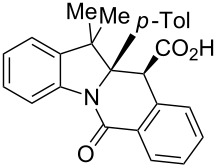 **10o**	50 d	52	11:1
16	**9p**	–	7 d	0	–
17	**9q**	–	7 d	0	–
18	**9r**	–	7 d	0	–
19	**9s**	–	7 d	0	–
20	**9t**	–	7 d	0	–

^a^Isolated yield of the product precipitate from the reaction mixture. ^b^Additional quantity of the *anti-* and/or *syn*-diastereomer(s) isolated from the filtrate as respective methyl esters **10'** (see Experimental section). ^c^Pure *anti*-diastereomer obtained by crystallization. ^d^Isolated and characterized as respective methyl esters **10'**.

**Figure 4:**
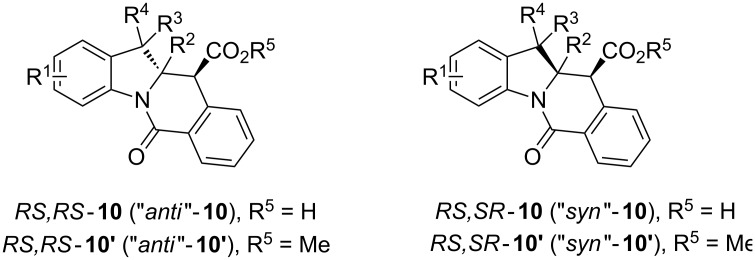
*Anti*- and *syn*-diastereomers of **10** and **10'**.

A number of observations emerged from examination of the results in Table 1. In all cases (except entry 7 where carboxylic acid **10g** did not precipitate from the reaction mixture and was isolated as respective methyl ester **10g'**), the major diastereomer was shown to possess the (*RS,RS*)-configuration (vide infra) and is referred to as ‘*anti*‘ throughout this article (considering orientation of carboxylic group relative to C^11a^–C^12^ bond of the five-membered ring); the minor, (*RS,SR*)-configured diastereomer is referred to as ‘*syn*’ (Figure 4). A good to excellent yield of pure *anti*-diastereomer was obtained with **9a**,**b**, **9d**, **9j**, **9l**, **9n**,**o** (Table 1, entries 1, 2, 4, 10, 12, 14 and 15) by simple filtration. We have also shown that in some of these cases (Table 1, entries 1 and 2) an additional quantity of *anti*- and/or *syn*-configured CCR product could be recovered from the filtrate in the form of respective methyl esters after *O*-methylation and chromatographic separation (see Experimental section): *syn*-**10a'** (7%), *anti*-**10a'** (12%), *syn*-**10b'** (13%). In those cases when the carboxylic acid precipitate contained a significant proportion of the *syn*-configured CCR product (Table 1, entries 3, 8, 11 and 13, *anti*/*syn* ratio ranging from 3.3:1 to 6.5:1), the latter was removed by crystallization and the respective pure *anti*-diastereomers (*anti*-**10c**, **10h**, **10k** and **10m**) were obtained and characterized. In certain instances (Table 1, entries 5–7, and 9), isolation of pure diastereomeric CCR adducts was achieved by total esterification of the carboxylic acid product mixture and chromatographic separation of the respective methyl esters (the 0.8:1 *anti/syn*-**10e** mixture stereoconverged to *anti*-**10e'** on esterification, vide infra).

**Figure 5:**
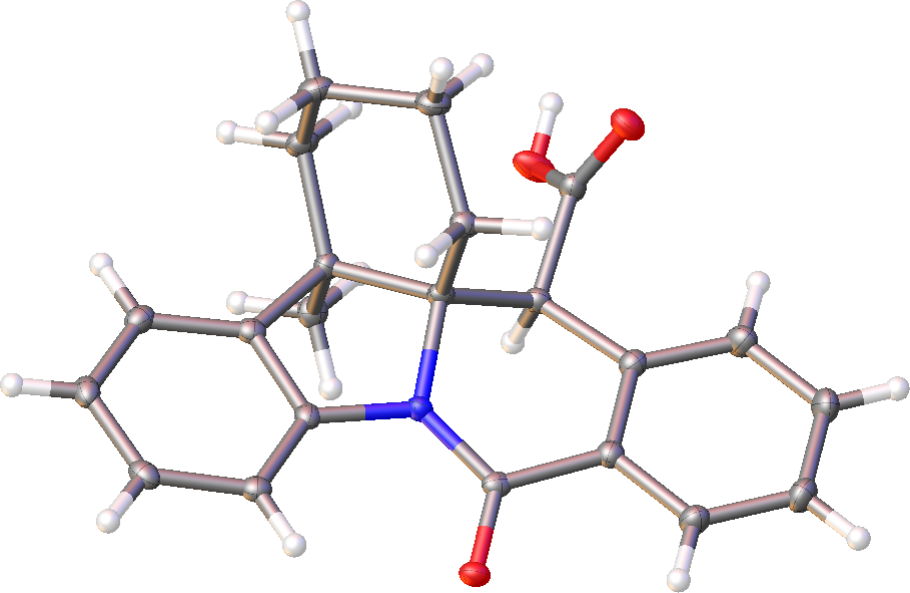
Single-crystal X-ray structure of compound **10l**.

The *trans*-diastereoselectivity of the CCR is well documented in the literature [33] and is, therefore, unsurprising. However, the stereocontrol achieved in this reaction over 3 stereocenters present in **10l** (obtained in 81% yield as a single diastereomer) is certainly quite noteworthy and was confirmed by X-ray analysis (Figure 5).

**Scheme 1:**
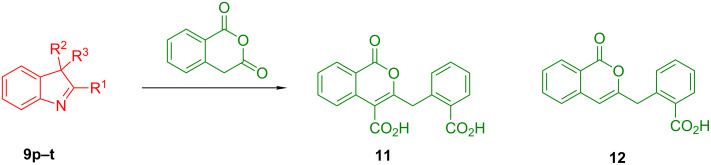
Formation of unwanted products **11** and **12** in lieu of the CCR with **9p–t**.

The tolerance of the reaction to the substitution pattern in the aromatic portion of the indolenines appears rather broad, both in terms of the electronic character of substituents and steric effects – although substituents in position 7 of the indolenine significantly affect the conformational behavior of the respective CCR adducts **10d**,**e** (vide infra). Notably, free phenolic hydroxy function is well tolerated (**9k** → **10k**) which is in line with literature reports [33]. However, the steric situation around the five-membered ring of indolenines had a profound effect on the reaction times and even the ability of certain indolenines to act as competent partners in the CCR. 2-Substituted indolenines **9h–o** required significantly longer times (from 16 h to 50 days) to be converted to the respective CCR products, compared to their 2-unsubstituted counterparts. Indolenines **9p–t** failed to undergo the reaction with HPA either at rt or at reflux temperature in acetonitrile or toluene. Attempts to trigger the reaction with these substrates by excluding solvent altogether [34] or applying microwave irradiation (up to 2 h at 200 °C in MeCN) were also unsuccessful. Any appreciable conversion led to predominant formation of HPA dimer **11** and product of its decarboxylation **12** (Scheme 1). The formation of these two products (observed by ^1^H NMR) was recently reported by Knapp et al. [35] as a result from the treatment of HPA with a strong base (which was absent in our case). The failure to activate sterically hindered indolenines toward the CCR using forcing conditions justifies conducting the reaction at room temperature, which led to clean conversions and good product yields despite the excruciatingly long reaction times (Table 1, entries 14 and 15).

**Figure 6:**
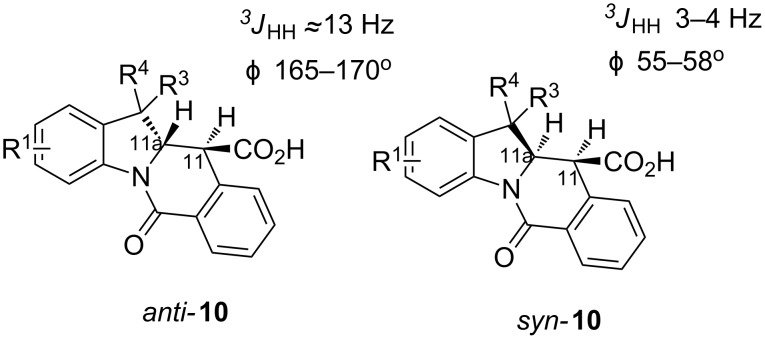
Typical *J*(H^11^-H^11a^)-values and corresponding dihedral angles for *syn-* and *anti*-diastereomers of compounds **10** derived from 2*H*-indolenines.

In order to ensure a correct stereochemical assignment of all major (*anti*) and minor (*syn*) products obtained in the reactions discussed herein, we obtained single-crystal X-ray structures of sixteen CCR adducts synthesized in this work and correlated this structural information with the NMR behavior of these compounds. The range of the coupling constant *J*(H^11^-H^11a^) values appears a straightforward criterion for relative stereochemistry assignment in compounds **10** derived from 2*H*-indolenines. As summarized in Figure 6 (see also Table S1 in Supporting Information File 1), *anti*-diastereomers display this coupling constant around 13 Hz, while it is in the 3-4 Hz range for *syn*-isomers.

**Figure 7:**
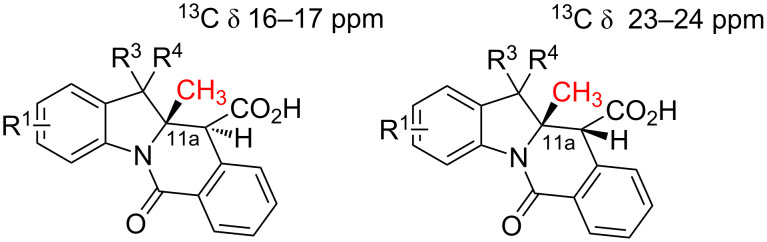
The difference in the ^13^C NMR chemical shifts of the angular methyl group between *syn*- and *anti*-diastereomers of 11a-Me in compounds **10h–k**, **10m,n**.

**Figure 8:**
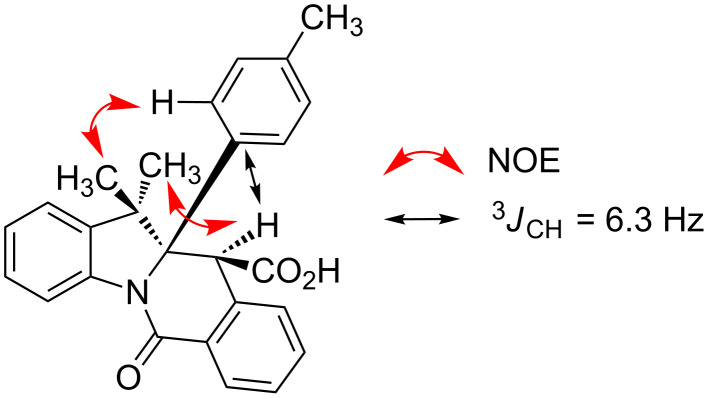
Criteria for stereochemistry assignment of *anti*-**10o**.

According to the X-ray analysis, the dihedral angle values in the C^11^H–C^11a^H fragment lie within the 165–170° and 55–58° range for *anti*- and *syn*-diastereomers, respectively (Supporting Information File 1, Table S1). Thus, for most of the 2*H*-indolenine-derived compounds (except for **10d**,**e**), there appears to be a good correlation between the relative stereochemistry of **10**, *J*(H^11^-H^11a^) values observed in the ^1^H NMR spectra of their solutions and said dihedral angle in crystals. Surprisingly, compounds **10d**,**e** (derived from more sterically congested 7-substituted indolenines **9d**,**e**) display the *J*(H^11^-H^11a^) values of 3.0 Hz for both diastereomers, which is inconsistent with the regular values of corresponding dihedral angles (166.3° and 169.7° for *anti*-**10d** and *anti*-**10e**, respectively; 57.5° for *syn*-**10e**) measurable in the X-ray structures of these compounds. We reasoned that such a phenomenon could be rationalized by a different conformer population in the solution compared to solid state. This hypothesis was preliminary confirmed by the results of variable-temperature NMR experiments summarized in Supporting Information File 1 (Figures S1–S6). Another potentially useful criterion for stereochemistry assignment of compounds **10** derived from 2-methylindolenines **10h–k**, **10m**,**n** is the range of ^13^C chemical shifts observed for the angular methyl group in their *syn*- and *anti*-diastereomers (Supporting Information File 1, Figure 7, Table S2). These findings were also verified by the available X-ray information on these compounds. Finally, the relative stereochemistry of compound **10o** (for which neither X-ray structures nor reliable NMR criteria were available) was assigned on the basis of the through-space interactions observable in its NOESY spectrum and the value of a *^3^J*_CH_ coupling constant (Supporting Information File 1, Figure 8, Figures S7–9).

**Scheme 2:**
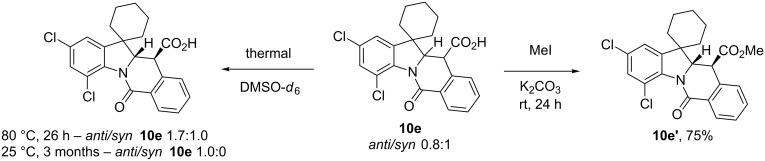
*Syn*/*anti* isomerization of compound **10e**.

Besides the anomalous NMR behavior discussed above, compound **10e** displayed a number of unusual tendencies which may shed some light on the diastereomeric integrity of CCR adducts in general as well as on the mechanism of their formation. Compound **10e** initially formed as a 0.8:1 mixture of *anti*/*syn* diastereomers (Table 1). However, it was promptly noted that this mixture converges to thermodynamically more stable *anti*-**10e** (the isomerization occurs on heating to 80 °C or, more slowly, even at room temperature). Esterification of *anti*/*syn*-**10e** in the presence of potassium carbonate led to the formation of *anti*-**10e'** as a sole product, presumably, via a base-promoted enolization and subsequent isomerization of the *syn*-**10e** (**10e',** Scheme 2).

**Scheme 3:**
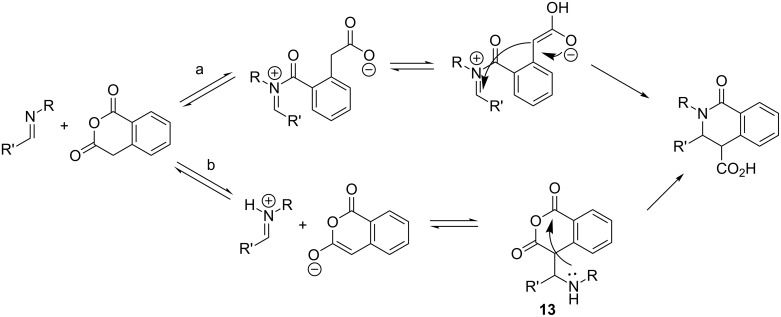
Alternative mechanistic pathways for the CCR.

Enolization is thought to be a key event in the formation of the CCR products which can occur via two alternative mechanisms: (a) *N*-acylation of the imine component followed by intramolecular Mannich reaction or (b) Mannich-type addition of the HPA enolate to a protonated imine component followed by intramolecular aminolysis of the cyclic anhydride moiety in Mannich adduct **13** (Scheme 3) [1].

Investigation of the CCR leading to the formation of **10e** undertaken in this work led to a serendipitous important observation that exposure of **9e** to an equimolar amount of HPA in acetonitrile at low temperature (5 °C) and higher dilution (two-fold compared to that used throughout this study) over 2 days led to a predominant formation of the respective Mannich adduct **13e** which crystallized out as a single diastereomer from the reaction mixture along with *syn/anti*-**10e** mixture and was separated from the latter mechanically under a microscope. Adduct **13e** (which has been postulated in the literature as a principal intermediate in the CCR [1,28] but never isolated) was characterized by means of ^1^H and ^13^C NMR spectroscopy as well as X-ray crystallography. The isolation, for the first time, of the Mannich-type adduct **13** between HPA and an imine clearly attests to the viability of mechanistic pathway (b) shown in Scheme 3.

**Scheme 4:**
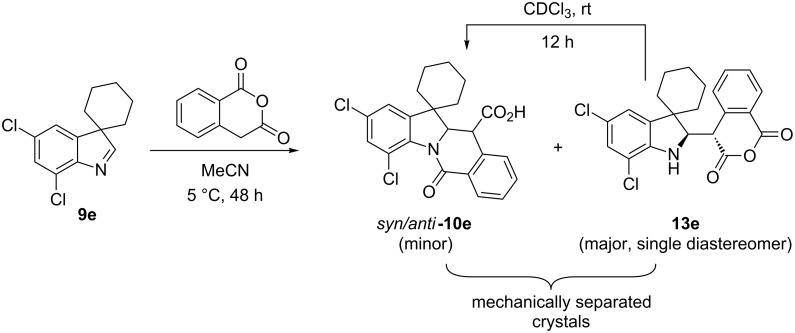
Formation and fate of Mannich adduct **13e**.

When left at room temperature for 12 h as a solution in CDCl_3_, single diastereomer **13e** fully converted itself into a ca. 1:1 *anti/syn*-**10e** (Scheme 4).

**Scheme 5:**
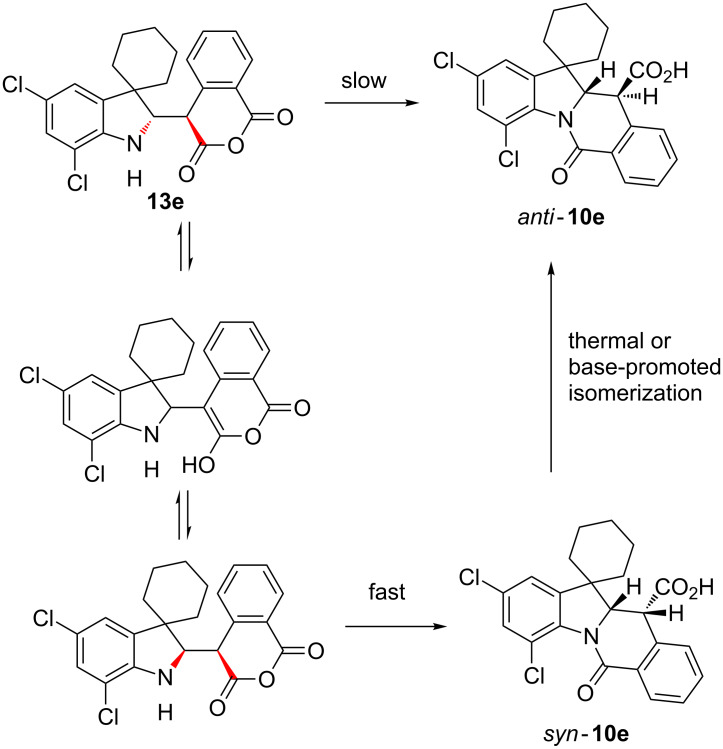
Mechanistic rationale for the **13e**→ *syn/anti*-**10e** conversion.

Conversion of diastereomerically pure **13e** into a mixture of diastereomers can be rationalized by a faster formation of kinetic product *syn*-**10e** preceded by enolization, in competition with direct albeit slow conversion **13e** → *anti*-**10e** (Scheme 5).

**Scheme 6:**
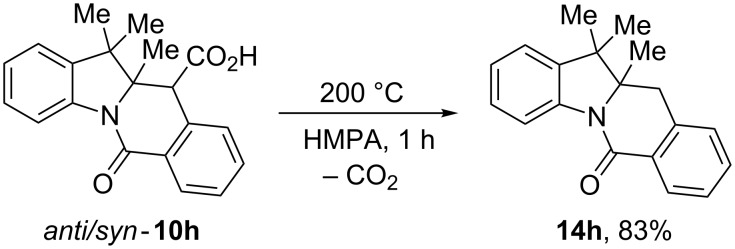
Decarboxylation of *anti*/*syn*-**10h**.

We mentioned that tetracyclic compounds **10** carry resemblance to natural as well as synthetic camptothecin-like topoisomerase inhibitors (vide supra). Compounds **10** are endowed with HPA-derived carboxylic acid functionality which may facilitate or prevent compounds’ binding to DNA or DNA-topoisomerase complex. Depending on the medicinal chemistry context, compounds **10** can be decarboxylated to deliver sterically encumbered tetracyclic lactams **14** lacking the carboxylic acid group as we showed for exemplary compound **10h**. The 4.3:1 *anti/syn* mixture of diastereomers of **10h** rapidly and cleanly lost the carboxylic function at 200 °C and gave 83% yield of racemic compound **14h** (Scheme 6).

## Conclusion

We have described a novel variant of the Castagnoli–Cushman reaction employing indolenines as cyclic imine components in the reaction with homophthalic anhydride (HPA). The compounds obtained contain a benzene-fused, privileged, naturally occurring hexahydropyrrolo[1,2-*b*]isoquinoline core and are distinctly encumbered from steric perspective. The novel compounds have been characterized by NMR spectroscopy, high-resolution mass spectrometry and X-ray crystallography and certain regularities in the NMR behavior have been established, leading a set of rules for stereochemical assignment based on the NMR data. A Mannich-type adduct between HPA and an imine (previously only postulated as a crucial intermediate en route to CCR products) has been isolated for the first time and fully characterized. Certain insights into the role of enolization equilibria in the formation and diastereomeric integrity of the CCR adducts has been obtained. The synthetic methodology described herein significantly expands the scope and tests the limits of the sterically permitted Castagnoli–Cushman reaction.

## Experimental

**General information.** NMR spectroscopic data were recorded with a 400 MHz (400.13 MHz for ^1^H and 100.61 MHz for ^13^C) and a 500 MHz (500.03 MHz for ^1^H and 125.7 MHz for ^13^C) spectrometers in DMSO-*d*_6_ or in CDCl_3_ and were referenced to residual solvent proton signals (δ_H_* = 7.26 and 2.50 ppm, respectively) and solvent carbon signals (*δ*_C_* = 77.2 and 39.5 ppm, respectively). Coupling constants, *J* are reported in Hz. Melting points were determined with an automated heat block instrument and are uncorrected. Mass spectra were recorded with a HRMS-ESI-qTOF spectrometer (electrospray ionization mode). X-ray single crystal analyses were performed with monochromated Mo Kα or Cu Kα radiation, respectively. Column chromatography was performed on silica gel 60 (230–400 mesh). For TLC analysis UV_254_ silica gel coated plates were used. MeCN was distilled from P_2_O_5_ and stored over molecular sieves 4 Å. Homophthalic anhydride was acquired from a commercial source, stored at 5 °C and used as received. All indolenines **9** were stored in sealed vials at 5 °C in the dark.

CCDC 1503093 (**13e**), 1503094 (*anti-***10c**), 1503095 (*anti-***10d**), 1503096 (*anti-***10g'**), 1503097 (*anti-***10e**), 1503098 (*anti****-*10a'**), 1503099 (*anti-***10f**), 1503100 (*syn-***10a'**), 1503101 (*syn-***10f**), 1503102 (*anti-***10e'**), 1503103 *(anti-***10n**), 1503104 (*anti-***10i**), 1503105 (*syn-***10e**), 1503106 (*anti-***10l**), 1470399 (*anti-***10b**), 1470389 (*anti-***10h**), 1461790 (*anti-***10j**) contain the supplementary crystallographic data for this paper. These data can be obtained free of charge from The Cambridge Crystallographic Data Centre via http://www.ccdc.cam.ac.uk.

**Indolenines 9**. Indolenines **9a**,**b** [26], **9d–g** [26], **9h** [36], **9i** [37], **9j** [38], **9k** [39], **9p** [40] are known compounds and were prepared from the arylhydrazine hydrochlorides and respective aldehydes or ketones according to the literature protocols.

**General procedure 1. Synthesis of indolenines 9c,l,n,o,q–t.** To a screw-cap vial containing suspension of corresponding arylhydrazine hydrochloride in glacial AcOH (15 mL) the carbonyl compound (1.1 equiv) was added in one portion. The reaction mixture was stirred at 55–60 °C for 4 h (or at reflux for 2–16 h) and concentrated in vacuo at 40 °C. The residue was diluted with DCM (50 mL) and filtered through Celite. The resulting solution was passed through a short pad of silica, washed with sat. NaHCO_3_ and water. The organic layer was dried over Na_2_SO_4_ and concentrated under reduced pressure to give pure indolenines **9c**,**l**,**s**. Indolenines **9n**,**o**,**q**,**r**,**t** were subjected to extra column chromatography.

**General procedure 2. Synthesis of compounds 10.** A mixture of homophthalic anhydride (1 equiv) and the corresponding indolenine **9a–t** (1 equiv) was placed in a sealed screw-cap vial, dissolved in dry acetonitrile (2 mL per 1 mmol) and stirred at room temperature for the time indicated in Table 1. The reaction mixture was cooled to −14 °C, the resulting precipitate was filtered and washed with a minimum amount of cold acetonitrile to give pure compound **10**.

**General procedure 3. Synthesis of methyl esters 10'.** The Castagnoli–Cushman product **10** was dissolved in dry acetone (10 mL per 1 mmol). Methyl iodide (2.5 equiv) and K_2_CO_3_ (2.5 equiv) were added to the solution and the resulting suspension was stirred for 24 h at room temperature. The volatiles were removed in vacuo. The residue was dissolved in DCM, washed with water, brine, dried over Na_2_SO_4_ and concentrated to give crude methyl ester **10'**, which was purified by column chromatography on silica gel.

## Supporting Information

File 1 Detailed experimental procedures, analytical data and copies of ^1^H and ^13^C NMR spectra for all new compounds; crystallographic data for compounds **10** and **13e**; results of correlational and variable temperature NMR experiments.
