# Supplementary material for: A speedy route to sterically encumbered, benzene-fused derivatives of privileged, naturally occurring hexahydropyrrolo[1,2-b]isoquinoline
Source: Beilstein J Org Chem. 2017 Jul 18;13:1413–24. doi: 10.3762/bjoc.13.138 (PMC5530605; doi:10.3762/bjoc.13.138)
Supplement: File 1 — Detailed experimental procedures, analytical data and copies of 1H and 13C NMR spectra for all new compounds; crystallographic data for compounds 10 and 13e; results of correlational and variable temperature NMR experiments. [file Beilstein_J_Org_Chem-13-1413-s001.pdf]

Supporting Information

for

A speedy route to sterically encumbered, benzene-fused derivatives of privileged, naturally occurring hexahydropyrrolo[1,2-*b*]isoquinoline

Olga Bakulina, Alexander Ivanov, Vitalii Suslonov, Dmitry Dar'in and Mikhail Krasavin*,§

Address: Institute of Chemistry, Saint Petersburg State University, 26 Universitetsky prospekt, Peterhof 198504, Russia;

Email: Mikhail Krasavin - m.krasavin@spbu.ru

*Corresponding Author

§ORCID Mikhail Krasavin: 0000-0002-0200-4772

Detailed experimental procedures, analytical data and copies of ^{1}H and ^{13}C NMR spectra for all new compounds; crystallographic data for compounds 10 and 13e; results of correlational and variable temperature NMR experiments

Table of Contents

1. Experimental section..**S2**
2. NMR studies for compounds **10d** an **10e**..**S11**
3. Relative configuration determination for compounds **10k** and **10m**..................................**S17**
4. Relative configuration determination for compound **10o**..**S17**
5. Crystallographic data for compounds **10** and **13e**...**S19**
6. References……………...**S24**
7. Copies of ^{1}H and ^{13}C NMR spectra of compounds **9** and **10**...**S25**

1. EXPERIMENTAL SECTION

General information. NMR spectroscopic data were recorded with a 400 MHz (400.13 MHz for ^{1}H and 100.61 MHz for ^{13}C) and a 500 MHz (500.03 MHz for ^{1}H and 125.7 MHz for ^{13}C) spectrometers in DMSO-d_6 or in $CDCl_3$ and were referenced to residual solvent proton signals (δ_H = 7.26 and 2.50 ppm, respectively) and solvent carbon signals (δ_C = 77.2 and 39.5 ppm, respectively). Coupling constants, *J* are reported in Hz. Melting points were determined with an automated heat block instrument and are uncorrected. Mass spectra were recorded with a HRMS-ESI-qTOF spectrometer (electrospray ionization mode). X-ray single crystal analyses were performed with monochromated Mo Kα or Cu Kα radiation, respectively. Column chromatography was performed on silica gel 60 (230–400 mesh). For TLC analysis UV_{254} silica gel coated plates were used. MeCN was distilled from P_2O_5 and stored over molecular sieves 4 Å. Homophthalic anhydride was acquired from a commercial source, stored at 5 °C and used as received. All indolenines **9** were stored in sealed vials at 5 °C in the dark.

CCDC 1503093 (**13e**), 1503094 (*anti*-**10c**), 1503095 (*anti*-**10d**), 1503096 (*anti*-**10g'**), 1503097 (*anti*-**10e**), 1503098 (*anti*-**10a'**), 1503099 (*anti*-**10f**), 1503100 (*syn*-**10a'**), 1503101 (*syn*-**10f**), 1503102 (*anti*-**10e'**), 1503103 *(anti*-**10n**), 1503104 (*anti*-**10i**), 1503105 (*syn*-**10e**), 1503106 (*anti*-**10l**), 1470399 (*anti*-**10b**), 1470389 (*anti*-**10h**), 1461790 (*anti*-**10j**) contain the supplementary crystallographic data for this paper. These data can be obtained free of charge from The Cambridge Crystallographic Data Centre via http://www.ccdc.cam.ac.uk.

Indolenines 9. Indolenines **9a-b** [1], **9d-g** [1], **9h** [2], **9i** [3], **9j** [4], **9k** [5], **9p** [6] are known compounds and were prepared from the arylhydrazine hydrochlorides and respective aldehydes or ketones according to the literature protocols.

General procedure 1. Synthesis of indolenines 9c,l,n,o,q-t. To a screw-cap vial containing a suspension of the corresponding aryl hydrazine hydrochloride in glacial AcOH (15 mL) carbonyl compound (1.1 equiv) was added in one portion. The reaction mixture was stirred at 55–60 °C for 4 h (or at reflux for 2–16 h) and concentrated in vacuo at 40 °C. The residue was diluted with DCM (50 mL) and filtered through Celite. The resulting solution was passed through a short pad of silica, washed with sat. $NaHCO_3$ and water. The organic layer was dried over Na_2SO_4 and concentrated under reduced pressure to give pure indolenines **9c,l,s**. Indolenines **9n,o,q-r,t** were subjected to extra column chromatography.

5'-Fluorospiro[cyclohexane-1,3'-indole] (9c). The title compound was prepared from 4-flourophenylhydrazine hydrochloride (1.51 g, 9.3 mmol) and cyclohexanecarboxaldehyde (1.14g, 10.2 mmol) according to general procedure 1. Light yellow solid. Yield 1.39 g (74%). ^{1}H NMR (400 MHz, $CDCl_3$) δ 8.34 (s, 1H), 7.59 (dd, *J*=8.4, 4.7, 1H), 7.18 – 6.92 (m, 2H), 2.00 – 1.55 (m, 10H). ^{13}C NMR (101 MHz, $CDCl_3$) δ = 178.1 (d, *J*=3.6), 161.6 (d, *J*=244.7), 150.3, 146.7 (d, *J*=8.8), 121.8 (d, *J*=9.0), 114.3 (d, *J*=23.8), 110.0 (d, *J*=24.7), 58.3 (d, *J*=2.2), 31.6, 25.4, 23.8. HRMS (ESI) m/z $[M+H]^+$: Calcd for $C_{13}H_{14}FN$: 204.1183. Found: 204.1171.

4*a*-Methyl-2,3,4,4a-tetrahydro-1*H*-carbazole (9l) [7]. The title compound was prepared from phenylhydrazine hydrochloride (0.72 g, 5 mmol) and 2-methylcyclohexanone (0.62g, 5.5 mmol) according to general procedure 1 (reflux, 2 h). Yield 600 mg (65%). Beige solid. ^{1}H NMR (400 MHz, $CDCl_3$) δ 7.62 (d, *J*=7.7, 1H), 7.39 – 7.26 (m, 2H), 7.21 (t, *J*=7.4, 1H), 2.95 – 2.78 (m, 1H),

2.61 (td, J=13.3, 5.7, 1H), 2.38 – 2.13 (m, 2H), 1.91 – 1.60 (m, 2H), 1.43 (qd, J=13.2, 8.9, 1H), 1.33 (s, 3H), 1.18 (td, J=13.3, 4.2, 1H).

2-Methyl-2',3',5',6'-tetrahydrospiro[indole-3,4'-pyran] (9m). A mixture of 4-acetylpyrane (2.05 g, 16.0 mmol), phenylhydrazine (1.73 g, 16.0 mmol) and *p*-toluenesulfonic acid monohydrate (100 mg, 5.3 mmol) was refluxed in benzene (40 mL) with a Dean-Stark trap for 3 h. The volatiles were removed in vacuo followed by column chromatography (hexane-EtOAc from 4:1 to 5:2) affording 2.0 g of 1-phenyl-2-(1-(tetrahydro-2*H*-pyran-4-yl)ethylidene)hydrazine with impurity. The obtained compound was refluxed in 30 mL of AcOH for 3 h. The reaction mixture was concentrated, redissolved in $CHCl_3$ and washed with water. The organic layer was dried over Na_2SO_4 and concentrated to give crude **9m**, which was purified by column chromatography (hexane-EtOAc from 1:1 to 2:3). Yield 550 mg (19%). Beige solid. ^{1}H NMR (400 MHz, $CDCl_3$) δ 7.84 (d, J=7.4, 1H), 7.59 (d, J=7.6, 1H), 7.38 (t, J=7.5, 1H), 7.21 (t, J=7.5, 1H), 4.20 – 3.90 (m, 4H), 2.34 (s, 3H), 2.26 – 2.12 (m, 2H), 1.28 – 1.25 (m, 2H). ^{13}C NMR (101 MHz, $CDCl_3$) δ 185.9, 154.2, 143.8, 128.2, 124.8, 123.8, 120.6, 63.6, 55.1, 30.8, 16.2. HRMS (ESI) m/z $[M+H]^+$: Calcd for $C_{13}H_5NO$: 202.1226. Found: 202.1246.

2'-Methylspiro[cyclohexane-1,3'-indole] (9n) [8]. The title compound was prepared from phenylhydrazine hydrochloride (1.66 g, 11.5 mmol) and cyclohexylmethylketone (1.59 g, 12.6 mmol) according to general procedure 1 (reflux, 16 h). Column chromatography ($CHCl_3$) afforded 685 mg (34%) of **9n** as beige solid. ^{1}H NMR (400 MHz, $CDCl_3$) δ 7.74 (d, J=7.5, 1H), 7.58 (d, J=7.7, 1H), 7.36 (td, J=7.6, 1.1, 1H), 7.19 (td, J=7.5, 1.0, 1H), 2.31 (s, 3H), 2.06 – 1.88 (m, 3H), 1.88 – 1.73 (m, 4H), 1.60 – 1.40 (m, 1H), 1.41 – 1.23 (m, 2H).

3,3-Dimethyl-2-(*p*-tolyl)-3*H*-indole (9o) [9]. The title compound was prepared from phenylhydrazine hydrochloride (0.94 g, 6.5 mmol) and 2-methyl-1-(*p*-tolyl)propan-1-one (1.2 g, 7.2 mmol) according to general procedure 1 (reflux, 16 h). Column chromatography (eluting with $CHCl_3$-hexane 1:4) afforded 890 mg (58%) of **9o** as viscous yellowish oil. ^{1}H NMR (400 MHz, $CDCl_3$) δ 8.07 (d, J=8.2, 2H), 7.69 (d, J=7.6, 1H), 7.41 – 7.16 (m, 5H), 2.43 (s, 3H), 1.59 (s, 6H).

3,3-Dimethyl-2-propyl-3*H*-indole (9q) and 2,3-dimethyl-3-propyl-3*H*-indole [10] **(9r).** The mixture of title compounds was prepared from phenylhydrazine hydrochloride (3.74 g, 26 mmol) and 2-methylhexan-3-one (3.26 g, 28.6 mmol) according to general procedure 1. Column chromatography (eluting with $CHCl_3$-hexane from 1:2 to 1:1) afforded pure indolenines **9r** and **9s**.
(9q): Yield 892 mg (18%), orange oil. ^{1}H NMR (400 MHz, $CDCl_3$) δ 7.63 (d, J=7.7, 1H), 7.37 – 7.27 (m, 2H), 7.25 – 7.16 (m, 1H), 2.60 – 2.50 (m, 2H), 2.01 – 1.83 (m, 2H), 1.33 (s, 6H), 1.08 (t, J=7.4, 3H). ^{13}C NMR (101 MHz, $CDCl_3$) δ 191.1, 154.0, 145.8, 127.7, 125.2, 121.3, 120.2, 53.9, 31.1, 23.3, 20.0, 14.4. HRMS (ESI) m/z $[M+H]^+$: Calcd for $C_{13}H_{17}N$: 188.1434. Found: 188.1438.

(9r): Yield 550 mg (11%), orange oil. ^{1}H NMR (400 MHz, $CDCl_3$) δ = 7.53 (d, J=7.6, 1H), 7.34 – 7.10 (m, 3H), 2.25 (s, 3H), 1.91 – 1.79 (m, 1H), 1.77 – 1.67 (m, 1H), 1.28 (s, 3H), 0.87 – 0.55 (m, 5H). ^{13}C NMR (101 MHz, $CDCl_3$) 187.1, 154.2, 143.3, 127.4, 125.1, 121.6, 119.9, 57.7, 39.7, 22.6, 17.5, 15.5, 14.0. HRMS (ESI) m/z $[M+H]^+$: Calcd for $C_{13}H_{17}N$: 188.1434. Found: 188.1429.

2'-Cyclohexylspiro[cyclohexane-1,3'-indole] (9s). Phenylhydrazine hydrochloride (0.86 g, 6.5 mmol) and dicyclohexylketone (1.38 g, 7.1 mmol) were reacted according to general procedure 1 (reflux, 6h). Crystallization from hexane-DCM-EtOAc mixture (2:1:2) gave **9s**-hydrochoride as

light yellow solid. Yield 0.5 g (29%). The prepared compound (500 mg, 1.6 mmol) was dissolved in $CHCl_3$ (50 mL), washed with $NaHCO_3$ saturated solution (25 mL) and water. The organic layer was dried over Na_2SO_4 and concentrated *in vacuo* to give pure **9s**. Yield 401 mg (91%). ^{1}H NMR (400 MHz, $CDCl_3$) δ 7.70 (d, *J*=7.4, 1H), 7.61 (d, *J*=7.7, 1H), 7.32 (td, *J*=7.6, 1.1, 1H), 7.15 (td, *J*=7.5, 1.0, 1H), 2.65 – 2.53 (m, 1H), 2.05 – 1.60 (m, 14H), 1.54 – 1.42 (m, 1H), 1.42 – 1.32 (m, 3H), 1.27 – 1.17 (m, 2H). ^{13}C NMR (101 MHz, $CDCl_3$) δ 194.7, 144.1, 127.6, 124.4, 124.3, 120.4, 58.7, 38.5, 32.4, 30.1, 26.7, 26.0, 25.5, 21.8. HRMS (ESI) m/z $[M+H]^+$: Calcd for $C_{19}H_{25}N$: 268.2060. Found: 268.2051.

2'-(*p*-Tolyl)spiro[cyclohexane-1,3'-indole] (9t). The title compound was prepared from phenylhydrazine hydrochloride (0.53 g, 3.7 mmol) and cyclohexyl(*p*-tolyl)methanone (0.83 g, 4.1 mmol) according to general procedure 1 (reflux, 16 h). Column chromatography (eluting with $CHCl_3$-hexane 1:4) afforded 650 mg (64%) of **9t** as viscous yellow oil. ^{1}H NMR (400 MHz, DMSO-d_6) δ 8.07 (d, *J*=8.3, 1H), 7.87 (d, *J*=7.4, 1H), 7.63 (d, *J*=7.3, 1H), 7.39 (td, *J*=7.6, 1.0, 1H), 7.33 (d, *J*=8.1, 1H), 7.23 (td, *J*=7.5, 1.0, 1H), 2.38 (s, 3H), 2.34 – 2.21 (m, 2H), 2.02 – 1.88 (m, 3H), 1.81 – 1.72 (m, 2H), 1.65 – 1.50 (m, 1H), 1.27 – 1.21 (m, 2H).^{13}C NMR (101 MHz, DMSO-d_6) δ 182.1, 153.3, 146.3, 140.1, 130.1, 129.1, 128.4, 127.5, 124.7, 124.3, 120.7, 57.5, 30.8, 24.5, 21.2, 20.9. HRMS (ESI) m/z $[M+H]^+$: Calcd for $C_{20}H_{21}N$: 276.1747. Found: 276.1758.

General procedure 2. Synthesis of compounds 10. A mixture of homophthalic anhydride (1 equiv.) and the corresponding indolenine **9a–t** (1 equiv) was placed in a sealed screw-cap vial, dissolved in dry acetonitrile (2 mL per 1 mmol) and stirred at room temperature for time indicated in Table 1. The reaction mixture was cooled to –14 °C, the resulting precipitate was filtered and washed with minimum amount of cold acetonitrile to give pure compound **10**.

General procedure 3. Synthesis of methyl esters 10'. The Castagnoli-Cushman product **10** was dissolved in dry acetone (10 mL per 1 mmol). Methyl iodide (2.5 equiv) and K_2CO_3 (2.5 equiv) were added to the solution and the resulting suspension was stirred for 24 h at room temperature. The volatiles were removed in vacuo. The residue was dissolved in DCM, washed with water, brine, dried over Na_2SO_4 and concentrated to give crude methyl ester **10'**, which was purified by column chromatography on silica gel.

(*RS,RS*)-6'-Oxo-11',11a'-dihydro-6'*H*-spiro[cyclohexane-1,12'-indolo[1,2-*b*]isoquinoline]-11'-carboxylic acid (*anti*-10a). The title compound was prepared according to general procedure 2 (1.9 mmol scale). The product was obtained as white solid, mp 251−252 ºC (decomp.). Yield 368 mg, 56%. ^{1}H NMR (400 MHz, DMSO-d_6) δ 13.68 (s, 1H), 8.42 (d, *J* = 7.9 Hz, 1H), 8.19 – 8.00 (m, 1H), 7.74 (d, *J* = 7.6 Hz, 1H), 7.64 (td, *J* = 7.6, 1.1 Hz, 1H), 7.50 (t, *J* = 7.5 Hz, 1H), 7.31 – 7.25 (m, 2H), 7.10 (t, *J* = 7.3 Hz, 1H), 4.36 (d, *J* = 12.9 Hz, 1H), 4.32 (d, *J* = 12.8 Hz, 1H), 2.17 – 1.97 (m, 1H), 1.95 – 1.75 (m, 4H), 1.75 – 1.63 (m, 1H), 1.64 – 1.49 (m, 3H), 1.27 – 1.11 (m, 1H); ^{13}C NMR (101 MHz, DMSO-d_6) δ 172.9, 161.0, 140.7, 138.8, 136.2, 132.8, 128.6, 127.8, 127.7, 127.5, 125.7, 125.6, 123.5, 116.0, 68.7, 46.3, 46.0, 34.0, 31.2, 25.1, 22.1, 20.0.; HRMS (ESI) m/z $[M+H]^+$: Calcd for $C_{22}H_{21}NO_3$: 348.1594. Found: 348.1586.

The filtrate was concentrated and subjected to esterification according general procedure 3 and column chromatography (hexane-Me_2CO 7:1) to give some extra products ***anti*-10a'** and ***syn*-10a'**.

(*RS,RS*)-Methyl 11*a*,12,12-trimethyl-6-oxo-6,11,11*a*,12-tetrahydroindolo[1,2-*b*]isoquinoline-11-carboxylate (*anti*-10a'). Yield 84 mg, 12%. White solid, mp 158−159 ºC. ^{1}H NMR (400 MHz, $CDCl_3$) δ 8.56 (d, *J*=8.1, 1H), 8.25 (d, *J*=7.3, 1H), 7.70 (d, *J*=7.6, 1H), 7.62 – 7.39 (m, 2H), 7.32 (t, *J*=7.7, 1H), 7.16 – 6.94 (m, 2H), 4.44 (d, *J*=13.0, 1H), 4.39 (d, *J*=12.9, 1H), 3.95 (s, 3H), 2.19 – 2.01 (m, 1H), 2.01 – 1.86 (m, 2H), 1.86 – 1.71 (m, 2H), 1.70 – 1.44 (m, 4H), 1.35 – 1.18 (m, 1H); ^{13}C NMR (101 MHz, $CDCl_3$) δ 172.4, 161.9, 141.2, 138.8, 135.5, 132.7, 129.2, 128.7, 128.3, 128.1, 125.8, 125.5, 123.8, 117.18, 69.4, 52.5, 46.7, 46.7, 34.5, 31.9, 25.7, 23.0, 20.6; HRMS (ESI) m/z $[M+H]^+$: Calcd for $C_{23}H_{23}NO_3$: 362.1751. Found: 362.1758.

(*RS,SR*)-Methyl 11*a*,12,12-trimethyl-6-oxo-6,11,11*a*,12-tetrahydroindolo[1,2-*b*]isoquinoline-11-carboxylate (*syn*-10a'). Yield 50 mg, 7%. White solid, mp 152−153 ºC. ^{1}H NMR (400 MHz, $CDCl_3$) δ 8.39 (d, *J*=7.9, 1H), 8.19 (dd, *J*=6.0, 3.0, 1H), 7.55 – 7.43 (m, 2H), 7.44 – 7.35 (m, 1H), 7.33 – 7.20 (m, 2H), 7.06 (t, *J*=7.5, 1H), 4.49 (d, *J*=3.2, 1H), 4.13 (d, *J*=3.2, 1H), 3.40 (s, 3H), 1.97 – 1.44 (m, 10H); ^{13}C NMR (101 MHz, $CDCl_3$) δ 171.2, 162.6, 142.0, 139.5, 136.2, 132.5, 131.0, 129.3, 129.2, 128.3, 127.6, 123.8, 123.3, 116.0, 70.1, 52.6, 46.9, 46.8, 42.3, 32.2, 25.5, 23.6, 22.7; HRMS (ESI) m/z $[M+H]^+$: Calcd for $C_{23}H_{23}NO_3$: 362.1751. Found: 362.1765.

(*RS,RS*)-2'-Methyl-6'-oxo-11',11*a*'-dihydro-6'*H*-spiro[cyclohexane-1,12'-indolo[1,2-*b*]isoquinoline]-11'-carboxylic acid (*anti*-10b). The title compound was prepared according to general procedure 2 (1 mmol scale). The product was obtained as white solid, mp 286−287 ºC (decomp.). Yield 268 mg, 73%. ^{1}H NMR (400 MHz, DMSO-d_6) δ 13.85 (s, 1H), 8.45 (d, *J*=8.2, 1H), 8.22 (dd, *J*=7.7, 1.0, 1H), 7.79 (td, *J*=7.6, 1.3, 1H), 7.71 (s, 1H), 7.66 (t, *J*=7.5, 1H), 7.41 (d, *J*=7.7, 1H), 7.26 (d, *J*=8.1, 1H), 4.51 (d, *J*=12.9, 1H), 4.45 (d, *J*=12.9, 1H), 2.50 (s, 3H), 2.32 – 2.17 (m, 1H), 2.08 – 1.62 (m, 8H), 1.43 – 1.26 (m, 1H); ^{13}C NMR (101 MHz, DMSO-d_6) δ 173.0, 160.6, 139.0, 138.4, 136.1, 132.7, 132.6, 128.6, 127.8, 127.6, 126.3, 125.6, 115.7, 68.8, 46.3, 46.0, 34.0, 31.1, 25.16, 22.2, 21.0, 20.0; HRMS (ESI) m/z $[M+H]^+$: Calcd for $C_{23}H_{23}NO_3$: 362.1751. Found: 362.1751.

The filtrate was subjected to esterification according general procedure 3 and column chromatography (eluent – $CHCl_3$) to give some extra product methyl ester ***syn*-10b'**;

(*RS,SR*)-Methyl 2'-methyl-6'-oxo-11',11*a*'-dihydro-6'*H*-spiro[cyclohexane-1,12'-indolo[1,2-*b*]isoquinoline]-11'-carboxylate (*syn*-10b'). Viscous yellow oil. Yield 48 mg, 13%. ^{1}H NMR (400 MHz, $CDCl_3$) δ 8.32 – 8.21 (m, 1H), 8.21 – 8.08 (m, 1H), 7.54 – 7.42 (m, 2H), 7.44 – 7.35 (m, 1H), 7.15 – 7.01 (m, 2H), 4.48 (d, *J*=3.3, 1H), 4.12 (d, *J*=3.3, 1H), 3.41 (s, 3H), 2.35 (s, 3H), 1.92 – 1.46 (m, 10H); ^{13}C NMR (101 MHz, $CDCl_3$) δ 171.1, 162.0, 139.5, 139.5, 136.0, 133.2, 132.2, 130.9, 129.0, 129.0, 128.7, 127.4, 123.9, 115.6, 70.0, 52.5, 46.7, 46.6, 42.2, 32.1, 25.4, 23.5, 22.6, 21.5; HRMS (ESI) m/z $[M+H]^+$: Calcd for $C_{24}H_{25}NO_3$: 376.1907. Found: 376.1922.

2'-Fluoro-6'-oxo-11',11*a*'-dihydro-6'*H*-spiro[cyclohexane-1,12'-indolo[1,2-*b*]isoquinoline]-11'-carboxylic acid (*anti/syn*-10c). The title compound was prepared according to general procedure 2 (1.2 mmol scale). The product was obtained as white solid, mp 263−265 ºC (decomp.). Yield 295 mg, 66% (*anti/syn* = 3.3:1). Crystallizaion from MeCN gave ***anti*-10c** as a single isomer (151 mg, 34%). ^{1}H NMR (400 MHz, DMSO-d_6) δ 13.71 (s, 1H), 8.41 (dd, *J*=8.7, 5.3, 1H), 8.06 (d, *J*=7.5, 1H), 7.64 (t, *J*=7.2, 1H), 7.58 – 7.41 (m, 2H), 7.25 (d, *J*=7.6, 1H), 7.16 – 6.98 (m, 1H), 4.37 (s, 2H), 2.10 – 1.94 (m, 1H), 1.92 – 1.47 (m, 8H), 1.26 – 1.06 (m, 1H); ^{13}C NMR (101 MHz, DMSO-d_6) δ = 173.1, 161.0, 158.4 (d, J_{CF} =239.6), 141.1 (d, J_{CF}=7.7), 137.4 (d, J_{CF} =1.4), 136.3, 133.0, 128.6,

128.1, 127.8, 125.8, 117.0 (d, J_{CF} =8.3), 113.9 (d, J_{CF} =22.6), 113.3 (d, J_{CF} =24.8), 69.2, 46.5, 46.3 (d, J_{CF} =1.0), 33.9, 31.2, 25.2, 22.1, 20.2; HRMS (ESI) m/z $[M+H]^+$: Calcd for $C_{22}H_{20}FNO_3$: 366.1500. Found: 366.1509.

(*RS,RS*)-4'-Methyl-6'-oxo-11',11*a*'-dihydro-6'*H*-spiro[cyclohexane-1,12'-indolo[1,2-*b*]isoquinoline]-11'-carboxylic acid (*anti*-10d). The title compound was prepared according to general procedure 2 (3 mmol scale). The product was obtained as white solid, mp 210−212 ºC (decomp.). Yield 907 mg, 84%. ^{1}H NMR (400 MHz, DMSO-d_6) δ 8.04 (dd, *J*=7.8, 1.0, 1H), 7.61 (td, *J*=7.6, 1.3, 1H), 7.55 – 7.36 (m, 3H), 7.17 – 7.04 (m, 2H), 4.38 (d, *J*=3.3, 1H), 4.25 (d, *J*=3.3, 1H), 2.05 – 1.91 (m, 1H), 1.83 – 1.56 (m, 4H), 1.45 – 1.12 (m, 4H), 1.00 – 0.85 (m, 1H); ^{13}C NMR (101 MHz, DMSO-d_6) δ 173.5, 161.8, 141.9, 141.8, 134.9, 132.4, 129.6, 129.1, 128.5, 128.0, 127.8, 127.5, 125.7, 122.0, 71.5, 47.6, 41.7, 30.8, 29.6, 24.9, 22.4, 20.2; HRMS (ESI) m/z $[M+H]^+$: Calcd for $C_{23}H_{23}NO_3$: 362.1751. Found: 362.1759.

2',4'-Dichloro-6'-oxo-11',11*a*'-dihydro-6'*H*-spiro[cyclohexane-1,12'-indolo[1,2-*b*]isoquinoline]-11'-carboxylic acid (*anti/syn*-10e). The title compound was prepared according to general procedure 2 (1 mmol scale). The product was obtained as beige solid, yield 275 mg, 66% (*anti/syn* 0.8:1).

Single isomers of **10e** and compound **13e** were obtained via mechanical separation of monocrystals formed from the same reaction carried out without stirring (reaction conditions: 70 mg (0.28 mmol) of indolenine **9e**, 45 mg (0.28 mmol) of HPA, 2.55 mL MeCN, 5 °C, 48 h).

(*RS,RS*)-2',4'-Dichloro-6'-oxo-11',11*a*'-dihydro-6'*H*-spiro[cyclohexane-1,12'-indolo[1,2-*b*]isoquinoline]-11'-carboxylic acid (*anti*-10e). ^{1}H NMR (400 MHz, DMSO-d_6) δ 13.25 (s, 1H), 8.03 (d, *J*=7.3, 1H), 7.68 – 7.60 (m, 2H), 7.58 – 7.51 (m, 2H), 7.47 (t, *J*=7.5, 1H), 4.45 (d, *J*=2.5, 1H), 4.38 (d, *J*=2.7, 1H), 2.00 – 1.84 (m, 1H), 1.81 – 1.64 (m, 4H), 1.40 (s, 1H), 1.31 – 1.15 (m, 3H), 0.94 (d, *J*=5.3, 1H); ^{13}C NMR (101 MHz, DMSO-d_6) δ ^{13}C NMR (101 MHz, DMSO) δ 173.2, 161.8, 146.3, 140.0, 135.0, 132.9, 130.5, 128.9, 128.0, 127.7, 127.7, 127.3, 126.3, 123.6, 71.8, 49.0, 41.4, 30.0, 29.3, 24.6, 22.1, 20.0; HPLC-MS-ESI m/z $[M+H]^+$: Calcd for $C_{22}H_{19}Cl_2NO_3$: 416.0815. Found: 416.0818.

(*RS,SR*)-2',4'-Dichloro-6'-oxo-11',11*a*'-dihydro-6'*H*-spiro[cyclohexane-1,12'-indolo[1,2-*b*]isoquinoline]-11'-carboxylic acid (*syn*-10e). ^{1}H NMR (400 MHz, DMSO-d_6) δ 8.02 (d, *J*=7.1, 1H), 7.61 (td, *J*=7.5, 1.2, 1H), 7.57 – 7.48 (m, 2H), 7.40 (d, *J*=1.9, 1H), 7.33 (d, *J*=1.9, 1H), 4.69 (d, *J*=2.8, 1H), 4.26 (d, *J*=2.7, 1H), 2.09 – 1.96 (m, 1H), 1.89 – 1.33 (m, 9H); ^{13}C NMR (101 MHz, DMSO-d_6) δ 171.6, 160.3, 146.1, 138.0, 137.4, 132.5, 130.1, 129.0, 128.3, 128.2, 128.1, 127.2, 122.0, 119.3, 70.2, 46.7, 45.9, 41.2, 31.0, 24.5, 23.1, 21.6; HPLC-MS-ESI m/z $[M+H]^+$: Calcd for $C_{22}H_{19}Cl_2NO_3$: 416.0815. Found: 416.0818.

(*SR*)-4-((*RS*)-5',7'-Dichlorospiro[cyclohexane-1,3'-indolin]-2'-yl)isochro-mane-1,3-dione (13e). ^{1}H NMR (500 MHz, 4% Me_2CO-d_6 in $CDCl_3$; 5 °C) δ 8.18 (d, *J*=7.7, 1H), 7.74 (t, *J*=7.5, 1H), 7.54 (t, *J*=7.6, 1H), 7.37 (d, *J*=7.7, 1H), 7.03 (s, 1H), 6.87 (s, 1H), 4.28 (s, 1H), 4.19 (s, 1H), 3.78 (s, 1H), 2.79 – 2.67 (m, 1H), 2.06 – 1.94 (m, 1H), 1.84 – 1.72 (m, 1H), 1.69 – 1.50 (m, 4H), 1.50 – 1.36 (m, 2H), 1.29 – 1.12 (m, 1H); ^{13}C NMR (126 MHz, 4% Me_2CO-d_6 in $CDCl_3$; 5 °C) δ = 166.1, 160.8, 142.3, 140.6, 137.9, 135.6, 130.3, 128.8, 127.4, 127.0, 125.4, 123.5, 121.2, 117.2, 71.8, 51.2, 47.3, 38.6, 28.5, 25.5, 23.8, 22.7;

Methyl (11'*RS*,11a'*RS*)-2',4'-dichloro-6'-oxo-11',11a'-dihydro-6'*H*-spiro[cyclohexane-1,12'-indolo[1,2-*b*]isoquinoline]-11'-carboxylate (*anti*-10e'). The title compound was prepared *via* esterification of **anti/syn-10e** mixture (700 mg, 1.67 mmol, *anti/syn* 1:1) according to general procedure 3 and column chromatography. Yield 534 mg, 75%. Yellow foam. ^{1}H NMR (400 MHz, $CDCl_3$) δ 8.26 (dd, *J*=7.8, 1.3, 1H), 7.55 (td, *J*=7.5, 1.4, 1H), 7.51 (d, *J*=1.9, 1H), 7.46 (t, *J*=7.3, 1H), 7.38 (d, *J*=1.9, 1H), 7.34 (d, *J*=7.7, 1H), 4.43 (d, *J*=4.9, 1H), 4.26 (d, *J*=4.9, 1H), 3.83 (s, 3H), 2.04 – 1.73 (m, 4H), 1.67 – 1.15 (m, 6H); ^{13}C NMR (101 MHz, $CDCl_3$) δ 172.3, 161.7, 145.7, 139.4, 133.5, 132.8, 131.3, 129.0, 128.7, 128.5, 127.4, 126.9, 123.7, 71.5, 52.2, 49.4, 42.9, 31.5, 30.0, 25.5, 22.9, 20.5; HRMS (ESI) m/z $[M-H]^-$: Calcd for $C_{23}H_{21}Cl_2NO_3$: 428.0826. Found: 428.0833.

1'-(Methylsulfonyl)-6-oxo-11,11*a*-dihydro-6*H*-spiro[indolo[1,2-*b*]isoquinoline-12,4'-piperidine]-11-carboxylic acid (*anti/syn*-10f). The title compound was prepared according to general procedure 2 (1 mmol scale). White solid, yield 325 mg, 79% (*anti/syn* 2:1). This diastereomeric mixture was separated after esterification according to general procedure 3 and column chromatography (hexane-Me_2CO-DCM 6:2:1 to 4:2:1).

(*RS*,*RS*)-Methyl 1'-(methylsulfonyl)-6-oxo-11,11*a*-dihydro-6*H*-spiro-[indolo[1,2-*b*]isoquinoline-12,4'-piperidine]-11-carboxylate (*anti*-10f'). White solid. Yield 191 mg, 41%. Mp 205−207 ºC. ^{1}H NMR (400 MHz, $CDCl_3$) δ 8.59 (d, *J*=7.9, 1H), 8.24 (dd, *J*=7.7, 1.3, 1H), 7.64 – 7.51 (m, 2H), 7.48 (t, *J*=7.5, 1H), 7.41 – 7.30 (m, 1H), 7.19 – 6.99 (m, 2H), 4.48 (d, *J*=12.9, 1H), 4.38 (d, *J*=12.8, 1H), 3.99 (s, 3H), 3.90 – 3.82 (m, 1H), 3.81 – 3.72 (m, 1H), 3.57 (ddd, *J*=13.0, 10.1, 6.0, 1H), 3.17 (td, *J*=12.5, 2.1, 1H), 2.92 (s, 3H), 2.25 (td, *J*=12.7, 4.5, 1H), 2.09 – 1.98 (m, 2H), 1.69 (d, *J*=13.1, 1H); ^{13}C NMR (101 MHz, $CDCl_3$) δ 172.0, 161.9, 141.3, 136.5, 135.1, 133.1, 129.0, 129.0, 128.8, 128.6, 125.6, 124.8, 124.1, 117.7, 68.6, 53.0, 46.6, 44.6, 42.8, 41.0, 36.5, 33.6, 31.3; HRMS (ESI) m/z $[M+H]^+$: Calcd for $C_{23}H_{24}N_2O_5S$: 463.1298. Found: 463.1310.

(*RS*,*SR*)-Methyl 1'-(methylsulfonyl)-6-oxo-11,11*a*-dihydro-6*H*-spiro-[indolo[1,2-*b*]isoquinoline-12,4'-piperidine]-11-carboxylate (*syn*-10f'). White solid. Yield 102 mg, 22%. Mp 225−226 ºC. ^{1}H NMR (400 MHz, $CDCl_3$) δ 8.42 (d, *J*=8.0, 1H), 8.26 – 8.10 (m, 1H), 7.56 – 7.46 (m, 2H), 7.44 – 7.38 (m, 1H), 7.37 – 7.29 (m, 2H), 7.09 (t, *J*=7.4, 1H), 4.50 (d, *J*=2.5, 1H), 4.03 (d, *J*=2.9, 1H), 3.73 – 3.59 (m, 2H), 3.47 – 3.24 (m, 5H), 2.91 (s, 3H), 2.15 (t, *J*=5.5, 2H), 1.97 (t, *J*=5.3, 2H); ^{13}C NMR (101 MHz, $CDCl_3$) δ 170.6, 136.4, 135.5, 132.7, 129.4, 129.3, 129.1, 127.5, 124.0, 123.3, 116.3, 69.5, 52.8, 46.4, 44.4, 43.1, 42.5, 40.1, 35.9, 31.6; HRMS (ESI) m/z $[M+H]^+$: Calcd for $C_{23}H_{24}N_2O_5S$: 463.1298. Found: 463.1318.

(*RS*,*RS*)- and (*RS*,*SR*)-Methyl 12,12-dimethyl-6-oxo-6,11,11*a*,12-tetrahydroindolo[1,2-*b*]isoquinoline-11-carboxylates (*anti*- and *syn*-10g'). A mixture of phenylhydrazine hydrochloride (433 mg, 3 mmol) and isobutyric aldehyde (216 mg, 3 mmol) in 15 ml of glacial acetic acid was heated at 50 °C for 2 h in a sealed screw-cap vial. Next, the reaction mixture was gradually poured into ca. 100 ml of saturated aqueous $NaHCO_3$ and extracted with five 2 ml portions of $CHCl_3$. Combined organic extracts were washed with brine, dried over $MgSO_4$, filtered and used without purification. Solution of 3,3-dimethylindolenine (NMR yield 1.75 mmol, 58%) was placed in a 15 ml screw-cap vial followed by addition of homophthalic anhydride (284 mg, 1.75 mmol). The reaction mixture was stirred at room temperature for 16 h, concentrated *in vacuo* and redissolved in Me_2CO (12 mL). Methyl iodide (284 mg, 2 mmol) and K_2CO_3 (276 mg, 2 mmol) were added to the solution and stirred for 24 h at r.t. The volatiles were removed *in vacuo*, $CHCl_3$

and water were added to the residue. The organic layer was separated, washed with brine, dried over Na_2SO_4 and concentrated to give crude mixture of ***anti***- and ***syn*-10g'**. Column chromatography (hexane-Me_2CO from 7:1 to 4:1) afforded pure isomers.

***Anti*-10g':** Yield 286 mg, 51%. Yellow foam. ^{1}H NMR (400 MHz, $CDCl_3$) δ 8.42 (d, *J*=8.0, 1H), 8.26 (dd, *J*=7.6, 1.2, 1H), 7.58 – 7.35 (m, 2H), 7.34 – 7.25 (m, 1H), 7.21 (d, *J*=6.9, 1H), 7.16 – 6.98 (m, 2H), 4.49 (d, *J*=13.0, 1H), 4.33 (d, *J*=12.9, 1H), 3.94 (s, 3H), 1.43 (s, 3H), 1.27 (s, 3H); ^{13}C NMR (101 MHz, $CDCl_3$) 172.0, 161.8, 140.3, 140.1, 135.4, 132.8, 129.4, 128.8, 128.4, 128.3, 125.4, 124.8, 121.9, 116.9, 68.9, 52.6, 47.0, 43.6, 26.3, 25.0; HRMS (ESI) m/z $[M+H]^+$: Calcd for $C_{20}H_{19}NO_3$: 322.1438. Found: 322.1439.

***Syn*-10g':** Yield 202 mg, 36%. Yellow foam. ^{1}H NMR (400 MHz, $CDCl_3$) δ 8.40 (d, *J*=8.0, 1H), 8.30 – 8.09 (m, 1H), 7.53 – 7.45 (m, 2H), 7.42 – 7.36 (m, 1H), 7.32 – 7.27 (m, 1H), 7.18 (dd, *J*=7.4, 0.8, 1H), 7.10 (td, *J*=7.4, 0.9, 1H), 4.37 (d, *J*=3.7, 1H), 4.05 (d, *J*=3.7, 1H), 3.50 (s, 1H), 1.50 (s, 2H), 1.32 (s, 2H); ^{13}C NMR (101 MHz, $CDCl_3$) 171.4, 162.2, 141.1, 139.5, 135.7, 132.4, 130.9, 129.3, 129.1, 128.3, 127.4, 124.3, 122.0, 116.5, 70.0, 52.6, 46.0, 43.5, 30.5, 25.1; HRMS (ESI) m/z $[M+H]^+$: Calcd for $C_{20}H_{19}NO_3$: 322.1438. Found: 322.1445.

11*a*,12,12-Trimethyl-6-oxo-6,11,11*a*,12-tetrahydroindolo[1,2-*b*]isoquinoline-11-carboxylic acid (*anti/syn*-10h). The title compound was prepared after evaporation of reaction mixture obtained according to general procedure 2 (1.9 mmol scale) and washing the residue with hexane-CCl_4 1:1 mixture. The product was obtained as white solid. Yield 520 mg, 86% (*anti/syn* 4.3:1). Crystallization from MeCN gave **9h** as a single diastereomer. Mp 270–271 ºC (decomp.); ^{1}H NMR (400 MHz, DMSO-d_6) δ 13.59 (s, 1H), 8.26 (d, *J*=7.8, 1H), 8.08 (dd, *J*=7.7, 1.1, 1H), 7.66 (td, *J*=7.6, 1.3, 1H), 7.52 (t, *J*=7.5, 1H), 7.34 (d, *J*=7.3, 1H), 7.30 – 7.22 (m, 2H), 7.15 (td, *J*=7.4, 0.9, 1H), 4.63 (s, 1H), 1.41 (s, 3H), 1.31 (s, 3H), 1.13 (s, 3H); ^{13}C NMR (101 MHz, DMSO-d_6) δ 171.9, 161.0, 140.5, 138.3, 135.8, 132.8, 128.6, 127.7, 127.6, 127.6, 126.1, 124.5, 122.2, 116.2, 70.9, 49.5, 47.0, 27.1, 20.3, 17.2; HRMS (ESI) m/z $[M+H]^+$: Calcd for $C_{20}H_{19}NO_3$: 322.1438. Found: 322.1440.

2-Fluoro-11*a*,12,12-trimethyl-6-oxo-6,11,11*a*,12-tetrahydro-indolo[1,2-*b*]isoquinoline-11-carboxylic acid (*anti/syn*-10i). The title compound was prepared according to general procedure 2 (1 mmol scale) as a 3:1 mixture of diastereomers. Yield 244 mg, 72%; Diastereomers were separated after esterification according to general procedure 3 and column chromatography (hexane-Me_2CO 6:1 to 4:1).

(*RS*,*RS*)-Methyl 2-fluoro-11*a*,12,12-trimethyl-6-oxo-6,11,11*a*,12-tetrahydroindolo[1,2-*b*]isoquinoline-11-carboxylate (*anti*-10i'). White foam, yield 166 mg, 47% (for two steps); ^{1}H NMR (400 MHz, $CDCl_3$) δ 8.37 (dd, *J*=8.8, 4.9, 1H), 8.23 (dd, *J*=7.6, 1.4, 1H), 7.53 (td, *J*=7.5, 1.5, 1H), 7.46 (t, *J*=7.5, 1H), 7.05 (d, *J*=7.6, 1H), 6.96 (td, *J*=8.9, 2.7, 1H), 6.89 (dd, *J*=8.1, 2.6, 1H), 4.65 (s, 1H), 3.95 (s, 3H), 1.36 (s, 3H), 1.34 (s, 3H), 1.27 (s, 3H); ^{13}C NMR (101 MHz, $CDCl_3$) δ 171.4, 161.8, 160.3 (d, J_{CF}=243.4), 142.7 (d, J_{CF} =7.5), 135.2, 134.9 (d, J_{CF} =2.1), 132.8, 128.9, 128.7, 128.1, 126.1, 118.6 (d, J_{CF} =8.1), 114.4 (d, J_{CF} =22.8), 109.3 (d, J_{CF} =24.2), 72.1, 52.4, 50.1, 47.6 (d, J_{CF} =1.6), 27.4, 20.4, 17.4; HRMS (ESI) m/z $[M+H]^+$: Calcd for $C_{21}H_{20}FNO_3$: 354.1492. Found: 354.1500.

(*RS,SR*)-Methyl 2-fluoro-11*a*,12,12-trimethyl-6-oxo-6,11,11*a*,12-tetrahydroindolo[1,2-*b*]isoquinoline-11-carboxylate (*syn*-10i'). White foam, yield 46 mg, 13% (for two steps); ^{1}H NMR (400 MHz, $CDCl_3$) δ 8.39 (dd, *J*=8.8, 4.9, 1H), 8.25 (dd, *J*=6.4, 2.5, 1H), 7.54 – 7.41 (m, 2H), 7.39 – 7.29 (m, 1H), 6.96 (td, *J*=8.9, 2.6, 1H), 6.86 (dd, *J*=8.1, 2.6, 1H), 3.96 (s, 1H), 3.52 (s, 3H), 1.49 (s, 3H), 1.27 (s, 3H), 1.12 (s, 3H); ^{13}C NMR (101 MHz, $CDCl_3$) δ 172.2, 162.1, 160.1 (d, J_{CF} =242.7), 141.8 (d, J_{CF} =7.4), 135.9, 135.7 (d, J_{CF} =1.9), 132.5, 129.7, 129.4, 128.8, 127.5, 118.2 (d, J_{CF} =8.0), 114.4 (d, J_{CF} =22.7), 109.2 (d, J_{CF} =24.2), 70.8, 52.6, 51.7, 48.1 (d, J_{CF} =1.6), 28.2, 23.9, 21.9; HRMS (ESI) m/z $[M+H]^+$: Calcd for $C_{21}H_{20}FNO_3$: 354.1492. Found: 354.1499.

(*RS,RS*)-11*a*,12,12-Trimethyl-2-nitro-6-oxo-6,11,11*a*,12-tetrahydro-indolo[1,2-*b*]isoquinoline-11-carboxylic acid (*anti*-10j). The title compound was prepared according to general procedure 2 (1 mmol scale). The product was obtained as light yellow solid, mp 294−296 ºC (decomp.). Yield 268 mg, 73%. ^{1}H NMR (400 MHz, DMSO-d_6) δ 13.70 (s, 1H), 8.42 (d, *J* = 8.8 Hz, 1H), 8.32 – 8.16 (m, 2H), 8.11 (dd, *J* = 7.7, 1.1 Hz, 1H), 7.70 (td, *J* = 7.6, 1.4 Hz, 1H), 7.54 (t, *J* = 7.6 Hz, 1H), 7.27 (d, *J* = 7.7 Hz, 1H), 4.75 (s, 1H), 1.49 (s, 3H), 1.38 (s, 3H), 1.18 (s, 3H); ^{13}C NMR (101 MHz, DMSO-d_6) δ 171.67, 161.8, 143.9, 143.8, 142.1, 136.1, 133.5, 128.1, 127.9, 127.8, 126.3, 124.9, 118.3, 116.0, 72.1, 49.2, 47.1, 26.7, 19.9, 17.4; HRMS (ESI) m/z $[M+H]^+$: Calcd for $C_{20}H_{18}N_2O_5$: 367.1288. Found: 367.1296.

2-Hydroxy-11*a*,12,12-trimethyl-6-oxo-6,11,11*a*,12-tetrahydro-indolo[1,2-*b*]isoquinoline-11-carboxylic acid (*anti/syn*-10k). The title compound was prepared according to general procedure 2 (1 mmol scale). The product was obtained as a white solid. Yield 192 mg, 57% (*anti/syn* 6.5:1). Crystallization from MeCN gave pure *anti*-isomer for analysis. ^{1}H NMR (400 MHz, DMSO-d_6) δ 13.54 (s, 1H), 9.33 (s, 1H), 8.16 – 7.98 (m, 2H), 7.62 (td, *J* = 7.6, 1.3 Hz, 1H), 7.49 (t, *J* = 7.5 Hz, 1H), 7.22 (d, *J* = 7.7 Hz, 1H), 6.71 (d, *J* = 2.4 Hz, 1H), 6.64 (dd, *J* = 8.6, 2.4 Hz, 1H), 4.56 (s, 1H), 1.35 (s, 3H), 1.29 (s, 3H), 1.11 (s, 4H); ^{13}C NMR (101 MHz, DMSO-d_6) δ = 172.0, 160.0, 154.7, 142.1, 135.5, 132.4, 130.5, 128.7, 127.5, 127.4, 126.0, 117.2, 113.5, 109.2, 70.9, 49.6, 47.0, 27.0, 20.2, 17.1; HRMS (ESI) m/z $[M+H]^+$: Calcd for $C_{20}H_{19}NO_4$: 338.1387. Found: 338.1397.

(4*aRS*,15*SR*,15*aSR*)-4*a*-Methyl-10-oxo-2,3,4,4*a*,10,15-hexahydro-1*H*-isoquinolino[3,2-*k*]carbazole-15-carboxylic acid (*anti*-10l). The title compound was prepared according to general procedure 2 (1 mmol scale). The product was obtained as white solid, mp 253−255 ºC (decomp.). Yield 280 mg, 81%. ^{1}H NMR (400 MHz, DMSO-d_6) δ 13.59 (s, 1H), 8.32 (d, *J*=7.6, 1H), 8.07 (dd, *J*=7.7, 1.2, 1H), 7.63 (td, *J*=7.6, 1.4, 1H), 7.49 (t, *J*=7.5, 1H), 7.38 – 7.21 (m, 3H), 7.16 (td, *J*=7.4, 1.0, 1H), 4.68 (s, 1H), 2.25 – 2.13 (m, 2H), 1.74 (td, *J*=13.8, 4.0, 1H), 1.57 – 1.42 (m, 3H), 1.33 (s, 3H), 1.20 – 1.03 (m, 4H); ^{13}C NMR (101 MHz, DMSO-d_6) δ 172.4, 161.2, 139.4, 138.9, 135.7, 132.6, 128.5, 127.6, 127.5, 124.5, 122.1, 116.8, 69.8, 49.4, 47.8, 30.6, 30.0, 27.2, 20.8, 20.2; HRMS (ESI) m/z $[M+H]^+$: Calcd for $C_{22}H_{21}NO_3$: 370.1414. Found: 370.1421.

11*a*-Methyl-6-oxo-2',3',5',6',11,11*a*-hexahydro-6*H*-spiro-[indolo[1,2-*b*]isoquinoline-12,4'-pyran]-11-carboxylic acid (*anti/syn*-10m). The title compound was prepared according to general procedure 2 (1.4 mmol scale). The product was obtained as white solid, mp 252−254 ºC. Yield 436 mg, 79% (*anti/syn* = 6:1). Crystallizaion from MeCN gave ***anti*-10m** as a single isomer (289 mg, 52%). ^{1}H NMR (400 MHz, DMSO-d_6) δ 13.72 (s, 1H), 8.42 (d, *J*=7.8, 1H), 8.08 (dd, *J*=7.6, 0.9, 1H), 7.87 (d, *J*=7.5, 1H), 7.67 (td, *J*=7.6, 1.2, 1H), 7.52 (t, *J*=7.5, 1H), 7.32 (t, *J*=7.5, 1H), 7.24 (d, *J*=7.7, 1H), 7.15 (t, *J*=7.2, 1H), 4.64 (s, 1H), 4.23 (td, *J*=12.4, 2.7, 1H), 3.95 (dd, *J*=12.1, 6.1, 1H), 3.75 – 3.72 (m, 1H), 3.52 (t, *J*=11.5, 1H), 2.36 (td, *J*=12.6, 4.3, 1H), 1.99 (td, *J*=13.2, 6.5, 1H), 1.88

– 1.84 (m, 1H), 1.42 – 1.39 (m, 1H); ^{13}C NMR (101 MHz, DMSO-d_6) δ = 171.8, 161.2, 139.3, 137.5, 135.6, 132.9, 128.4, 127.7, 127.7, 127.7, 125.9, 125.9, 123.6, 116.5, 71.6, 64.0, 62.0, 49.5, 47.8, 32.6, 29.2, 15.7; HRMS (ESI) m/z [M+H]$^+$: Calcd for $C_{22}H_{21}NO_4$: 386.1363. Found: 386.1356.

(*RS*,*RS*)-11*a*'-Methyl-6'-oxo-11',11*a*'-dihydro-6'*H*-spiro[cyclohexane-1,12'-indolo[1,2-*b*]isoquinoline]-11'-carboxylic acid (*anti*-10n). The title compound was prepared according to general procedure 2 (0.9 mmol scale). The product was obtained as white solid, mp 239−241 ºC (decomp.). Yield 336 mg, 93%. ^{1}H NMR (400 MHz, DMSO-d_6) δ 13.65 (s, 1H), 8.38 (d, *J*=7.4, 1H), 8.07 (dd, *J*=7.7, 1.1, 1H), 7.71 (d, *J*=7.4, 1H), 7.65 (td, *J*=7.6, 1.4, 1H), 7.50 (t, *J*=7.5, 1H), 7.32 – 7.25 (m, 1H), 7.22 (d, *J*=7.7, 1H), 7.11 (td, *J*=7.5, 0.9, 1H), 4.63 (s, 1H), 2.17 – 1.92 (m, 3H), 1.88 – 1.74 (m, 2H), 1.64 (td, *J*=13.5, 5.5, 1H), 1.52 (d, 2H), 1.42 – 1.28 (m, 1H), 1.24 – 1.13 (m, 1H), 1.09 (s, 3H); ^{13}C NMR (101 MHz, DMSO-d_6) δ 172.1, 161.1, 139.3, 138.5, 135.8, 132.3, 128.4, 127.6, 127.4, 126.1, 126.0, 123.5, 116.4, 71.9, 49.8, 49.3, 32.7, 28.3, 25.1, 22.8, 20.7, 16.2; HRMS (ESI) m/z [M+H]$^+$: Calcd for $C_{23}H_{23}NO_3$: 384.1570. Found: 384.1558.

(*RS*,*SR*)-12,12-Dimethyl-6-oxo-11*a*-(*p*-tolyl)-6,11,11*a*,12-tetrahydroindolo[1,2-*b*]isoquinoline-11-carboxylic acid (*anti*-10o). The title compound was prepared according to general procedure 2 (0.9 mmol scale). The product was obtained as white solid, mp 236−237 ºC (decomp.). Yield 208 mg, 52%. ^{1}H NMR (400 MHz, DMSO-d_6) δ 13.64 (s, 1H), 8.38 (d, *J*=7.9, 1H), 7.77 (d, *J*=7.1, 1H), 7.50 (t, *J*=7.1, 1H), 7.37 – 7.31 (m, 2H), 7.30 – 7.21 (m, 2H), 7.19 – 7.06 (m, 2H), 6.77 (d, *J*=8.3, 2H), 5.26 (s, 1H), 2.04 (s, 3H), 1.47 (s, 3H), 1.15 (s, 3H); ^{13}C NMR (101 MHz, DMSO-d_6) δ 172.0, 162.5, 141.0, 140.4, 136.7, 136.2, 136.0, 132.4, 129.7, 128.0, 127.9, 127.5, 127.0, 125.27, 124.7, 122.7, 114.7, 77.6, 53.0, 49.0, 28.6, 22.9, 20.2; HRMS (ESI) m/z [M+H]$^+$: Calcd for $C_{26}H_{23}NO_3$: 398.1751. Found: 398.1762.

11a,12,12-Trimethyl-11*a*,12-dihydroindolo[1,2-*b*]isoquinolin-6(11*H*)-one (14h). Compound ***anti/syn*-10h** (93 mg, 0.29 mmol, 4.3:1) was dissolved in 1 mL of hexamethylphosphoramide and stirred at 205−210 °C for 1h in air. After complete consumption of starting material (controlled by TLC) the reaction mixture was diluted with 50 mL of EtOAc and washed with 5×20 mL of water. The organic layer was washed with brine, dried over Na_2SO_4 and concentrated under reduced pressure to give pure **14h** (67 mg, 83%). Yellow oil. ^{1}H NMR (400 MHz, DMSO-d_6) δ 8.22 (d, *J*=7.8, 1H), 8.03 (dd, *J*=7.6, 0.9, 1H), 7.56 (td, *J*=7.4, 1.3, 1H), 7.45 – 7.40 (m, 2H), 7.33 (dd, *J*=7.4, 0.8, 1H), 7.25 (td, *J*=7.8, 1.3, 1H), 7.12 (td, *J*=7.4, 1.0, 1H), 3.43 (d, *J*=15.6, 1H), 2.99 (d, *J*=15.7, 1H), 1.40 (s, 3H), 1.16 (s, 3H), 0.97 (s, 3H); ^{,13}C NMR (101 MHz, $CDCl_3$) δ 162.4, 140.4, 139.7, 137.1, 132.3, 129.3, 128.4, 128.3, 127.9, 127.2, 124.4, 121.9, 117.4, 69.2, 46.6, 34.3, 28.1, 20.9, 20.2; HRMS (ESI) m/z [M+H]$^+$: Calcd for $C_{19}H_{19}NO$: 278.1539. Found: 278.1537.

2. NMR studies for compounds 10d and 10e

In order to rationalize the inconsistency between $^{3}J_{HH}$-values and the value of dihedral angle in crystal form for compounds **10d** and **10e** we performed a series of NMR experiments. We reasoned that such a phenomenon could be explained by distorted molecular geometry (see section 5, crystallographic data) and different conformer population in the solution compared to solid state.

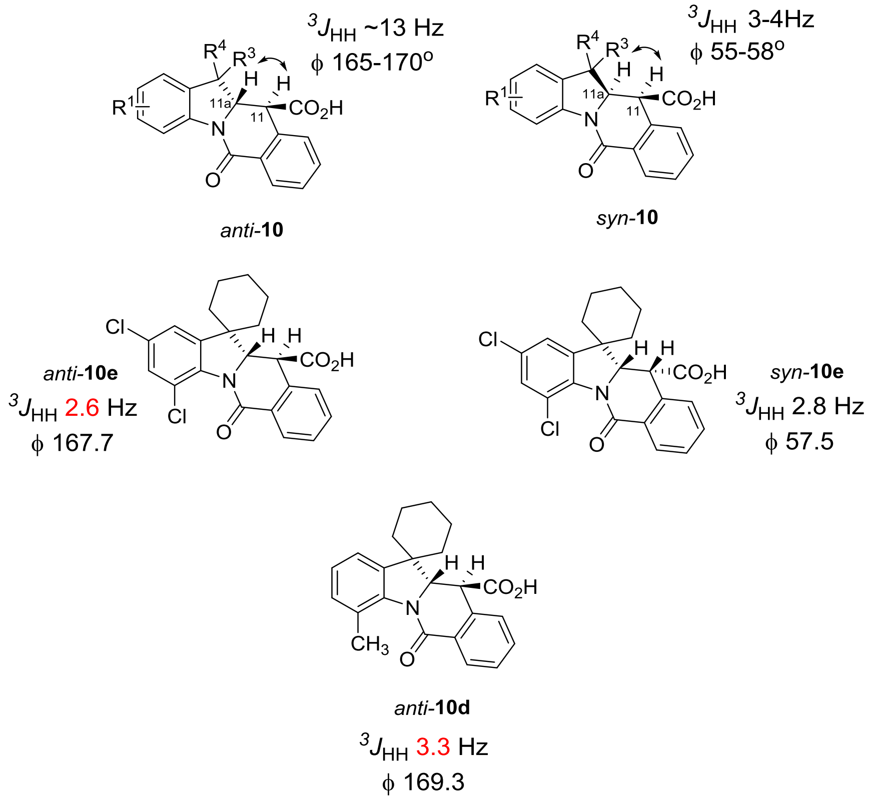

For compound **10e** two sets of H-NMR spectra were recorded: at temperatures varying from 293 to 183 FIGURE K in Me_2CO-d_6 (mixture *anti/syn* 1:1; Figure S1) and from 293 to 393K in DMSO-d_6 (*anti*-**10e**; Figure S2). We observed decoalescence for the signals of H^{11} and H^{11a} atoms for both diastereomers at 223K (Figure S1) along with new set of signals arising: δ_H = 4.56 and 4.42 ppm. It was not possible to identify these new signals by ^{1}H-NMR spectrum only, due to their very low intensity, caused by small relative content of corresponding molecules. Nevertheless correlation spectroscopy provided some useful information about these sets of signals. COSY spectrum proved this pair of protons with chemical shifts 4.56 and 4.42 ppm to be a spin system (Figure S3). ROESY spectrum recorded for compound *anti/syn*-**10e** at 183K (Figure S4) revealed exchange cross-peaks between this new spin system and spin system of compound *syn*-**10e** (δ_H 4.94 and 4.59 ppm). In addition this ROESY spectrum demonstrated another two exchange cross-peaks (δ_H 5.08-4.72 and 4.80-4.51 ppm). The new signals (δ_H 5.08 and 4.80 ppm) exchanged with signals of *anti*-**10e** (δ_H 4.72 and 4.51 ppm). These facts may indicate that two new sets of signals observed at low temperatures belong to minor conformers of different isomers of **10e**. In addition we observed variation in $J_{H11\text{-}H11a}$ coupling constant (*anti*-**10e**) from 2.6 to 0.7 Hz at temperature decrease (Figure S1) and from 2.9 to 3.7 Hz under heating (Figure S2). In the first case variation can be explained by increasing amount of major (thermodinamycally more stable) conformer with small *J*-value. Variation in the second case correlates with increasing amount of thermodinamycally less stable conformer with *J*-constant about 13 Hz, which is major conformer for all other cases (*anti*-

10a-c,f,g). A similar series of NMR-experiments was carried out for compound *anti*-**10d** – H-NMR spectra were recorded from 292 to 182K in Me_2CO-d_6 (Figure S5) and from 293 to 393K in DMSO-d_6 (Figure S6). In this case $J_{H11\text{-}H11a}$ coupling constant varied from 3.6 to 1.5 Hz under cooling and from 3.3 to 4.0 Hz under heating. *J*-values were obtained from *J*-resolved experiments.

TableS1. Measured dihedral angles for $C^{11}H$-$C^{11a}H$ fragments for compounds **10** and corresponding ^3J- coupling constants.

Compound	Angle[a] $C^{11}H$-$C^{11a}H$, °	$J_{C11H\text{-}C11aH}$, Hz	Compound	Angle[a] $C^{11}H$-$C^{11a}H$, °	$J_{C11H\text{-}C11aH}$, Hz
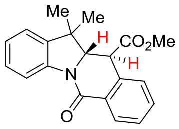 *anti*-**10g'**	168.7	13.0	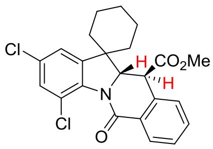 *anti*-**10e'**	94.2	4.8
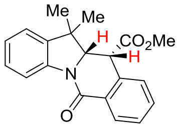 *syn*-**10g'**	ND	3.7	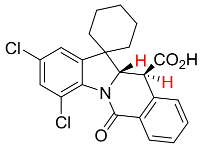 *anti*-**10e**	166.7	2.6
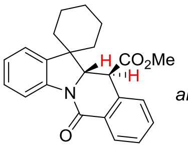 *anti*-**10a'**	164.7	13.0 (12.9, for corresponding acid)	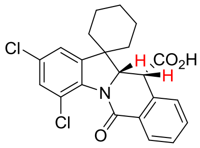 *syn*-**10e**	57.5	2.8
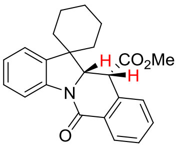 *syn*-**10a'**	58.1	3.2 (3.0, for corresponding acid)	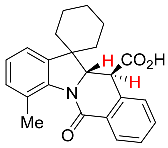 *anti*-**10d**	169.3	3.3
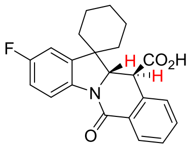 *anti*-**10c**	169.9	13.6	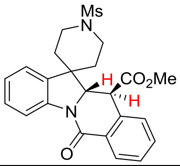 *anti*-**10f'**	171.6	12.9 (12.8, for corresponding acid)
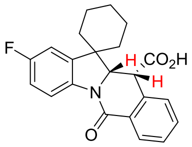 *syn*-**10c**	ND	2.8	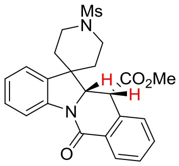 *syn*-**10f'**	55.5	2.9 (3.2, for corresponding acid)
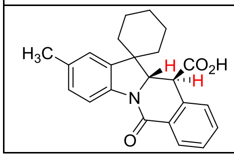 *anti*-**10b**	170.3	12.9	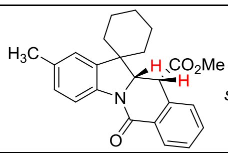 *syn*-**10b'**	ND	3.3

[a] Data obtained from X-ray single crystal analysis

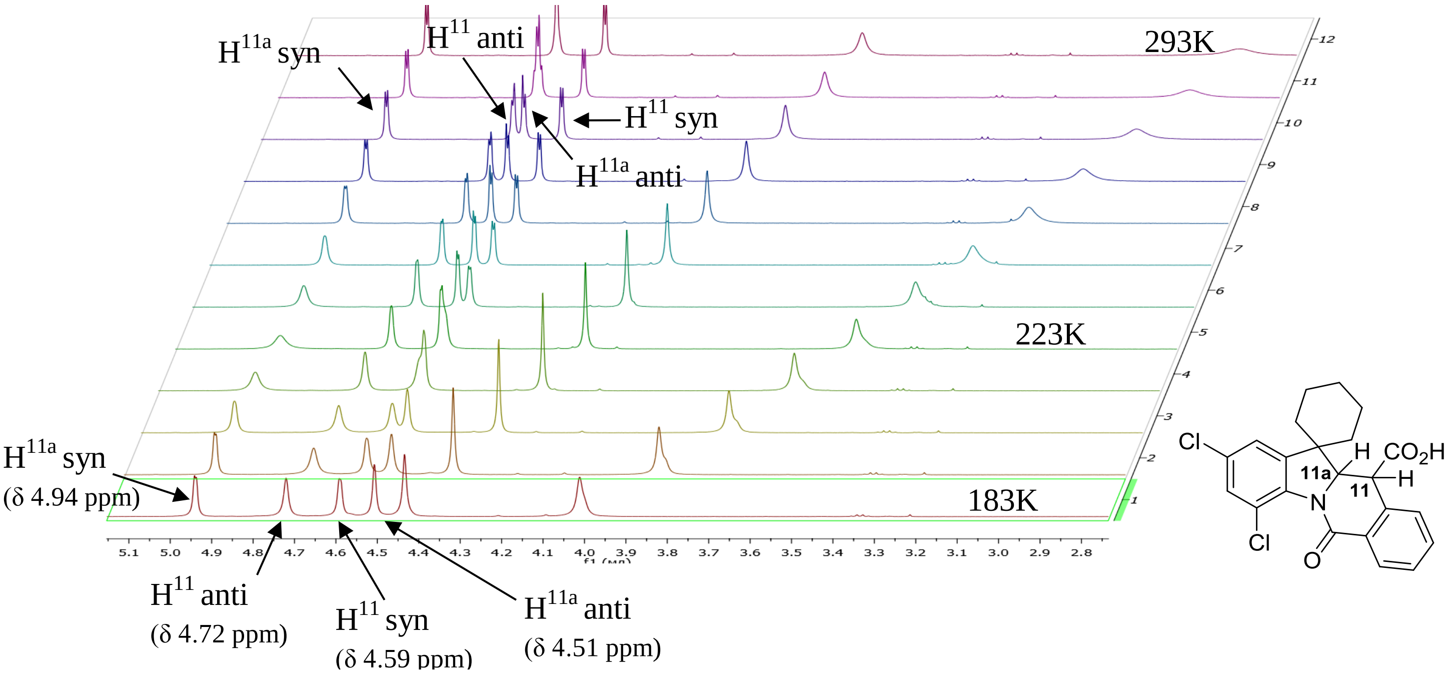

Figure S1: Fragments of H-NMR spectra of compound **10e** (*anti/syn* 1:1) at 293 K (top) – 183 K (bottom) in Me_2CO-d_6.

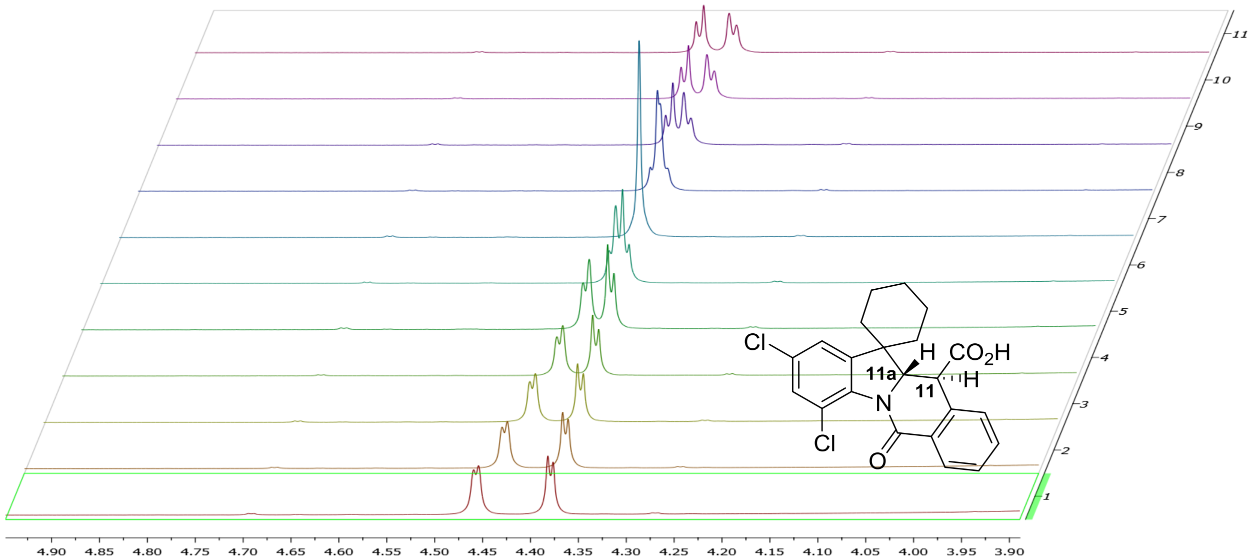

Figure S2: Fragments of H-NMR spectra of compound *anti*-**10e** at 393K (top) – 293K (bottom) in DMSO-d_6

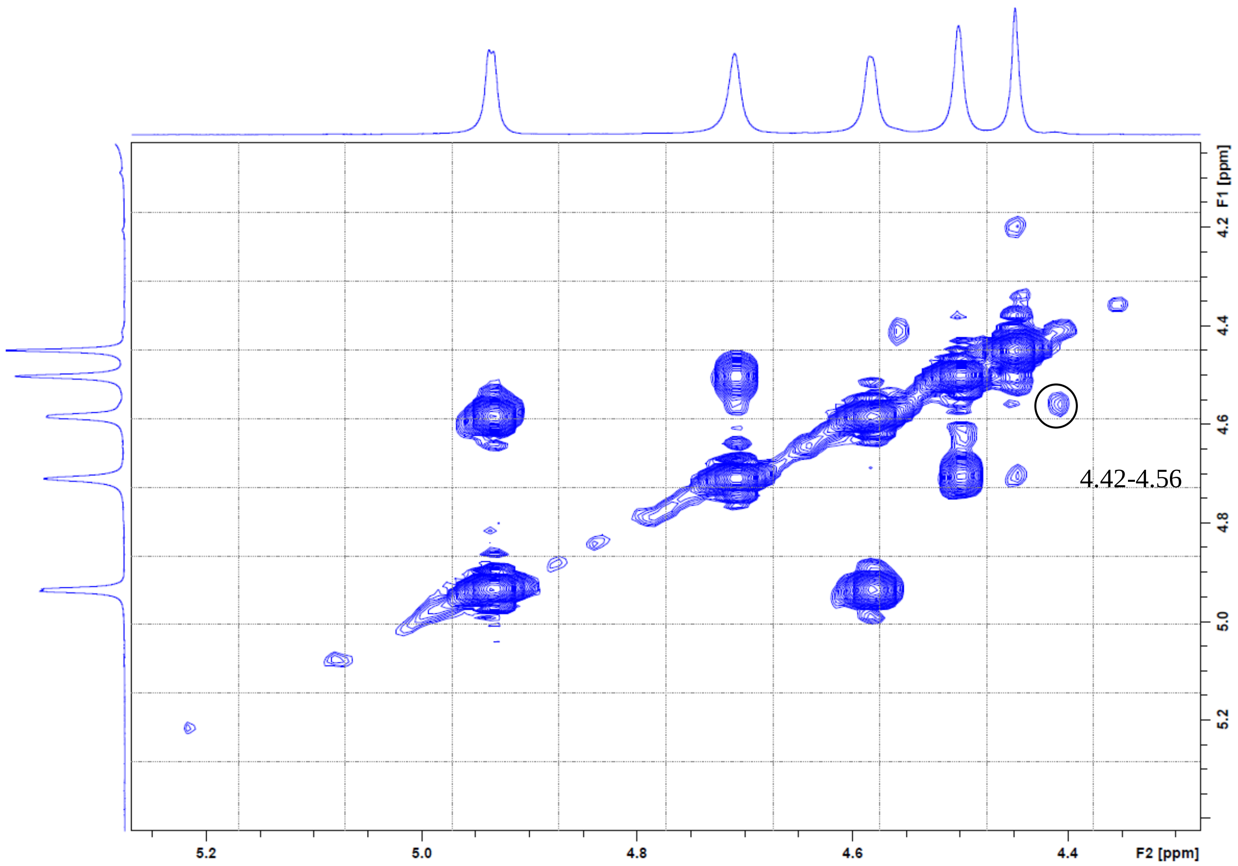

Figure S3: Fragment of COSY spectrum for compound **10e** (*anti/syn* 1:1) at 183K in Me_2CO-d_6

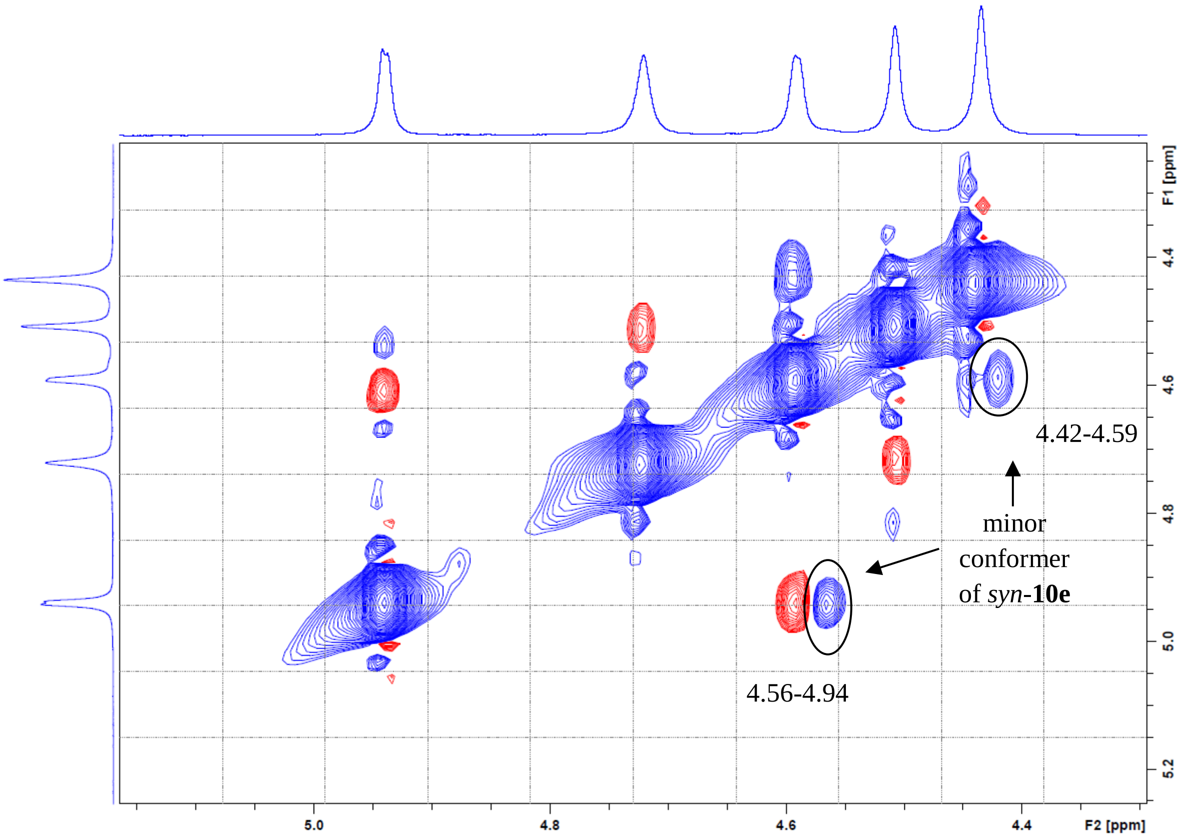

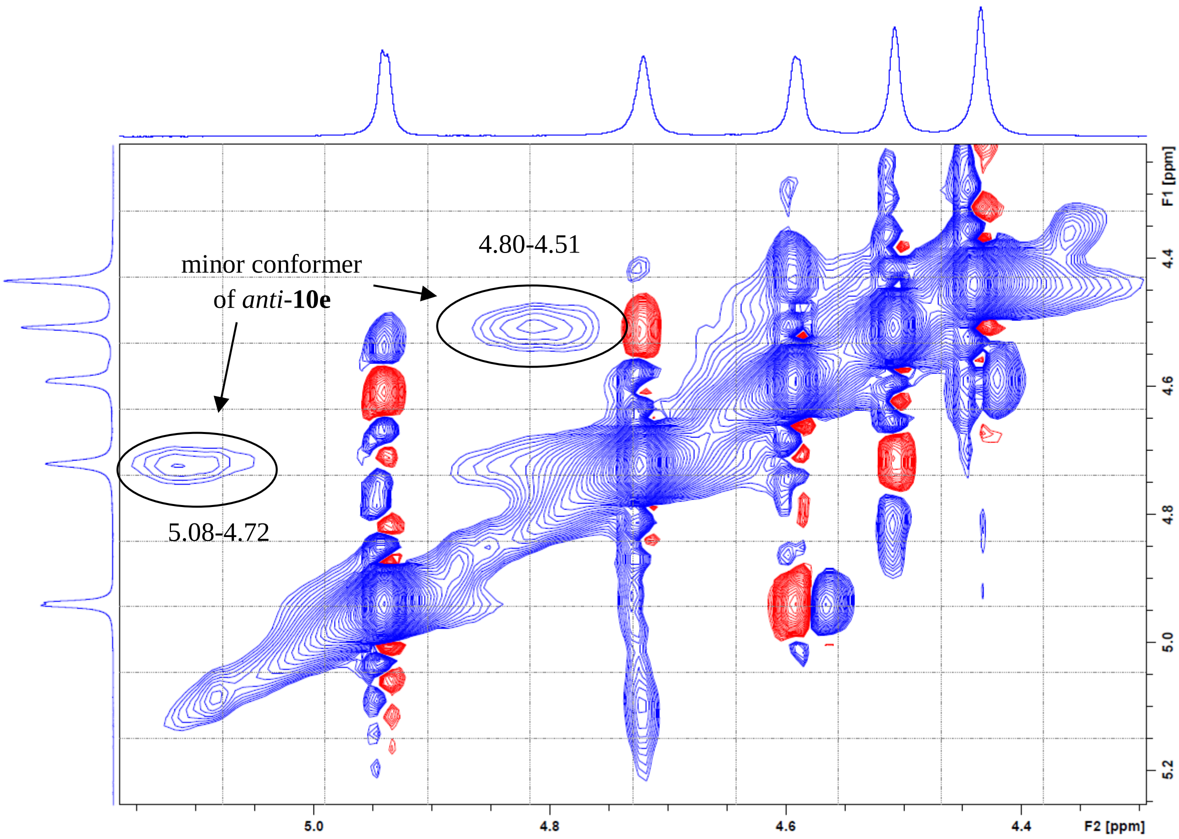

Figure S4: Fragment of ROESY spectrum for compound **10e** (*anti/syn* 1:1) at 183K in Me_2CO-d_6 with different contour levels

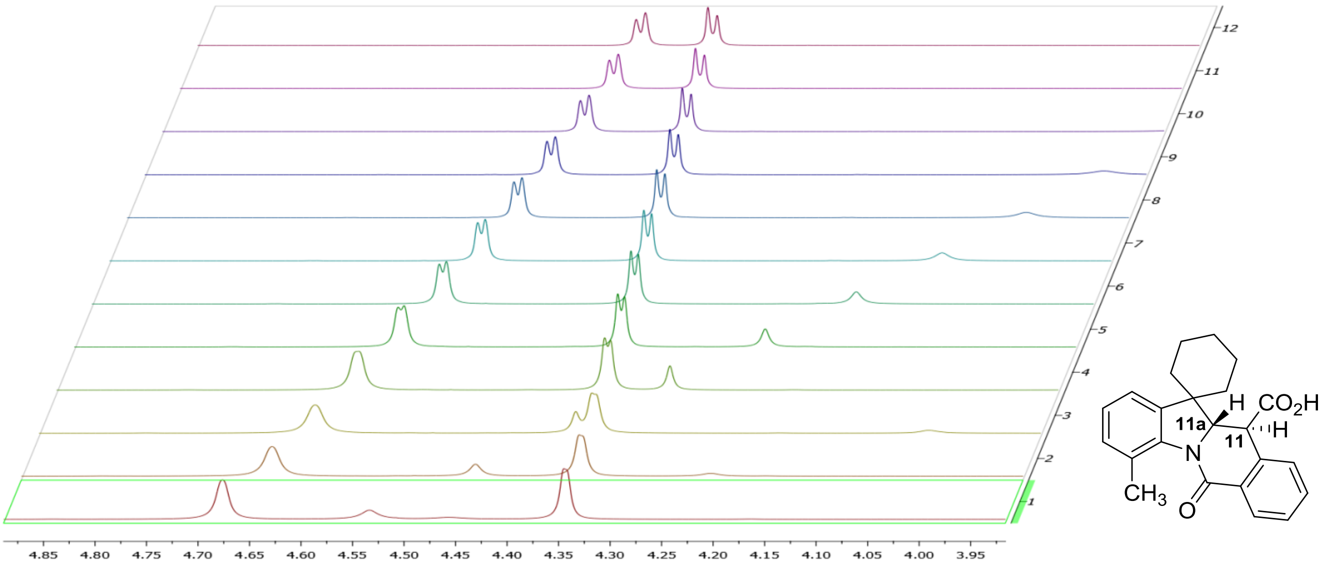

Figure S5: Fragments of H-NMR spectra of compound *anti*-**10d** at 293 K (top) – 183 K (bottom) in Me_2CO-d_6

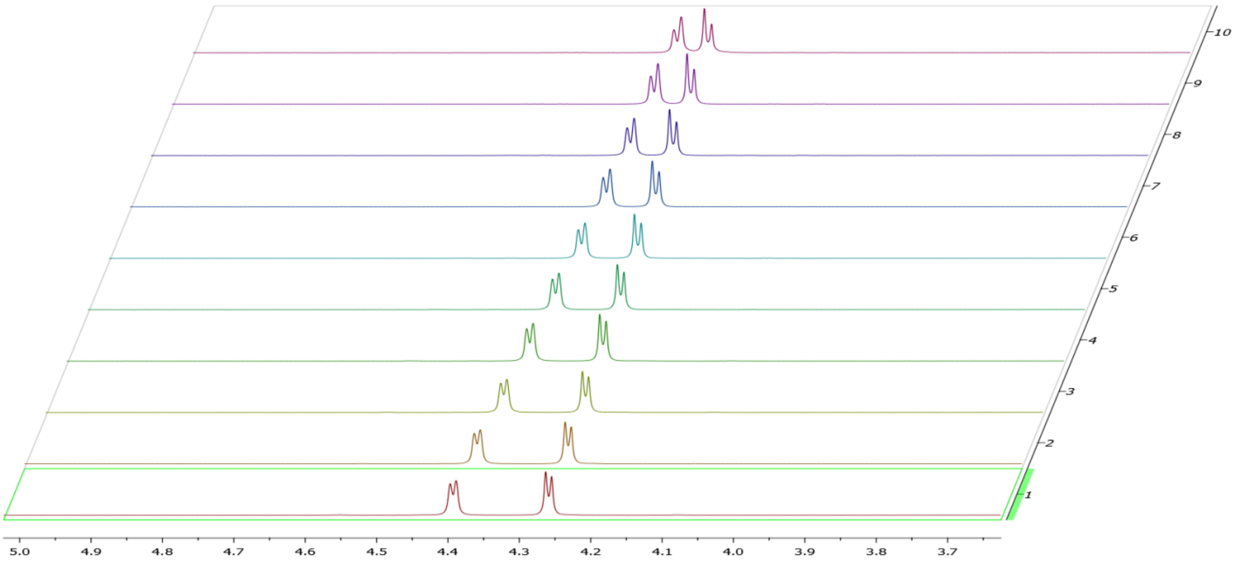

Figure S6: Fragments of H-NMR spectra of compound *anti*-**10d** at 393K (top) – 293K (bottom) in DMSO-d_6

3. Relative configuration determination for compounds 10k and 10m

^{13}C NMR spectra of **10h–k,m,n** major isomers revealed chemical shifts of carbon atom in CH_3^{11a}-group to be about 16–17 ppm, while for minor isomers of **10h–k** these chemical shifts lie within 23–24 ppm (Table S2). According to X-ray major isomers of **10h–i,n** have *anti*-configuration. Thus the values of ^{13}C chemical shifts of CH_3^{11a}-groups can serve as criterion for relative configuration assignment in CCR products **10**, derived from 2-methylindolenines. The configuration of **10k** and **10m** major isomers was determined to be *anti*- according to this criterion: $\delta C(CH_3^{11a})$= 17.1 and 15.7 ppm respectively.

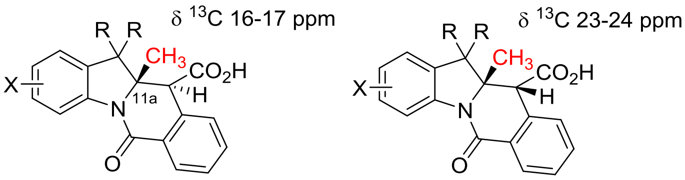

Table S2. ^{13}C chemical shifts δ,ppm of C^{11a}-methyl group for compounds **10**.

Compound	$\delta C^{11a\text{-}Me}$ (*anti-*)	$\delta C^{11a\text{-}Me}$ (*syn-*)	Compound	$\delta C^{11a\text{-}Me}$ (*anti-*)	$\delta C^{11a\text{-}Me}$ (*syn-*)
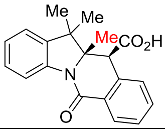 **10h**	17.2	23.4	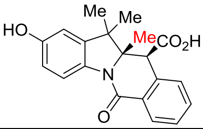 **10k**	17.1	23.9
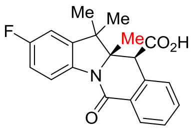 **10i**	17.7	23.8	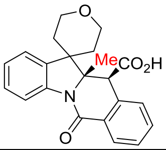 **10m**	15.7	-
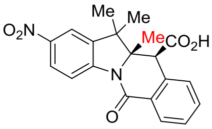 **10j**	17.4	23.3	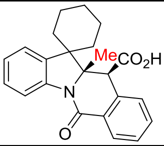 **10n**	16.2	-

4. Relative configuration determination for compound 10o

Relative configuration of 11a-(*p*-tolyl)-substituted compound **10o** was assigned to be *(RS, SR)*- on the basis of NOESY spectrum, which showed one cross peak from *ortho*-protons of *p*-tolyl moiety to methyl group in position 12 and another cross peak from H^{11} to the second methyl group in position 12. The value of observed coupling constant between C_{ipso}(*p*-tolyl) and H^{11} $^3J_{CH}$ 6.3 Hz also confirms this assignment (expected value of $^3J_{CH}$ for staggered conformation of C^{ipsoAr}–C^{11a}–C^{11}–H^{11} fragment (*anti*-isomer) is 6-8 Hz and 1-3 Hz for gauche-conformation (*syn*-isomer); Figure S7–9) [11,12].

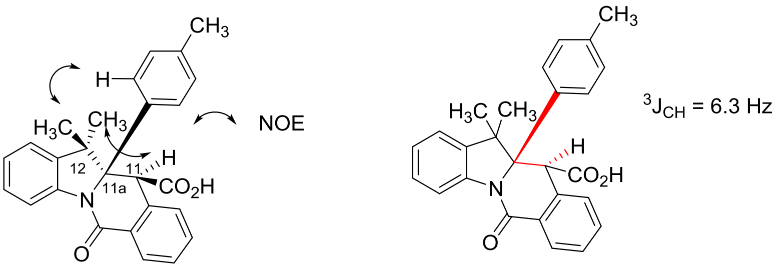

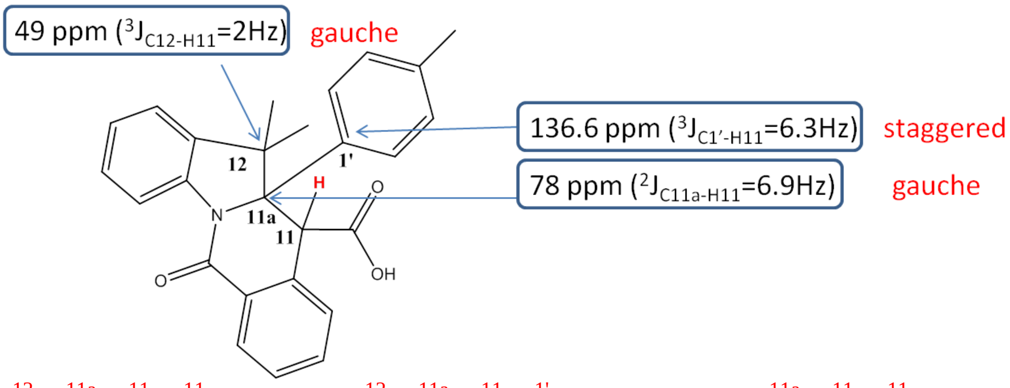

C^{12}-C^{11a}-C^{11}-H^{11} – gauche, C^{12}-C^{11a}-C^{11}-C$^{1'}$ - staggered, N-C^{11a}-C^{11}-H^{11} - gauche

Figure S7 Measured coupling constants $J_{C\text{-}H}{}^{11}$ for compound **10o**.

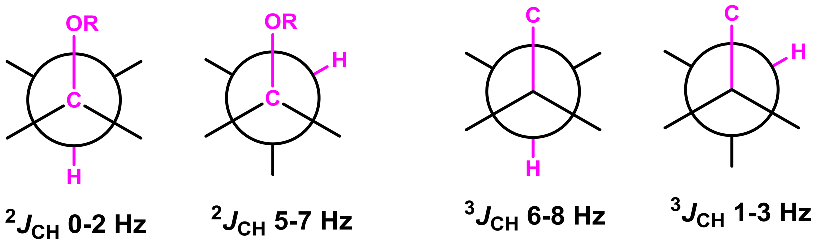

Figure S8 Reported values of coupling constants $^3J_{C\text{-}H}$ and $^2J_{C\text{-}H}$ for systems with β-heteroatom[1].

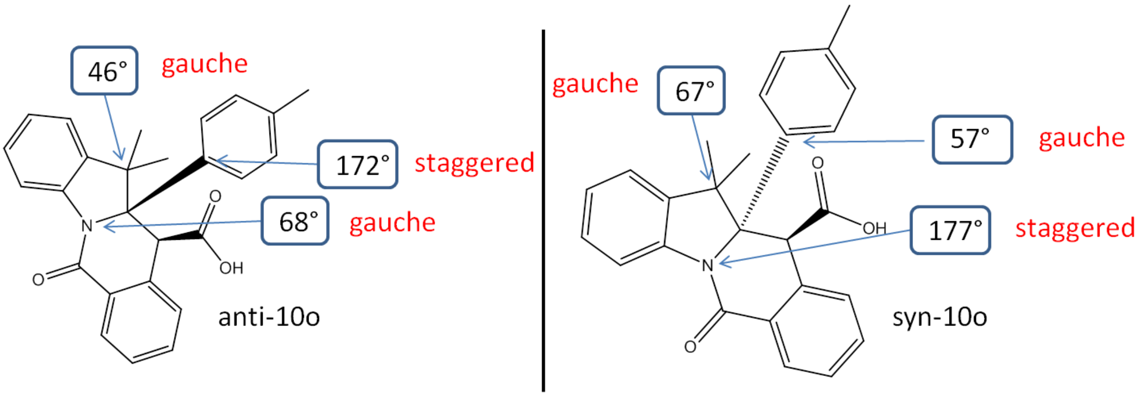

Figure S9 Calculated dihedral angles (MM2; ChemBioOffice 2014) for *anti*- and *syn*-isomers of compound **10o.**

5. Crystallographic data for compounds 10 and 13e.

X-ray single crystal analyses were performed on Agilent Technologies «Xcalibur» and «Supernova» diffractometers with monochromated Mo Kα or Cu Kα radiation, respectively. Crystals were measuring at the temperature of 100 K for all samples, except *anti*-**10f'**, which destroyed at lower temperature. The structures has been solved by the Superflip [13] and ShelXS [14] structure solution programs using Charge Flipping and Direct Methods, respectively, and refined with the ShelXL [15] refinement incorporated in the OLEX2 program package [16]. The structure *anti*-**10e'** contains disordered DMSO on threefold axis. Electron density from this solvent has been suppressed using SQUEEZE/PLATON [17] program. CCDC 1503093-1503106, 1470399, 1470389, 1461790 contain the supplementary crystallographic data for this paper. These data can be obtained free of charge from The Cambridge Crystallographic Data Centre via http://www.ccdc.cam.ac.uk.

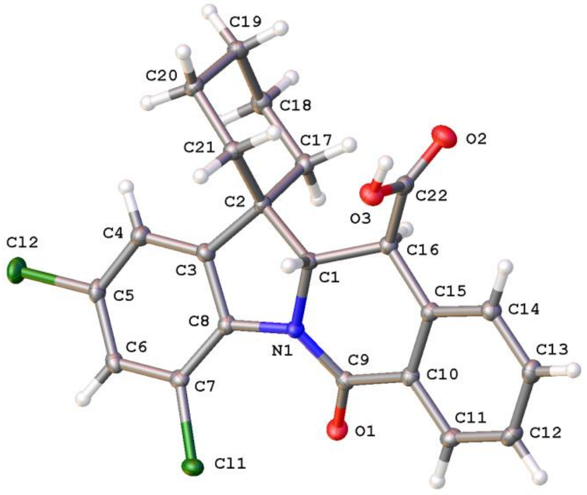

***anti*-10e**

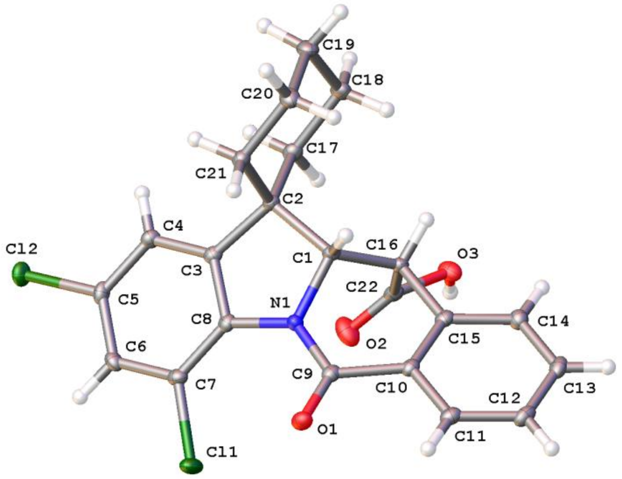

***syn*-10e**

Table S3 Crystal data and structure refinement for *anti*-10e		Table S4 Crystal data and structure refinement for *syn*-10e	
Empirical formula	$C_{22}H_{19}Cl_2NO_3$	Empirical formula	$C_{22}H_{19}Cl_2NO_3$
Formula weight	416.28	Formula weight	416.28
Temperature/K	100(2)	Temperature/K	100(2)
Crystal system	monoclinic	Crystal system	orthorhombic
Space group	$P2_1/c$	Space group	$P2_12_12_1$
a/Å	15.7357(3)	a/Å	9.8687(3)
b/Å	6.94798(11)	b/Å	12.2841(4)
c/Å	17.1351(3)	c/Å	15.4822(5)
α/°	90	α/°	90
β/°	101.9535(17)	β/°	90
γ/°	90	γ/°	90
Volume/Å^3	1832.78(5)	Volume/Å^3	1876.88(11)
Z	4	Z	4
ρ_{calc}g/cm^3	1.509	ρ_{calc}g/cm^3	1.473
μ/mm^{-1}	0.379	μ/mm^{-1}	0.370
F(000)	864.0	F(000)	864.0
Crystal size/mm^3	0.25 × 0.25 × 0.2	Crystal size/mm^3	0.25 × 0.25 × 0.2
Radiation	MoKα (λ = 0.71073)	Radiation	MoKα (λ = 0.71073)
2Θ range for data	5.996 to 54.99	2Θ range for data	5.914 to 54.99

collection/°		collection/°	
Index ranges	$-20 \le h \le 20$, $-9 \le k \le 9$, $-22 \le l \le 22$	Index ranges	$-12 \le h \le 11$, $-15 \le k \le 15$, $-20 \le l \le 20$
Reflections collected	32039	Reflections collected	18673
Independent reflections	4193 [R_{int} = 0.0240, R_{sigma} = 0.0133]	Independent reflections	4312 [R_{int} = 0.0274, R_{sigma} = 0.0240]
Data/restraints/parameters	4193/0/254	Data/restraints/parameters	4312/0/254
Goodness-of-fit on F^2	1.046	Goodness-of-fit on F^2	1.047
Final R indexes [I>=2σ (I)]	R_1 = 0.0340, wR_2 = 0.0841	Final R indexes [I>=2σ (I)]	R_1 = 0.0265, wR_2 = 0.0647
Final R indexes [all data]	R_1 = 0.0372, wR_2 = 0.0867	Final R indexes [all data]	R_1 = 0.0288, wR_2 = 0.0664
Largest diff. peak/hole / e Å^{-3}	0.48/-0.22	Largest diff. peak/hole / e Å^{-3}	0.32/-0.19
**CCDC**	**1503097**	**CCDC**	**1503105**

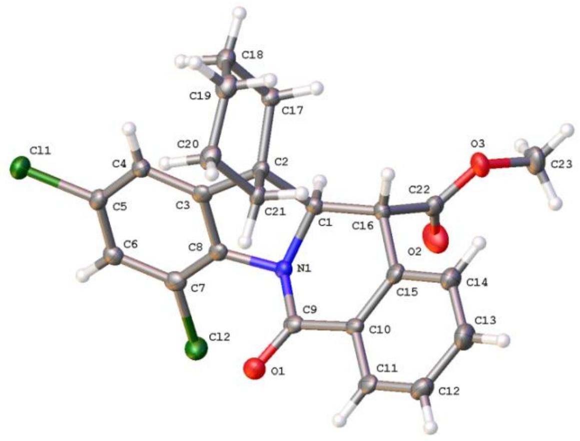

***anti*-10e'**

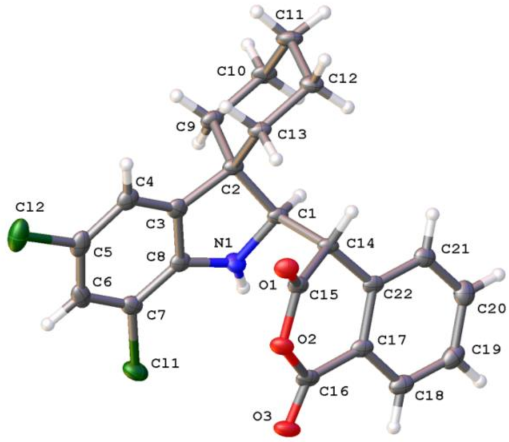

13e

**Table S5 Crystal data and structure refinement for *anti*-10e'**		**Table S6 Crystal data and structure refinement for 13e**	
Empirical formula	$C_{23}H_{21}Cl_2NO_3$	Empirical formula	$C_{22}H_{19}Cl_2NO_3$
Formula weight	430.31	Formula weight	416.28
Temperature/K	100(2)	Temperature/K	100(2)
Crystal system	trigonal	Crystal system	monoclinic
Space group	R3	Space group	$P2_1/c$
a/Å	20.4043(3)	a/Å	16.3985(8)
b/Å	20.4043(3)	b/Å	11.1961(6)
c/Å	13.6622(2)	c/Å	10.3370(5)
α/°	90	α/°	90
β/°	90	β/°	96.103(5)
γ/°	120	γ/°	90
Volume/Å^3	4926.02(15)	Volume/Å^3	1887.11(16)
Z	9	Z	4
ρ_{calc}g/cm^3	1.305	ρ_{calc}g/cm^3	1.465
μ/mm^{-1}	2.859	μ/mm^{-1}	3.297
F(000)	2016.0	F(000)	864.0
Crystal size/mm^3	0.3 × 0.2 × 0.2	Crystal size/mm^3	0.2 × 0.08 × 0.08
Radiation	CuKα (λ = 1.54184)	Radiation	CuKα (λ = 1.54184)
2Θ range for data collection/°	8.18 to 144.976	2Θ range for data collection/°	9.582 to 144.898
Index ranges	$-25 \le h \le 25$, $-22 \le k \le 25$,	Index ranges	$-20 \le h \le 20$, $-13 \le k$

	-16 ≤ l ≤ 16		≤ 9, -11 ≤ l ≤ 12
Reflections collected	16404	Reflections collected	7750
Independent reflections	4288 [R_{int} = 0.1334, R_{sigma} = 0.0628]	Independent reflections	3725 [R_{int} = 0.0521, R_{sigma} = 0.0614]
Data/restraints/parameters	4288/1/263	Data/restraints/parameters	3725/0/253
Goodness-of-fit on F^2	1.117	Goodness-of-fit on F^2	1.040
Final R indexes [I>=2σ (I)]	R_1 = 0.0450, wR_2 = 0.1109	Final R indexes [I>=2σ (I)]	R_1 = 0.0583, wR_2 = 0.1554
Final R indexes [all data]	R_1 = 0.1433, wR_2 = 0.1639	Final R indexes [all data]	R_1 = 0.0676, wR_2 = 0.1680
Largest diff. peak/hole / e Å^{-3}	0.84/-0.64	Largest diff. peak/hole / e Å^{-3}	0.66/-0.45
**CCDC**	**1503102**	**CCDC**	**1503093**

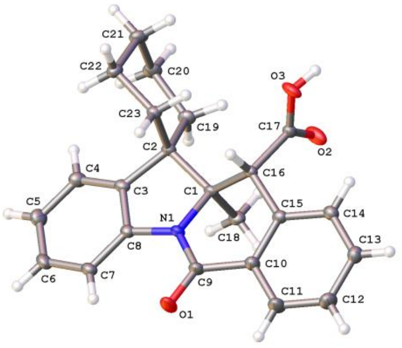

Crystal structure determination of *anti*-10n

Crystal Data for $C_{23}H_{23}NO_3$ (M =361.42 g/mol): monoclinic, space group C2/c (no. 15), a = 23.3057(6) Å, b = 12.7914(3) Å, c = 16.7508(4) Å, β = 133.0560(10)°, V = 3648.77(16) Å^3, Z = 8, T = 100(2) K, μ(MoKα) = 0.087 mm^{-1}, $Dcalc$ = 1.316 g/cm^3, 30249 reflections measured (6.15° ≤ 2Θ ≤ 54.996°), 4190 unique (R_{int} = 0.0207, R_{sigma} = 0.0109) which were used in all calculations. The final R_1 was 0.0366 (I > 2σ(I)) and wR_2 was 0.0998 (all data).

CCDC 1503103

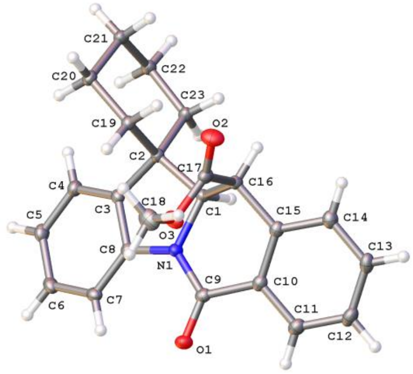

Crystal structure determination of *syn*-10a'

Crystal Data for $C_{23}H_{23}NO_3$ (M =361.42 g/mol): triclinic, space group P-1 (no. 2), a = 11.5749(2) Å, b = 12.2804(4) Å, c = 13.7928(4) Å, α = 68.362(3)°, β = 88.617(2)°, γ = 81.872(2)°, V = 1803.27(9) Å^3, Z = 4, T = 100(2) K, μ(CuKα) = 0.703 mm^{-1}, $Dcalc$ = 1.331 g/cm^3, 51248 reflections measured (6.898° ≤ 2Θ ≤ 152.6°), 7555 unique (R_{int} = 0.0531, R_{sigma} = 0.0235) which were used in all calculations. The final R_1 was 0.0374 (I > 2σ(I)) and wR_2 was 0.0977 (all data).

CCDC 1503100

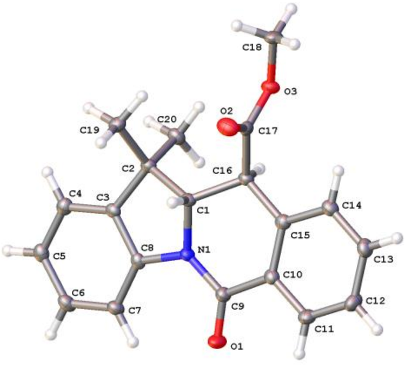

Crystal structure determination of *anti*-10g'

Crystal Data for $C_{20}H_{19}NO_3$ (M =321.36 g/mol): monoclinic, space group Cc (no. 9), a = 17.7696(7) Å, b = 13.3575(5) Å, c = 7.1549(3) Å, β = 109.372(3)°, V = 1602.12(11) Å^3, Z = 4, T = 100(2) K, μ(MoKα) = 0.090 mm^{-1}, $Dcalc$ = 1.332 g/cm^3, 7519 reflections measured (6.1° ≤ 2Θ ≤ 54.986°), 3473 unique (R_{int} = 0.0281, R_{sigma} = 0.0387) which were used in all calculations. The final R_1 was 0.0370 (I > 2σ(I)) and wR_2 was 0.0926 (all data).

CCDC 1503096

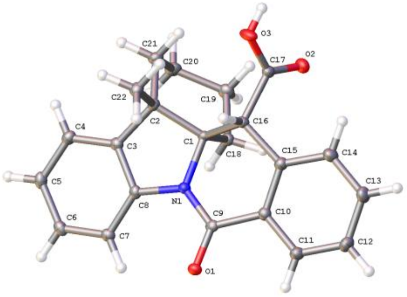

Crystal structure determination of *anti*-10l

Crystal Data for $C_{22}H_{21}NO_3$ (M =347.40 g/mol): monoclinic, space group C2/c (no. 15), a = 22.5031(6) Å, b = 13.5761(4) Å, c = 14.7923(4) Å, β = 129.571(2)°, V = 3483.49(18) Å^3, Z = 8, T = 100(2) K, μ(MoKα) = 0.088 mm^{-1}, $Dcalc$ = 1.325 g/cm^3, 29448 reflections measured (5.51° ≤ 2Θ ≤ 54.998°), 4001 unique (R_{int} = 0.0713, R_{sigma} = 0.0318) which were used in all calculations. The final R_1 was 0.0410 (I > 2σ(I)) and wR_2 was 0.1107 (all data).

CCDC 1503106

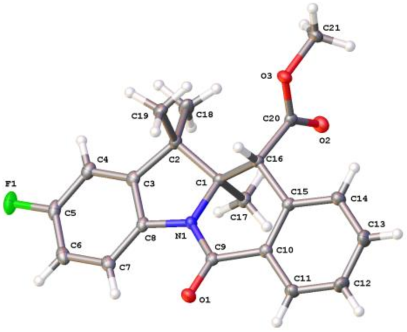

Crystal structure determination of *anti*-10i

Crystal Data for $C_{21}H_{20}FNO_3$ (M =353.38 g/mol): monoclinic, space group C2/c (no. 15), a = 26.5832(15) Å, b = 6.7549(2) Å, c = 21.1207(8) Å, β = 115.042(3)°, V = 3436.1(3) Å^3, Z = 8, T = 100(2) K, μ(MoKα) = 0.098 mm^{-1}, $Dcalc$ = 1.366 g/cm^3, 15007 reflections measured (6.264° ≤ 2Θ ≤ 54.994°), 3943 unique (R_{int} = 0.0255, R_{sigma} = 0.0244) which were used in all calculations. The final R_1 was 0.0371 (I > 2σ(I)) and wR_2 was 0.0945 (all data).

CCDC 1503104

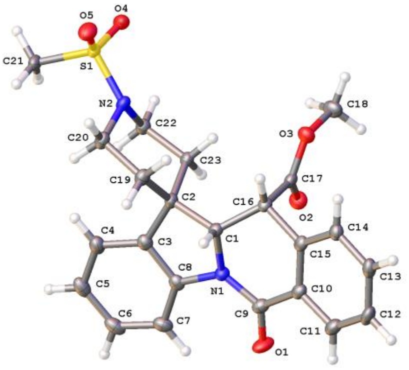

Crystal structure determination of *syn*-10f

Crystal Data for $C_{23}H_{24}N_2O_5S$ (M =440.50 g/mol): monoclinic, space group P2$_1$/c (no. 14), a = 11.2258(2) Å, b = 9.78991(18) Å, c = 19.3208(3) Å, β = 103.8379(19)°, V = 2061.71(7) Å^3, Z = 4, T = 100(2) K, μ(CuKα) = 1.730 mm^{-1}, $Dcalc$ = 1.419 g/cm^3, 14079 reflections measured (8.112° ≤ 2Θ ≤ 139.968°), 3904 unique (R_{int} = 0.0260, R_{sigma} = 0.0217) which were used in all calculations. The final R_1 was 0.0411 (I > 2σ(I)) and wR_2 was 0.1052 (all data).

CCDC 1503101

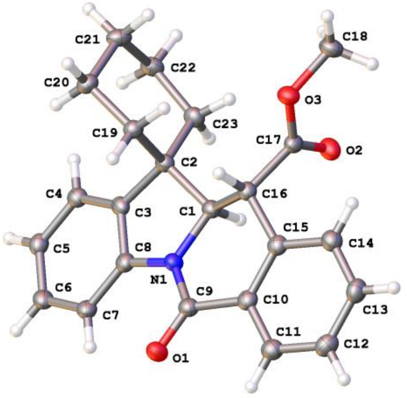

Crystal structure determination of *anti*-10a'

Crystal Data for $C_{23}H_{23}NO_3$ (M =361.42 g/mol): monoclinic, space group Cc (no. 9), a = 19.3213(7) Å, b = 13.2486(4) Å, c = 7.3482(2) Å, β = 109.212(3)°, V = 1776.24(10) Å^3, Z = 4, T = 100(2) K, μ(CuKα) = 0.713 mm^{-1}, $Dcalc$ = 1.352 g/cm^3, 12942 reflections measured (8.248° ≤ 2Θ ≤ 144.844°), 2996 unique (R_{int} = 0.0456, R_{sigma} = 0.0265) which were used in all calculations. The final R_1 was 0.0353 (I > 2σ(I)) and wR_2 was 0.0945 (all data).

CCDC 1503098

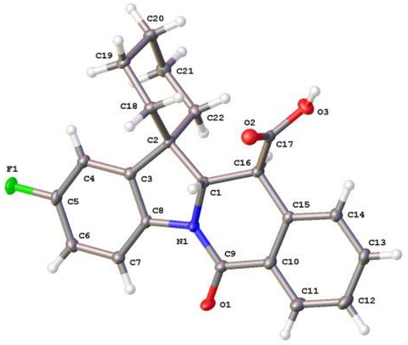

Crystal structure determination of *anti*-10c

Crystal Data for $C_{22}H_{20}FNO_3$ (M =365.39 g/mol): monoclinic, space group C2/c (no. 15), a = 22.2110(8) Å, b = 13.4818(4) Å, c = 15.1581(6) Å, β = 131.494(2)°, V = 3399.8(2) Å^3, Z = 8, T = 100(2) K, μ(MoKα) = 0.102 mm^{-1}, *Dcalc* = 1.428 g/cm^3, 15635 reflections measured (6.044° ≤ 2Θ ≤ 54.996°), 3905 unique (R_{int} = 0.0254, R_{sigma} = 0.0218) which were used in all calculations. The final R_1 was 0.0443 (I > 2σ(I)) and wR_2 was 0.1214 (all data).

CCDC 1503094

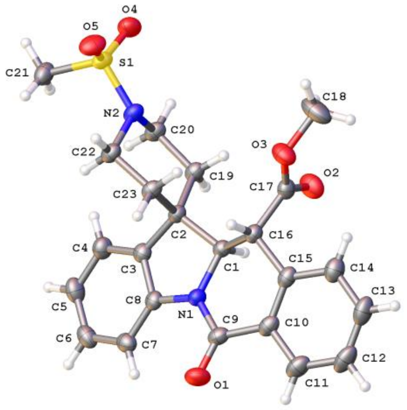

Crystal structure determination of *anti*-10f

Crystal Data for $C_{23}H_{24}N_2O_5S$ (M =440.50 g/mol): monoclinic, space group P2$_1$/c (no. 14), a = 14.5219(6) Å, b = 14.0079(4) Å, c = 10.9021(4) Å, β = 109.892(4)°, V = 2085.39(13) Å^3, Z = 4, T = 200(2) K, μ(MoKα) = 0.194 mm^{-1}, *Dcalc* = 1.403 g/cm^3, 26903 reflections measured (5.966° ≤ 2Θ ≤ 54.998°), 4782 unique (R_{int} = 0.0229, R_{sigma} = 0.0173) which were used in all calculations. The final R_1 was 0.0370 (I > 2σ(I)) and wR_2 was 0.0989 (all data).

CCDC 1503099

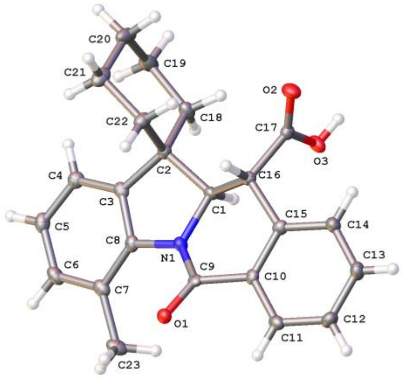

Crystal structure determination of *anti*-10d

Crystal Data for $C_{23}H_{23}NO_3$ (M =361.42 g/mol): orthorhombic, space group P2$_1$2$_1$2$_1$ (no. 19), a = 7.2945(3) Å, b = 14.2074(6) Å, c = 17.4589(7) Å, V = 1809.36(13) Å^3, Z = 4, T = 100(2) K, μ(MoKα) = 0.088 mm^{-1}, *Dcalc* = 1.327 g/cm^3, 14046 reflections measured (5.478° ≤ 2Θ ≤ 54.994°), 4173 unique (R_{int} = 0.0350, R_{sigma} = 0.0441) which were used in all calculations. The final R_1 was 0.0399 (I > 2σ(I)) and wR_2 was 0.0869 (all data).

CCDC 1503095

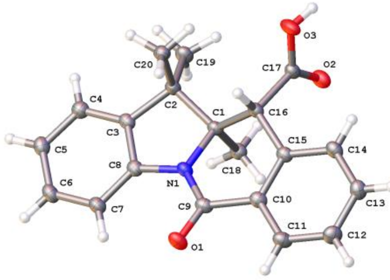

Crystal structure determination of *anti*-10h

Crystal Data for $C_{20}H_{19}NO_3$ (M =321.36 g/mol): orthorhombic, space group Pbca (no. 61), a = 14.9929(3) Å, b = 12.2708(3) Å, c = 16.6823(4) Å, V = 3069.13(12) Å^3, Z = 8, T = 100(2) K, μ(CuKα) = 0.755 mm^{-1}, *Dcalc* = 1.391 g/cm^3, 21935 reflections measured (10.606° ≤ 2Θ ≤ 144.948°), 2942 unique (R_{int} = 0.0610, R_{sigma} = 0.0353) which were used in all calculations. The final R_1 was 0.0507 (I > 2σ(I)) and wR_2 was 0.1481 (all data).

CCDC 1470389

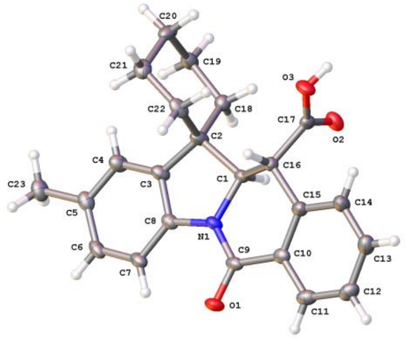

Crystal structure determination of *anti*-10b

Crystal Data for $C_{23}H_{23}NO_3$ (M =361.42 g/mol): monoclinic, space group P2$_1$/n (no. 14), a = 9.2717(2) Å, b = 11.18826(19) Å, c = 17.5072(4) Å, β = 105.013(2)°, V = 1754.11(7) Å^3, Z = 4, T = 100(2) K, μ(CuKα) = 0.722 mm^{-1}, $Dcalc$ = 1.369 g/cm^3, 9756 reflections measured (9.478° ≤ 2Θ ≤ 144.978°), 3397 unique (R_{int} = 0.0237, R_{sigma} = 0.0205) which were used in all calculations. The final R_1 was 0.0516 (I > 2σ(I)) and wR_2 was 0.1243 (all data).

CCDC 1470399

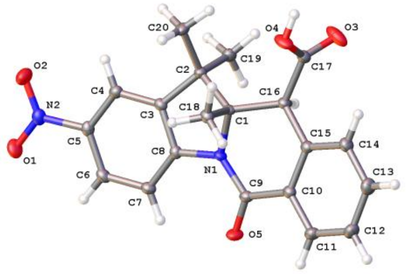

Crystal structure determination of *anti*-10j

Crystal Data for $C_{20}H_{18}N_2O_5$ (M =366.36 g/mol): monoclinic, space group P2$_1$/c (no. 14), a = 8.8454(3) Å, b = 14.1900(4) Å, c = 13.5995(4) Å, β = 104.646(3)°, V = 1651.50(9) Å^3, Z = 4, T = 100(2) K, μ(MoKα) = 0.107 mm^{-1}, $Dcalc$ = 1.473 g/cm^3, 14296 reflections measured (5.558° ≤ 2Θ ≤ 54.998°), 3807 unique (R_{int} = 0.0259, R_{sigma} = 0.0249) which were used in all calculations. The final R_1 was 0.0398 (I > 2σ(I)) and wR_2 was 0.1001 (all data).

CCDC 1461790

6. References:

1. Golubev, P.; Bakulina, O.; Dar'in, D.; Krasavin, M. Eur. J. Org. Chem., 2016, 3969.
2. Ji, C.; Ma, L.; Yin, M.; Yang, W.; Pan, K. *Chem. Asian J.*, **2016**, *11*, 2316.
3. Szaloki, G.; Sanguinet, L. *J. Org. Chem.*, **2015**, *80*, 3949.
4. Balmond, E. I.; Tautgeg, B. K.; Faulkner, A. L.; Or, V. W.; Hodur, B. M.; Shaw, J. T.; Louie, A. Y. *J. Org. Chem.*, **2016**, *81*, 8744.
5. Schultz-Senft, M.; Gates, P. J.; Soennichsen, F. J.; Stanbitz, A. *Dyes Pigm.*, **2017**, *136*, 292.
6. Prostoba, Y.; Coelho, P. J.; Pina, J.; Seixas de Melo, J. *J. Photochem. Photobiol.*, **2010**, *216*, 59.
7. Gore, S.; Baskaran, S.; Koenig, B. *Org. Lett.*, **2012**, *14*, 4568.
8. Li, H.; Luan, Z.-J.; Zheng, G.-W.; Xu, J.-H. *Adv. Synth. Catal.*, **2015**, *357*, 1692.
9. Deniz, E.; Battal, M.; Cusido, J.; Sortino, S.; Raymo, F. M. *Phys. Chem. Chem. Phys.*, **2012**, *14*, 10300.
10. Lin, A.; Yang, J.; Hashim, M. *Org. Lett.*, **2013**, *15*, 1950.
11. Kwan, E.E.; Shaw, G.H. *Eur. J. Org. Chem.* **2008**, 2671–2688.
12. Bifulco, G.; Dambruoso, P.; Gomez-Paloma, L.; Riccio, R. *Chem. Rev.*, **2007**, *107*, 3744
13. Palatinus, L. & Chapuis, G. (2007). J. Appl. Cryst., 40, 786-790; Palatinus, L. & van der Lee, A. (2008). J. Appl. Cryst. 41, 975-984; Palatinus, L., Prathapa, S. J. & van Smaalen, S. (2012). J. Appl. Cryst. 45, 575-580.
14. Sheldrick, G.M. (2008). Acta Cryst. A64, 112-122.
15. Sheldrick, G.M. (2015). Acta Cryst. C71, 3-8.
16. Dolomanov, O.V., Bourhis, L.J., Gildea, R.J, Howard, J.A.K. & Puschmann, H. (2009), J. Appl. Cryst. 42, 339-341.

17. Spek, A. L. PLATON, A Multipurpose Crystallographic Tool; Utrecht University: Utrecht, The Netherlands, 2005.

7. Copies of ^{1}H and ^{13}C NMR spectra of compounds 9 and 10

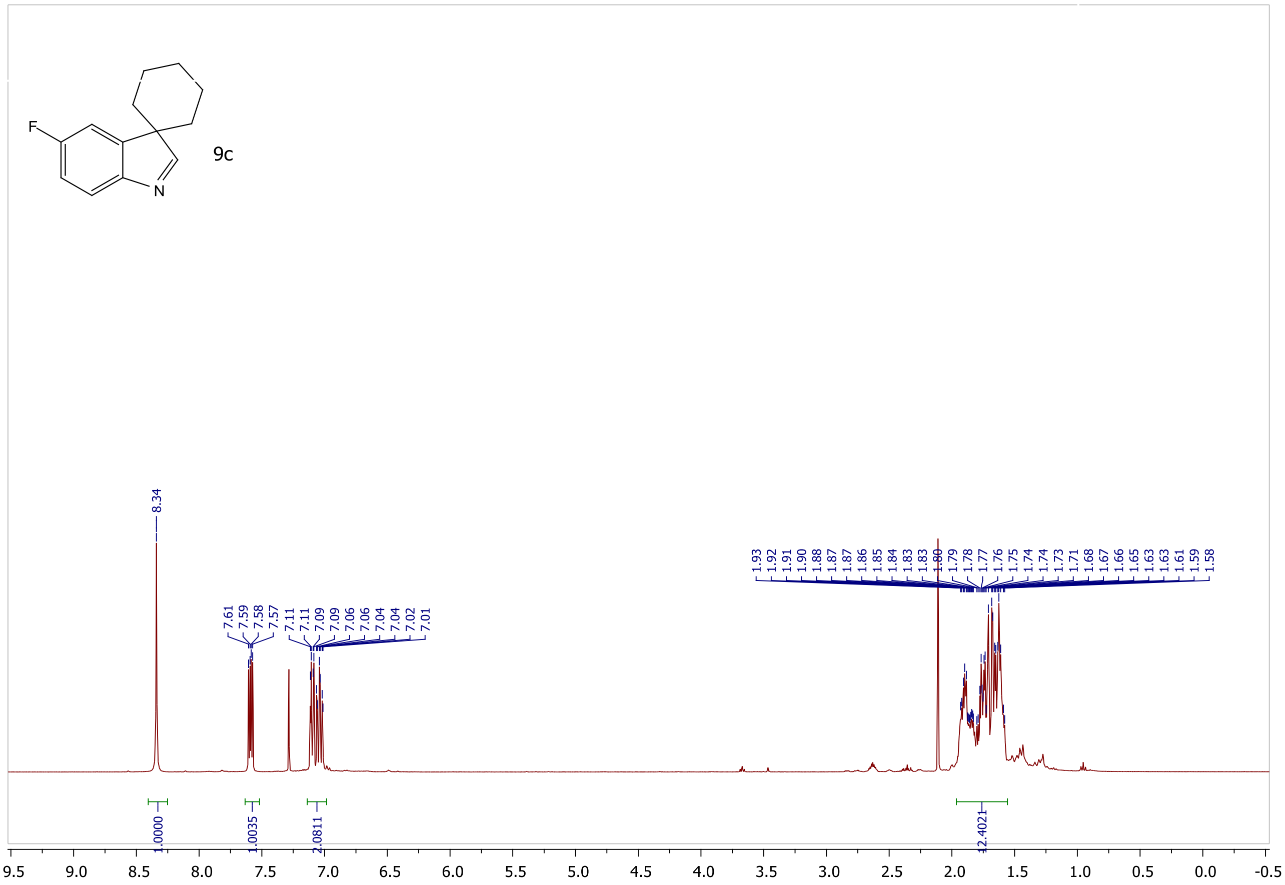

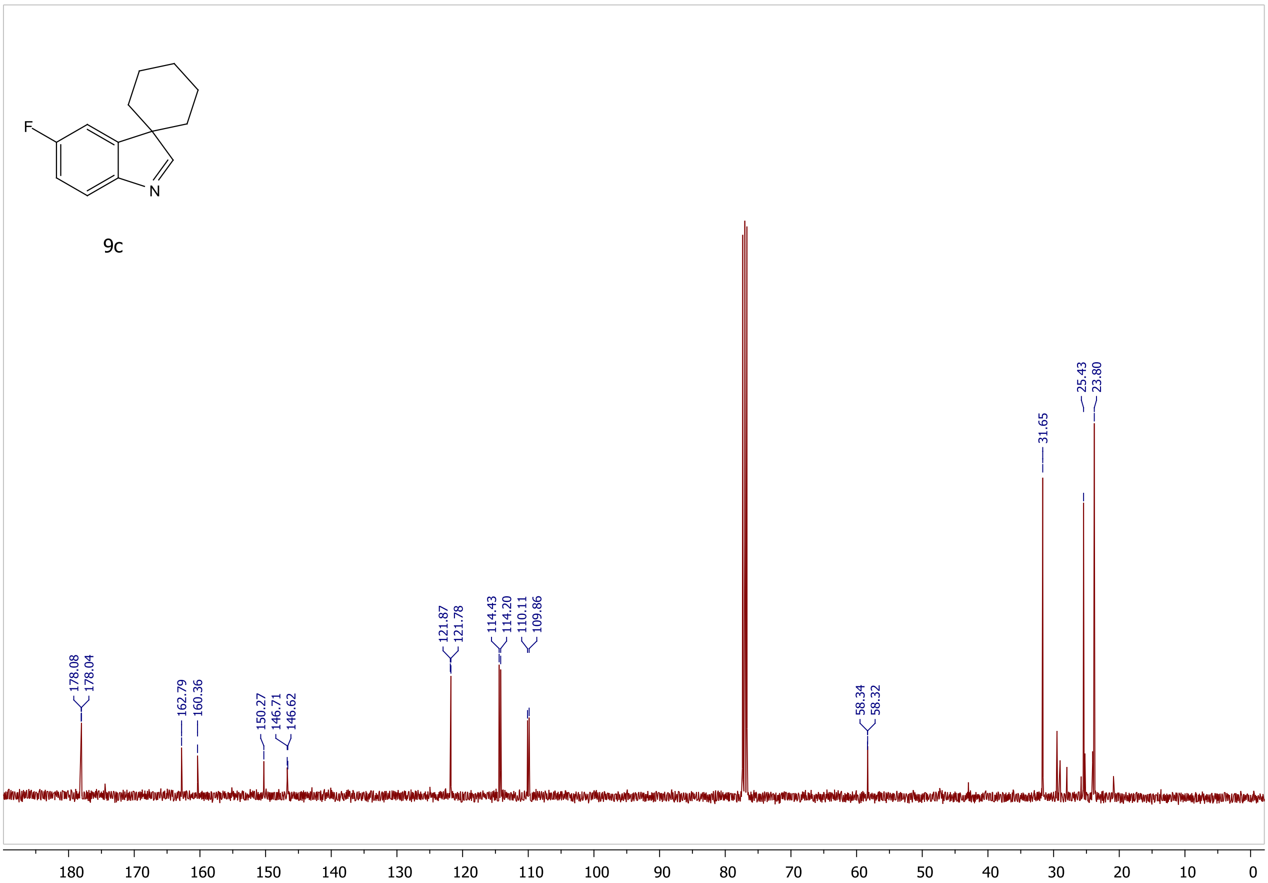
F
N
9c
178.08
178.04
162.79
160.36
150.27
146.71
146.62
121.87
121.78
114.43
114.20
110.11
109.86
58.34
58.32
31.65
25.43
23.80
180 170 160 150 140 130 120 110 100 90 80 70 60 50 40 30 20 10 0

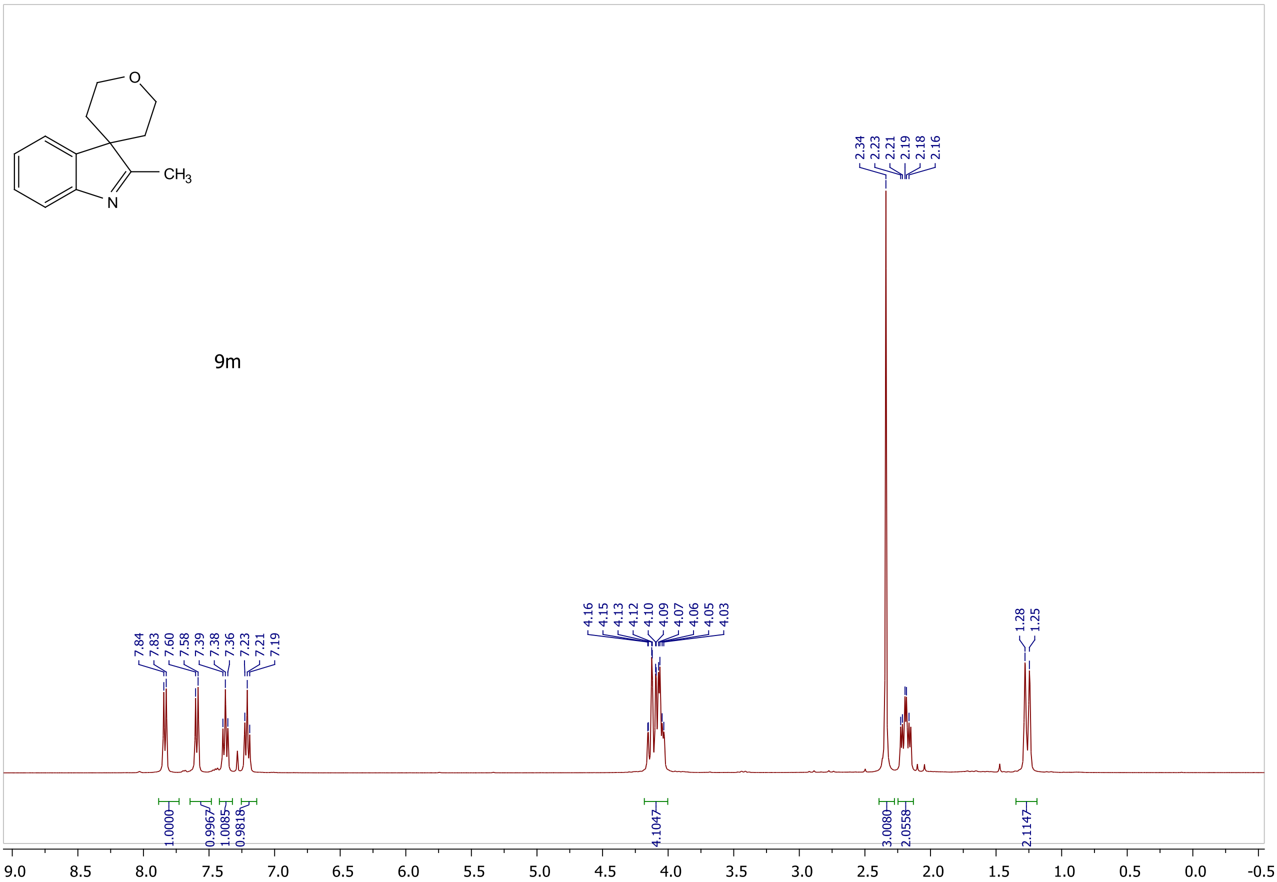
O
CH_3
N
9m
7.84
7.83
7.60
7.58
7.39
7.38
7.36
7.23
7.21
7.19
4.16
4.15
4.13
4.12
4.10
4.09
4.07
4.06
4.05
4.03
2.34
2.23
2.21
2.19
2.18
2.16
1.28
1.25
1.0000
0.9967
1.0085
0.9818
4.1047
3.0080
2.0558
2.1147
9.0 8.5 8.0 7.5 7.0 6.5 6.0 5.5 5.0 4.5 4.0 3.5 3.0 2.5 2.0 1.5 1.0 0.5 0.0 -0.5

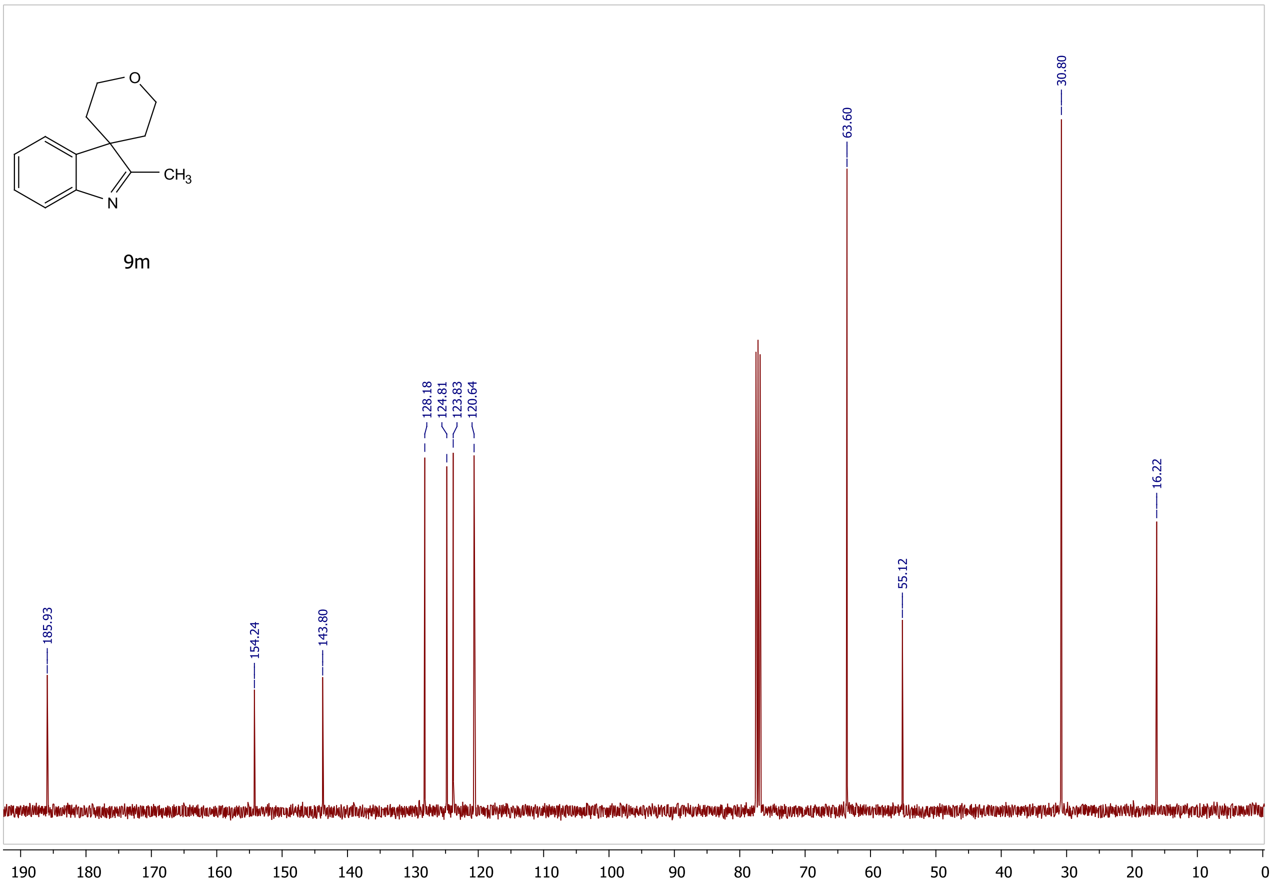

O
CH3
N
9m
185.93
154.24
143.80
128.18
124.81
123.83
120.64
63.60
55.12
30.80
16.22
190 180 170 160 150 140 130 120 110 100 90 80 70 60 50 40 30 20 10 0

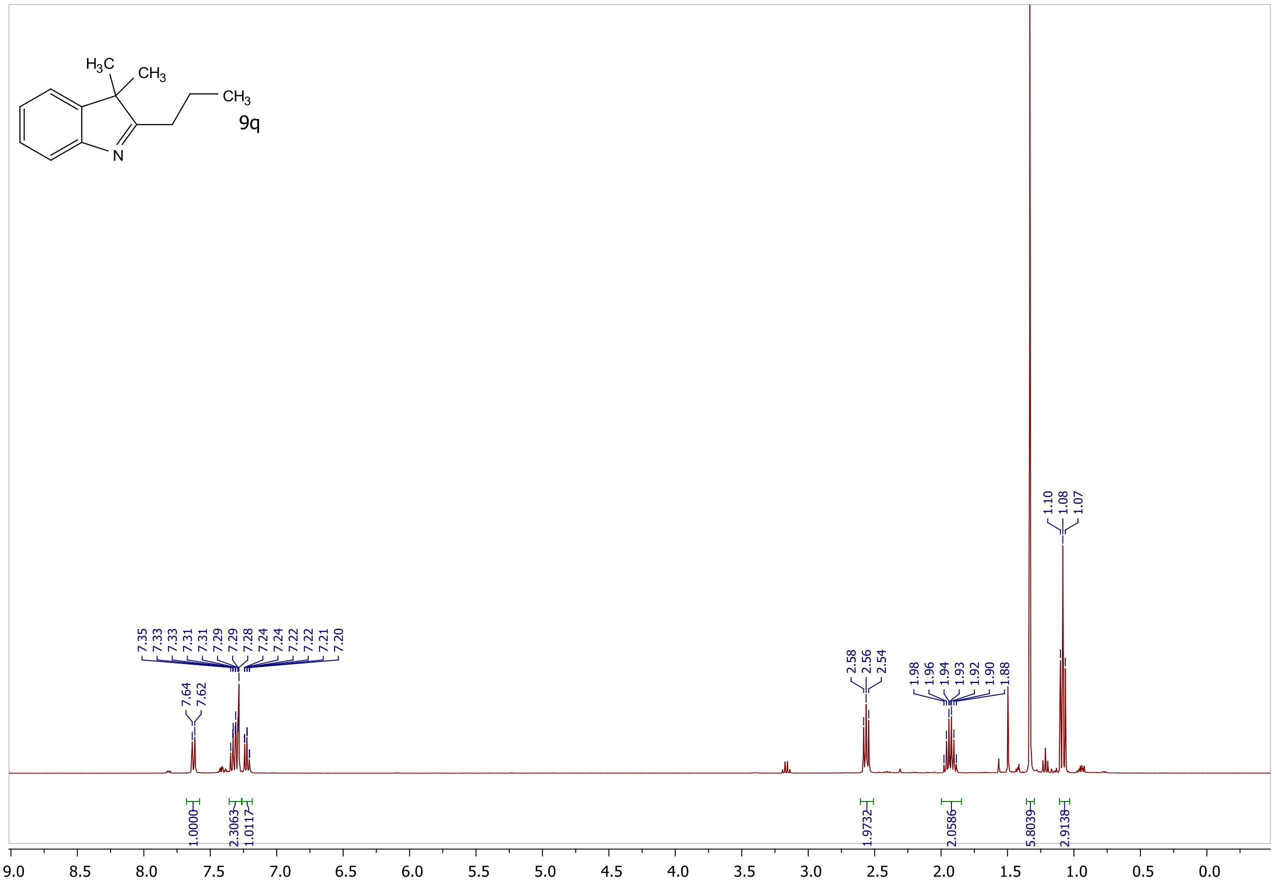

H3C
CH3
CH3
N
9q
7.35 7.33 7.33 7.31 7.31 7.29 7.29 7.28 7.24 7.24 7.22 7.22 7.21 7.20
7.64 7.62
2.58 2.56 2.54
1.98 1.96 1.94 1.93 1.92 1.90 1.88
1.10 1.08 1.07
1.0000
2.3063
1.0117
1.9732
2.0586
5.8039
2.9138
9.0 8.5 8.0 7.5 7.0 6.5 6.0 5.5 5.0 4.5 4.0 3.5 3.0 2.5 2.0 1.5 1.0 0.5 0.0

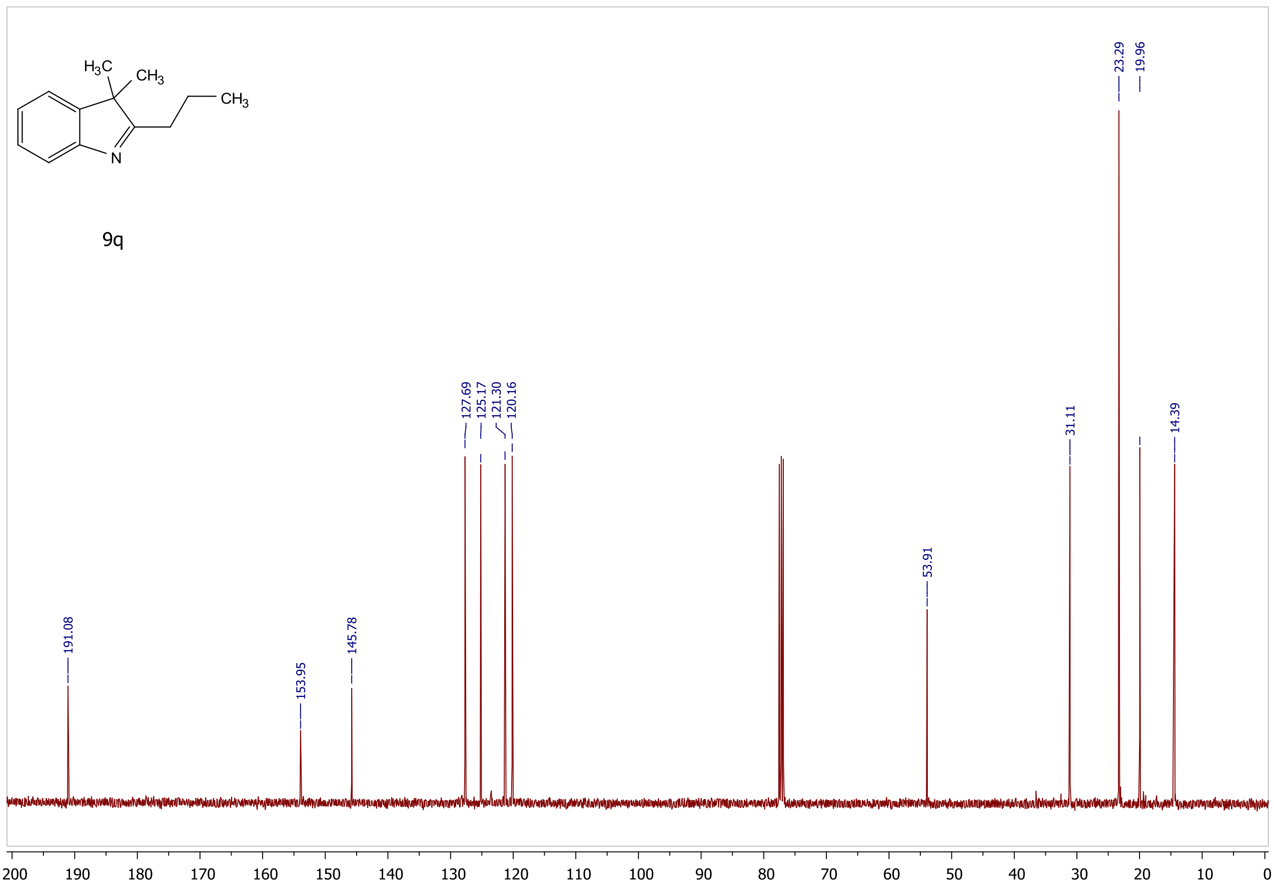

H3C
CH3
CH3
N
9q
191.08
153.95
145.78
127.69
125.17
121.30
120.16
53.91
31.11
23.29
19.96
14.39
200 190 180 170 160 150 140 130 120 110 100 90 80 70 60 50 40 30 20 10 0

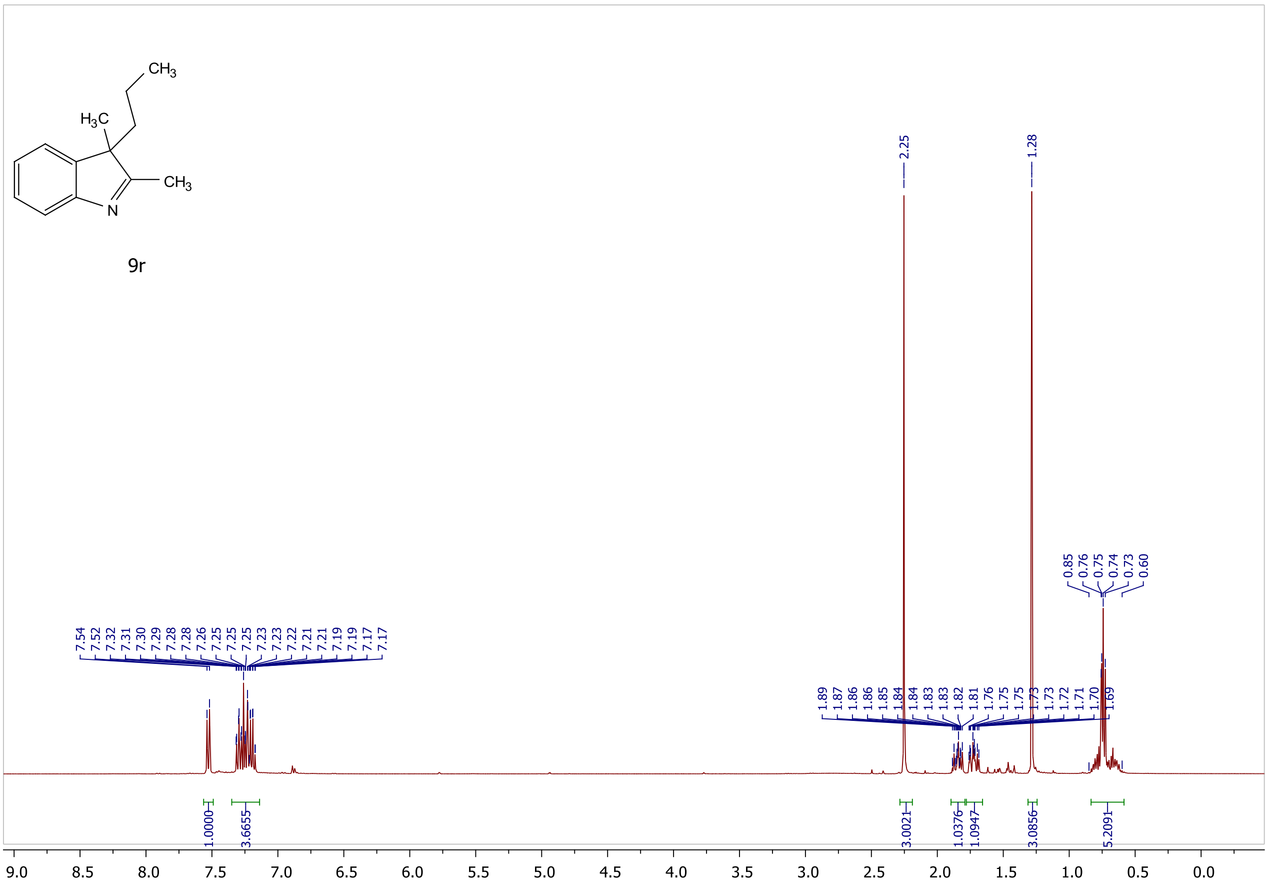

CH3
H3C
CH3
N
9r
7.54 7.52 7.32 7.31 7.30 7.29 7.28 7.28 7.26 7.25 7.25 7.25 7.23 7.23 7.22 7.21 7.21 7.19 7.19 7.17 7.17
2.25
1.28
1.89 1.87 1.86 1.86 1.85 1.84 1.84 1.83 1.83 1.82 1.81 1.76 1.75 1.75 1.73 1.73 1.72 1.71 1.70 1.69
0.85 0.76 0.75 0.74 0.73 0.60
1.0000 3.6655 3.0021 1.0376 1.0947 3.0856 5.2091
9.0 8.5 8.0 7.5 7.0 6.5 6.0 5.5 5.0 4.5 4.0 3.5 3.0 2.5 2.0 1.5 1.0 0.5 0.0

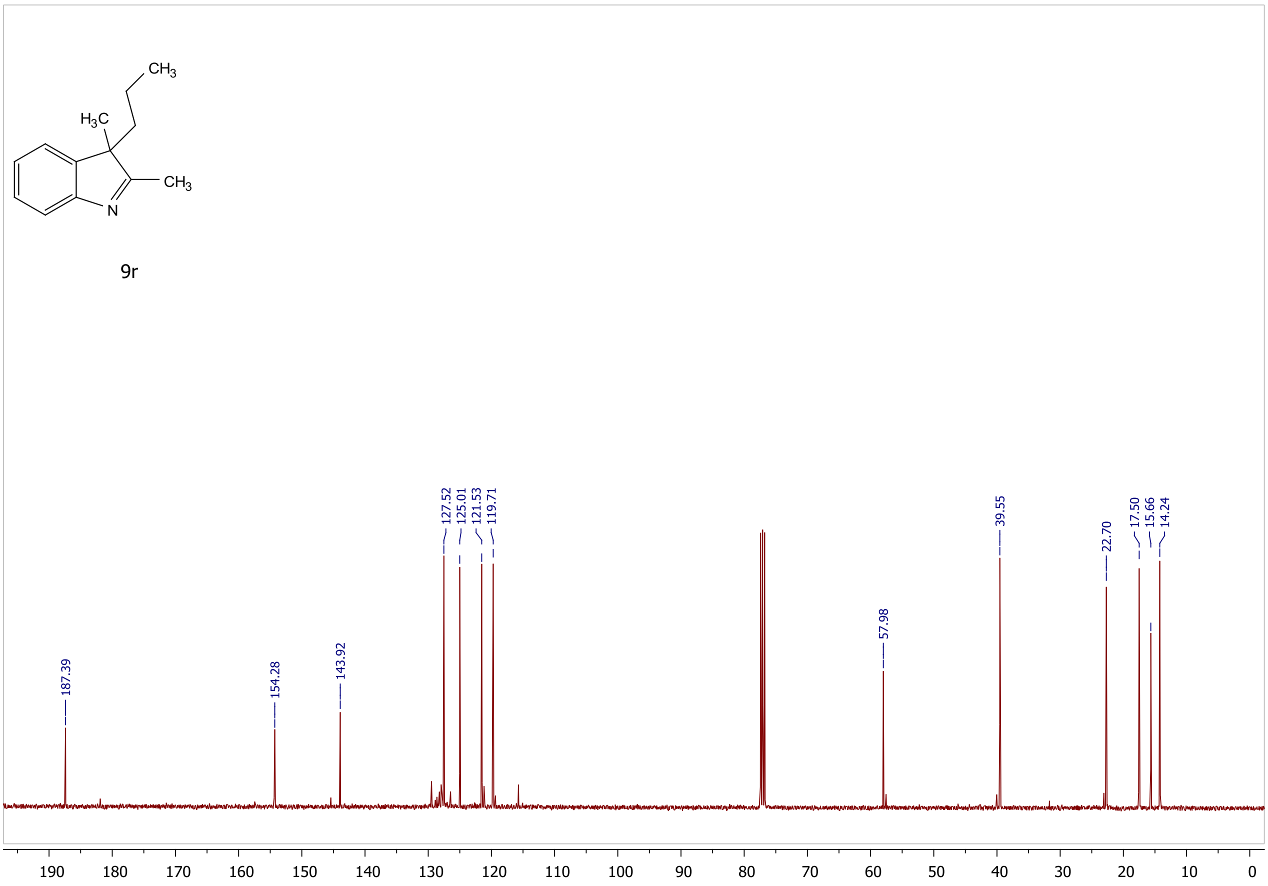

CH3
H3C
CH3
N
9r
187.39
154.28
143.92
127.52 125.01 121.53 119.71
57.98
39.55
22.70
17.50 15.66 14.24
190 180 170 160 150 140 130 120 110 100 90 80 70 60 50 40 30 20 10 0

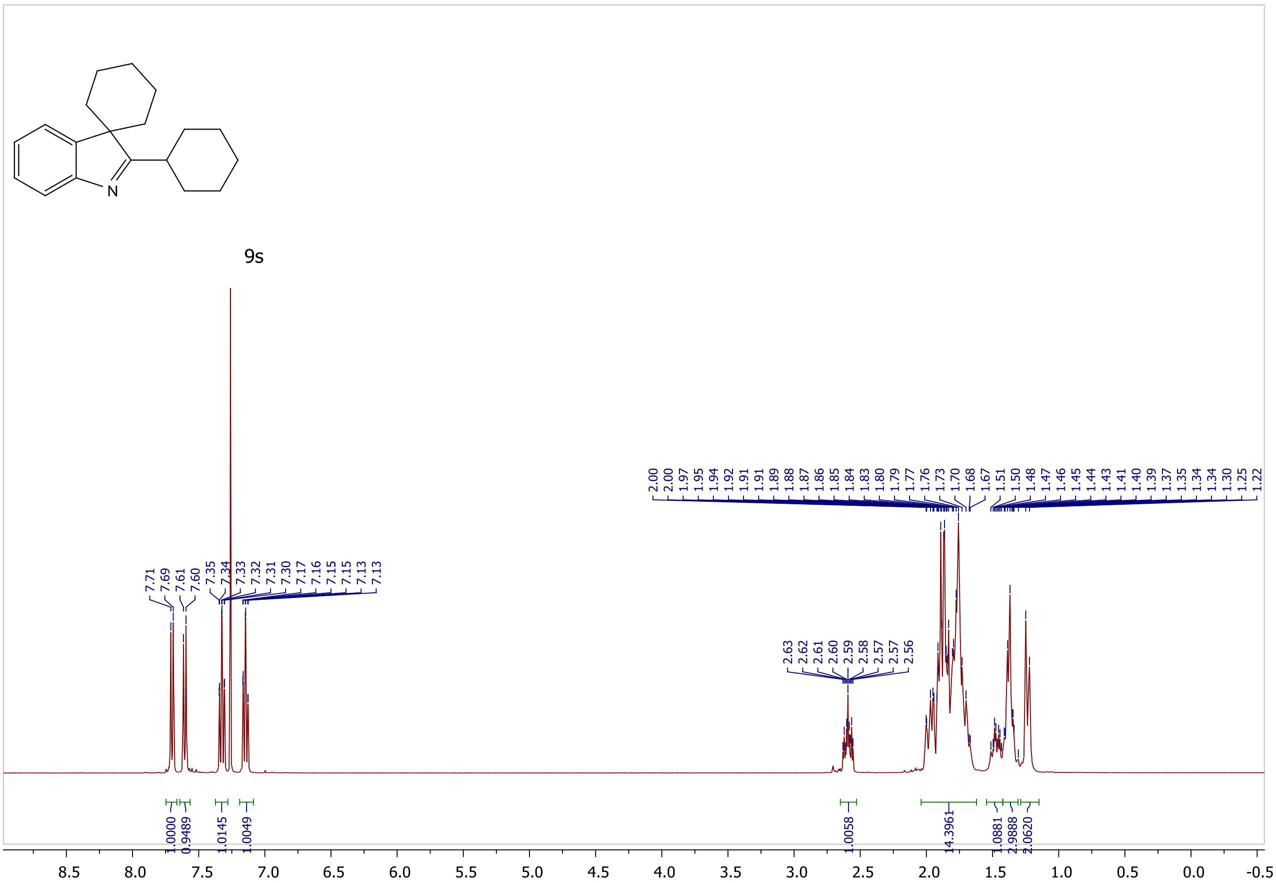
N
9s
7.71 7.69 7.61 7.60 7.35 7.34 7.33 7.32 7.31 7.30 7.17 7.16 7.15 7.15 7.13 7.13
2.63 2.62 2.61 2.60 2.59 2.58 2.57 2.57 2.56
2.00 2.00 1.97 1.95 1.94 1.92 1.91 1.91 1.89 1.88 1.87 1.86 1.85 1.84 1.83 1.80 1.79 1.77 1.76 1.73 1.70 1.68 1.67 1.51 1.50 1.48 1.47 1.46 1.45 1.44 1.43 1.41 1.40 1.39 1.37 1.35 1.34 1.34 1.30 1.25 1.22
1.0000 0.9489 1.0145 1.0049 1.0058 14.3961 1.0881 2.9888 2.0620
8.5 8.0 7.5 7.0 6.5 6.0 5.5 5.0 4.5 4.0 3.5 3.0 2.5 2.0 1.5 1.0 0.5 0.0 -0.5

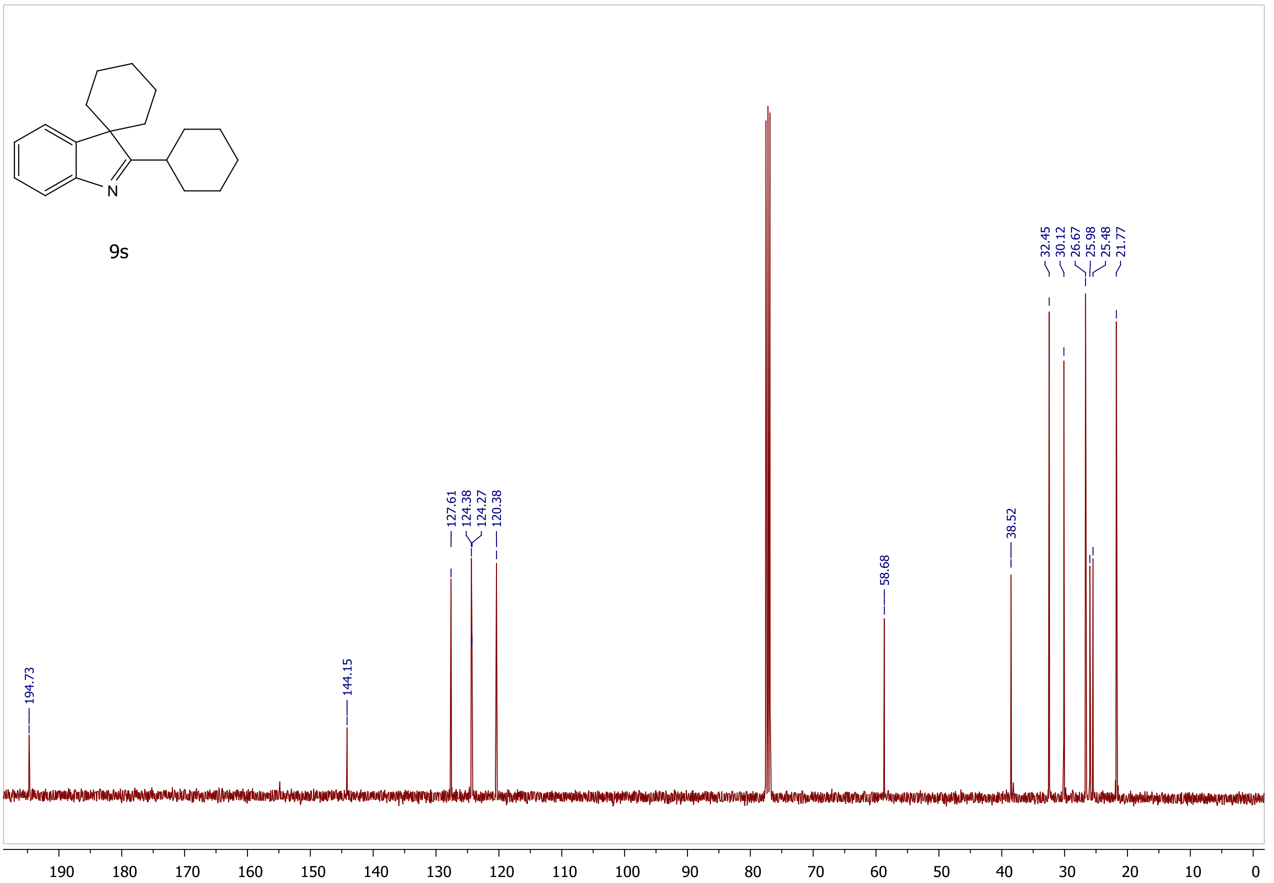
N
9s
194.73
144.15
127.61 124.38 124.27 120.38
58.68
38.52
32.45 30.12 26.67 25.98 25.48 21.77
190 180 170 160 150 140 130 120 110 100 90 80 70 60 50 40 30 20 10 0

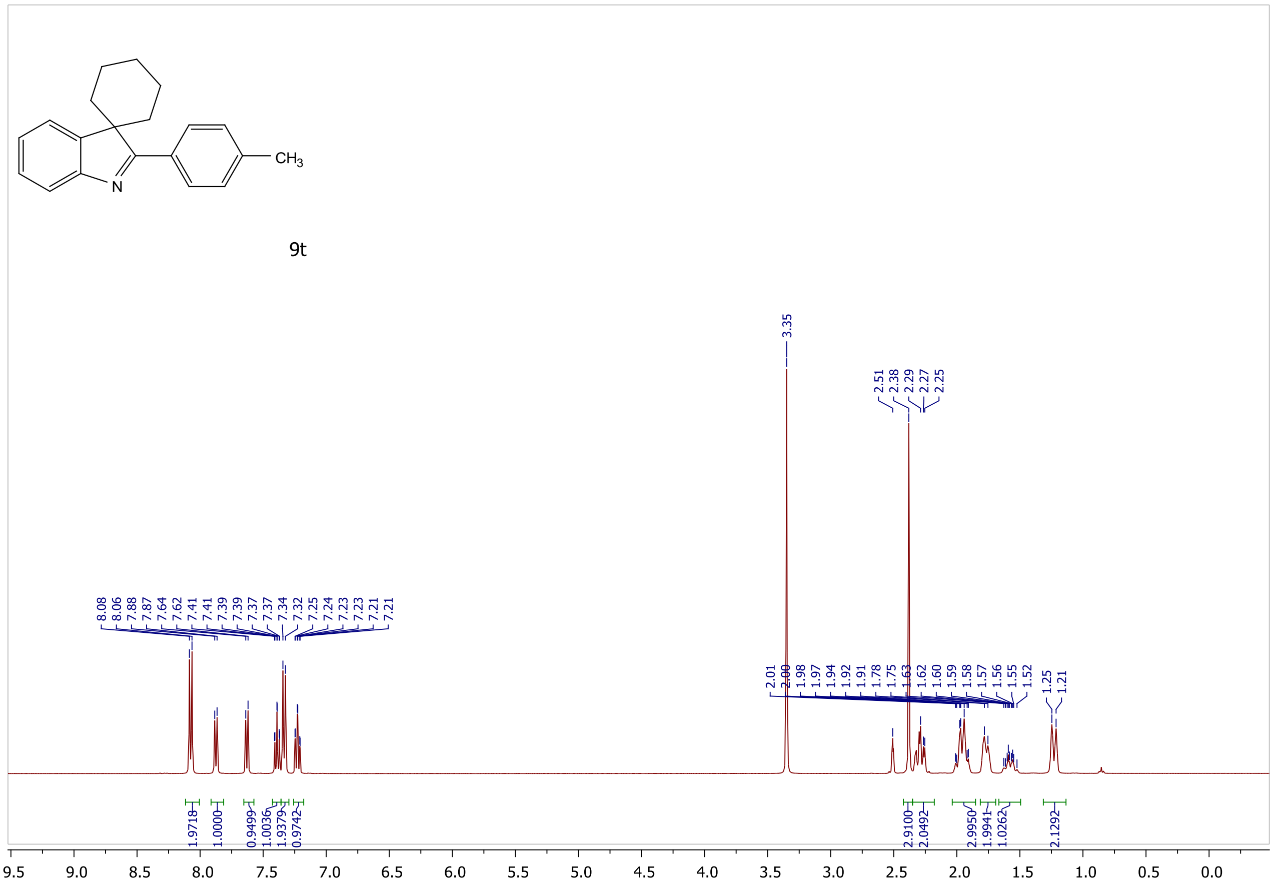

CH3
N
9t
3.35
2.51 2.38 2.29 2.27 2.25
8.08 8.06 7.88 7.87 7.64 7.62 7.41 7.41 7.39 7.39 7.37 7.34 7.32 7.25 7.24 7.23 7.23 7.21 7.21
2.01 2.00 1.98 1.97 1.94 1.92 1.91 1.78 1.75 1.63 1.62 1.60 1.59 1.58 1.57 1.56 1.55 1.52 1.25 1.21
1.9718 1.0000 0.9499 1.0036 1.9379 0.9742 2.9100 2.0492 2.9950 1.9941 1.0262 2.1292
9.5 9.0 8.5 8.0 7.5 7.0 6.5 6.0 5.5 5.0 4.5 4.0 3.5 3.0 2.5 2.0 1.5 1.0 0.5 0.0

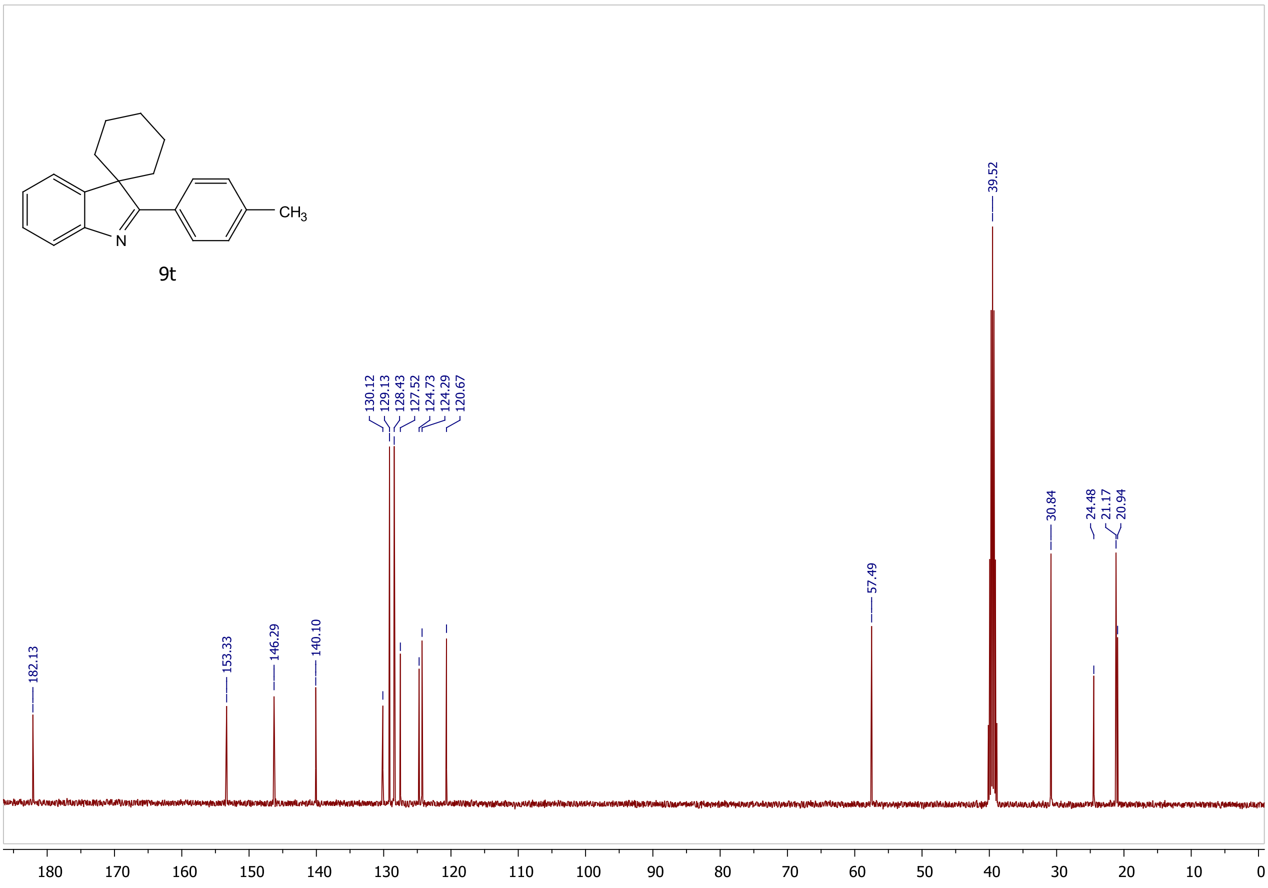

CH3
N
9t
39.52
130.12 129.13 128.43 127.52 124.73 124.29 120.67
30.84
24.48 21.17 20.94
57.49
182.13
153.33
146.29
140.10
180 170 160 150 140 130 120 110 100 90 80 70 60 50 40 30 20 10 0

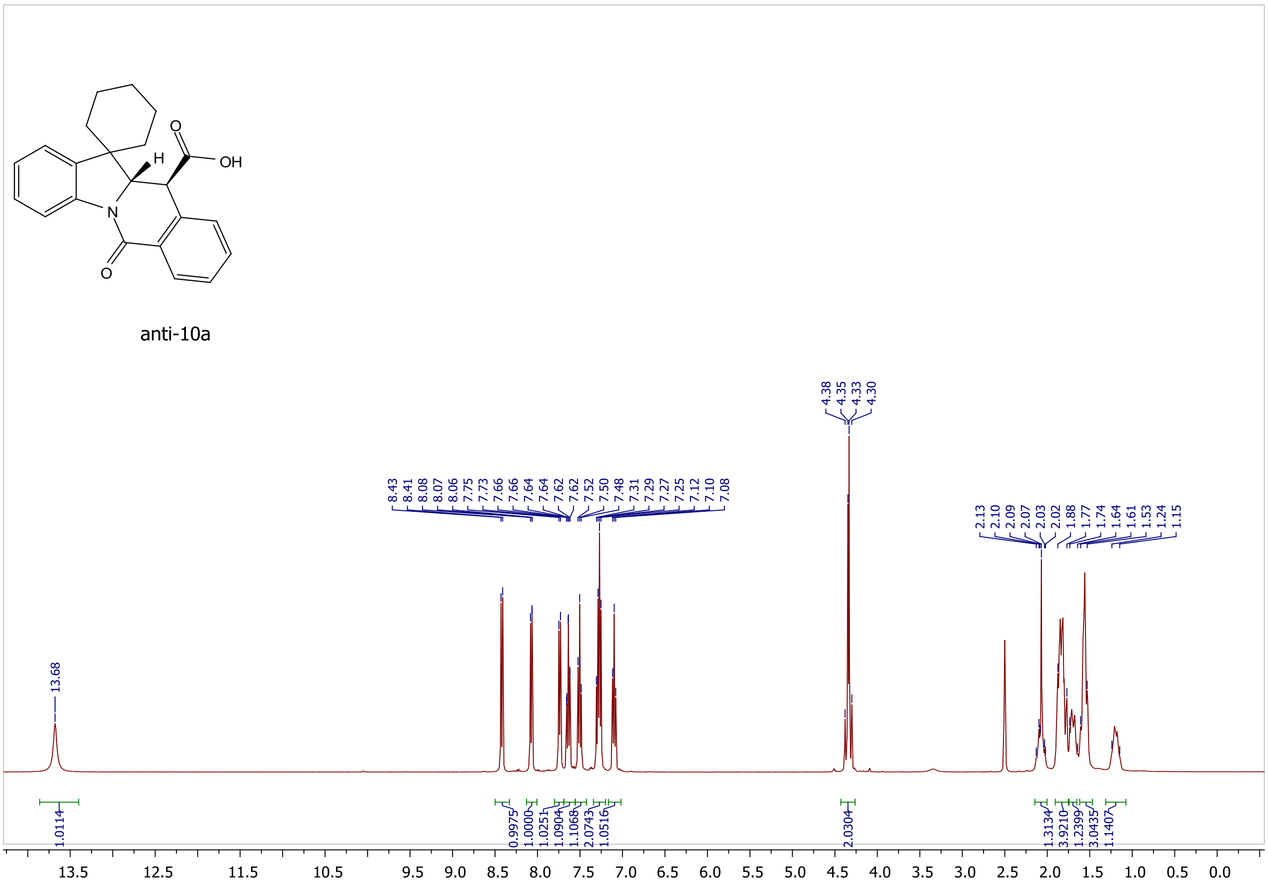
anti-10a
H
OH
O
N
O
13.68
8.43 8.41 8.08 8.07 8.06 7.75 7.73 7.66 7.66 7.64 7.64 7.62 7.62 7.52 7.50 7.48 7.31 7.29 7.27 7.25 7.12 7.10 7.08
4.38 4.35 4.33 4.30
2.13 2.10 2.09 2.07 2.03 2.02 1.88 1.77 1.74 1.64 1.61 1.53 1.24 1.15
1.0114
0.9975 1.0000 1.0251 1.0904 1.1068 2.0743 1.0516
2.0304
1.3134 3.9210 1.2399 3.0435 1.1407
13.5 12.5 11.5 10.5 9.5 9.0 8.5 8.0 7.5 7.0 6.5 6.0 5.5 5.0 4.5 4.0 3.5 3.0 2.5 2.0 1.5 1.0 0.5 0.0

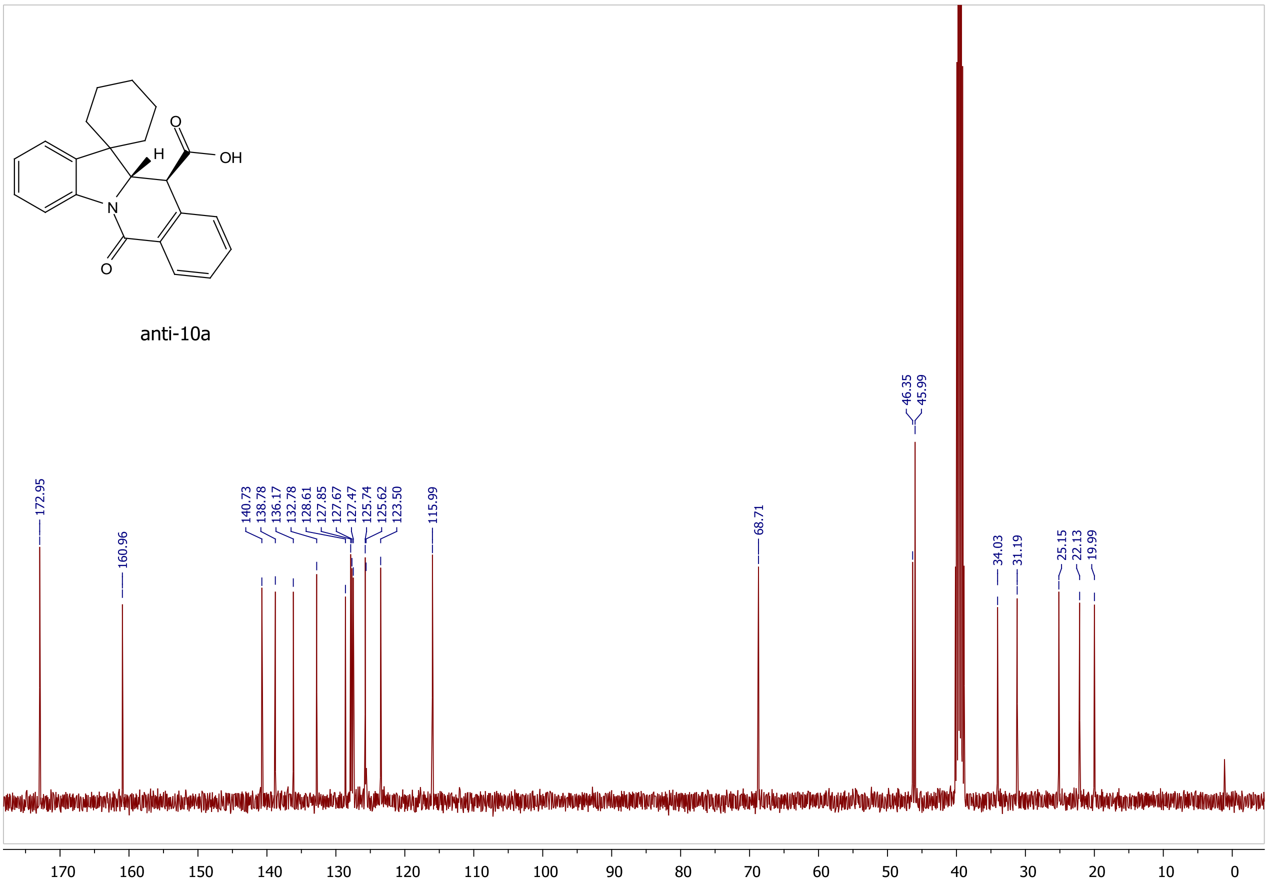
anti-10a
H
OH
O
N
O
172.95
160.96
140.73 138.78 136.17 132.78 128.61 127.85 127.67 127.47 125.74 125.62 123.50
115.99
68.71
46.35 45.99
34.03 31.19
25.15 22.13 19.99
170 160 150 140 130 120 110 100 90 80 70 60 50 40 30 20 10 0

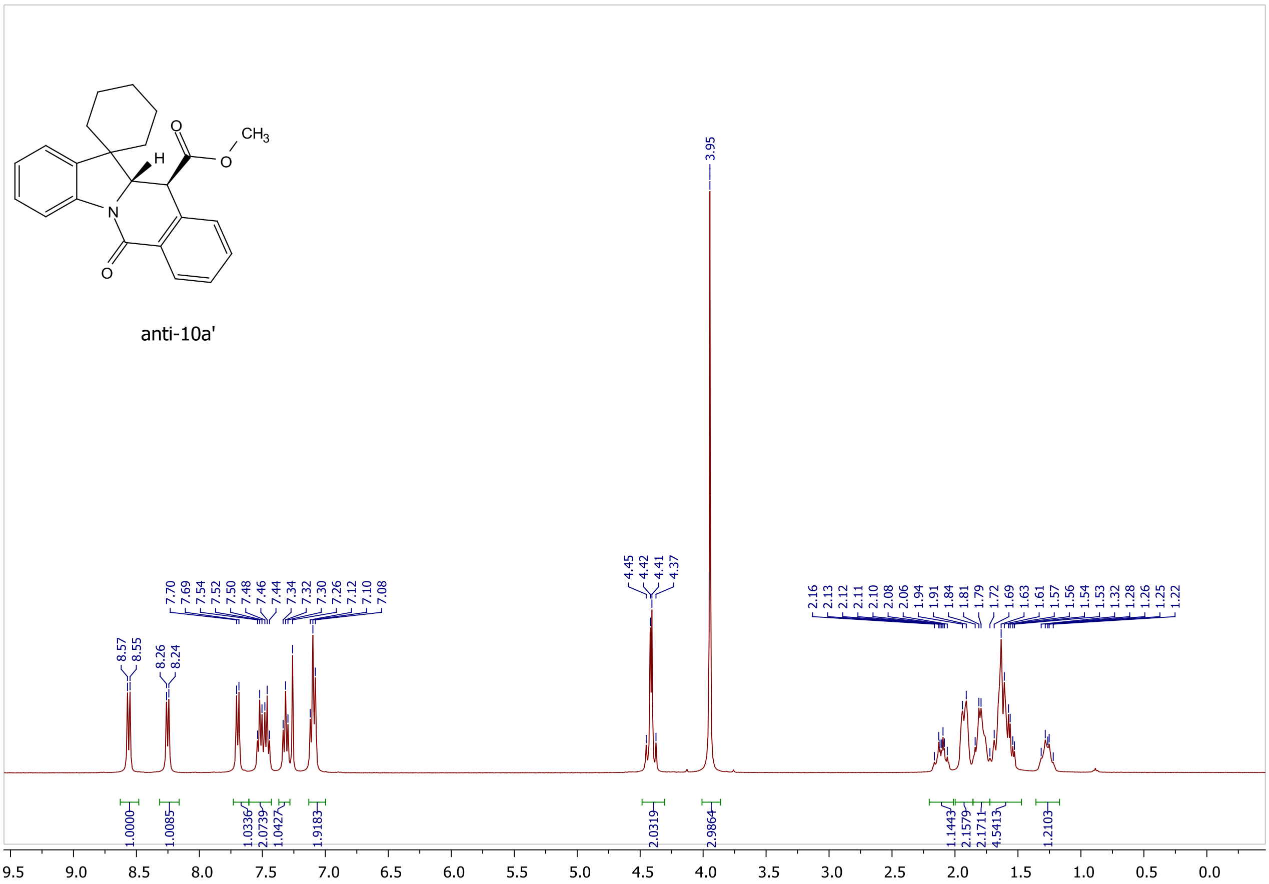
CH3
H
N
O
anti-10a'
8.57
8.55
8.26
8.24
7.70 7.69 7.54 7.52 7.50 7.48 7.46 7.44 7.34 7.32 7.30 7.26 7.12 7.10 7.08
4.45 4.42 4.41 4.37
3.95
2.16 2.13 2.12 2.11 2.10 2.08 2.06 1.94 1.91 1.84 1.81 1.79 1.72 1.69 1.63 1.61 1.57 1.56 1.54 1.53 1.32 1.28 1.26 1.25 1.22
1.0000 1.0085 1.0336 2.0739 1.0427 1.9183 2.0319 2.9864 1.1443 2.1579 2.1711 4.5413 1.2103
9.5 9.0 8.5 8.0 7.5 7.0 6.5 6.0 5.5 5.0 4.5 4.0 3.5 3.0 2.5 2.0 1.5 1.0 0.5 0.0

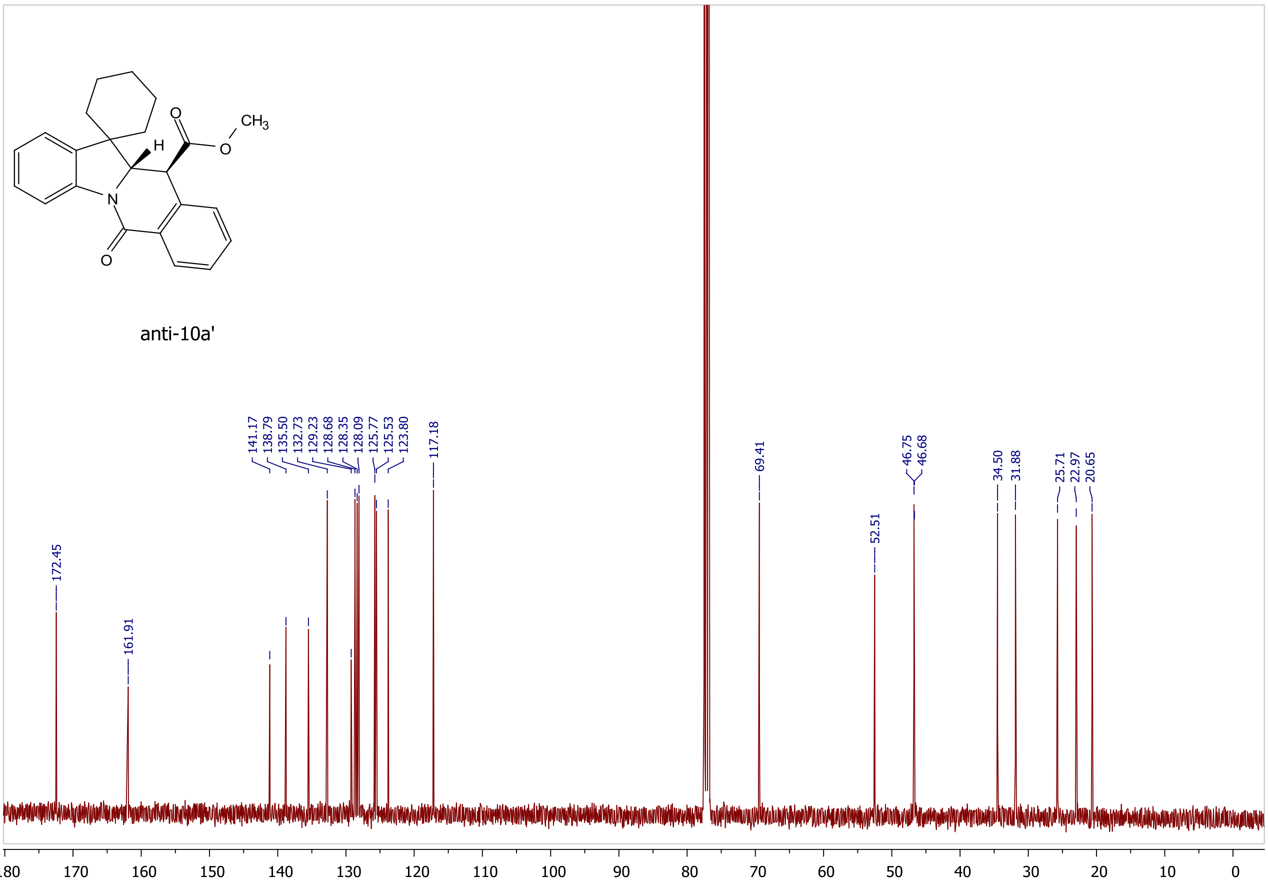
CH3
H
N
O
anti-10a'
172.45
161.91
141.17 138.79 135.50 132.73 129.23 128.68 128.35 128.09 125.77 125.53 123.80
117.18
69.41
52.51
46.75 46.68
34.50 31.88
25.71 22.97 20.65
180 170 160 150 140 130 120 110 100 90 80 70 60 50 40 30 20 10 0

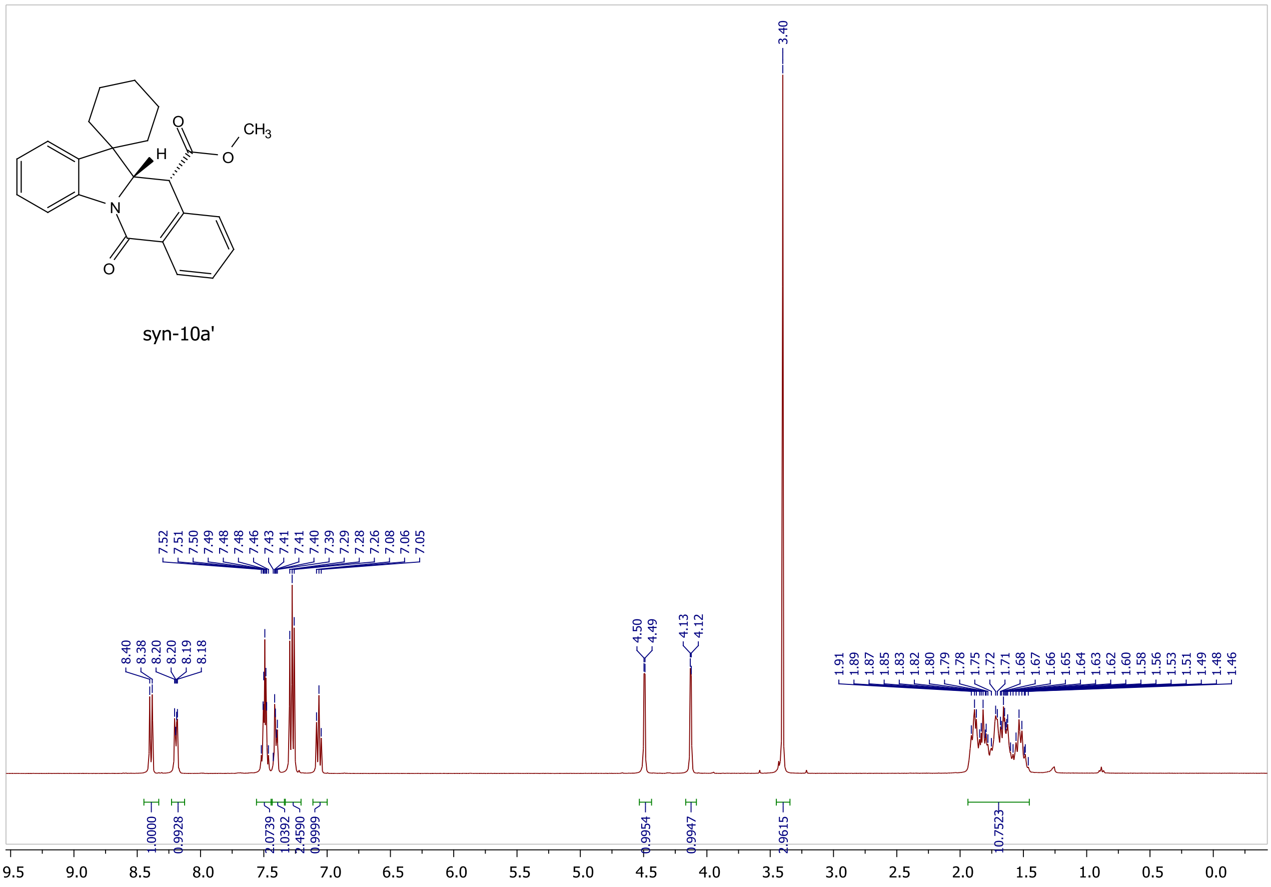
CH3
syn-10a'
7.52 7.51 7.50 7.49 7.48 7.48 7.46 7.43 7.41 7.41 7.40 7.39 7.29 7.28 7.26 7.08 7.06 7.05
8.40 8.38 8.20 8.20 8.19 8.18
4.50 4.49
4.13 4.12
3.40
1.91 1.89 1.87 1.85 1.83 1.82 1.80 1.79 1.78 1.75 1.72 1.71 1.68 1.67 1.66 1.65 1.64 1.63 1.62 1.60 1.58 1.56 1.53 1.51 1.49 1.48 1.46
1.0000 0.9928 2.0739 1.0392 2.4590 0.9999 0.9954 0.9947 2.9615 10.7523
9.5 9.0 8.5 8.0 7.5 7.0 6.5 6.0 5.5 5.0 4.5 4.0 3.5 3.0 2.5 2.0 1.5 1.0 0.5 0.0

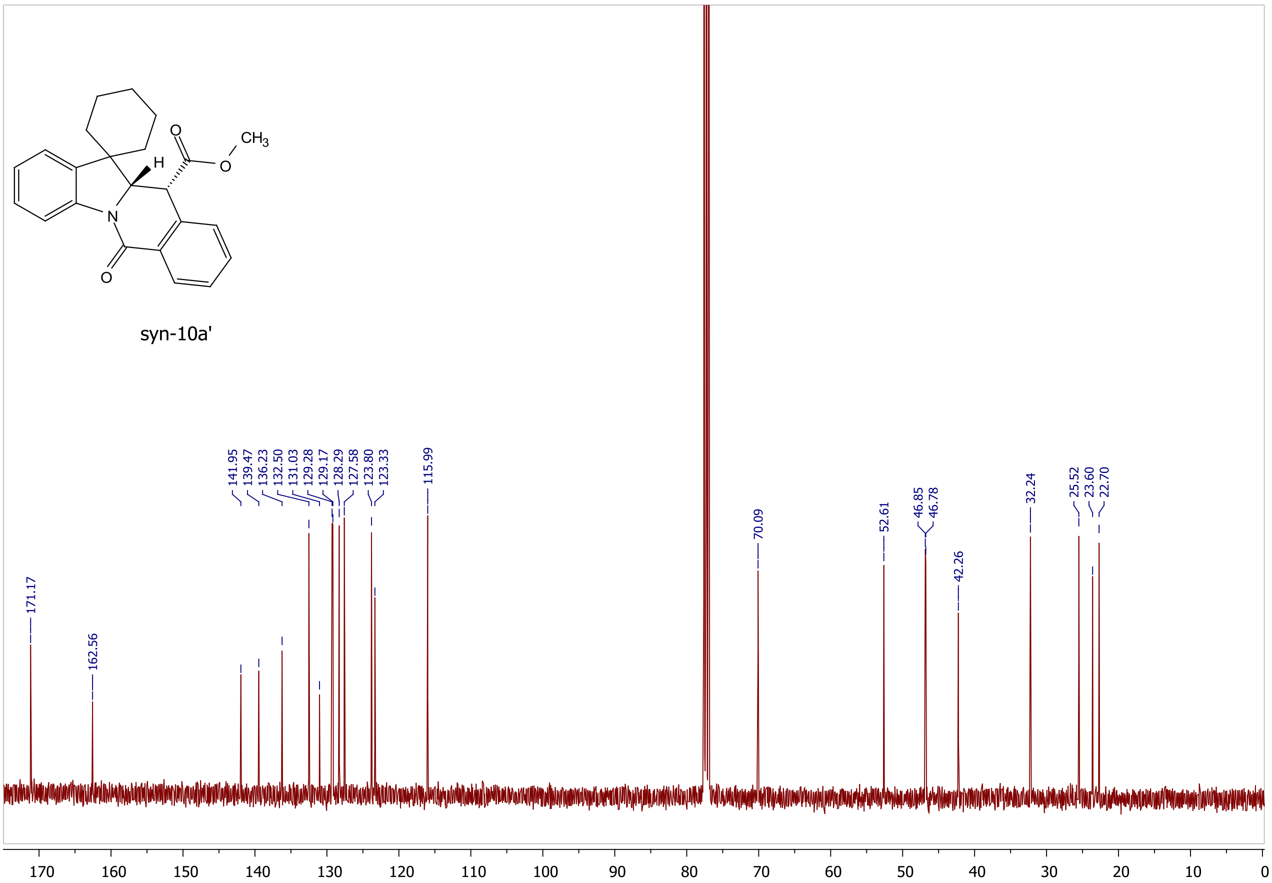
CH3
syn-10a'
171.17
162.56
141.95 139.47 136.23 132.50 131.03 129.28 129.17 128.29 127.58 123.80 123.33
115.99
70.09
52.61
46.85 46.78
42.26
32.24
25.52 23.60 22.70
170 160 150 140 130 120 110 100 90 80 70 60 50 40 30 20 10 0

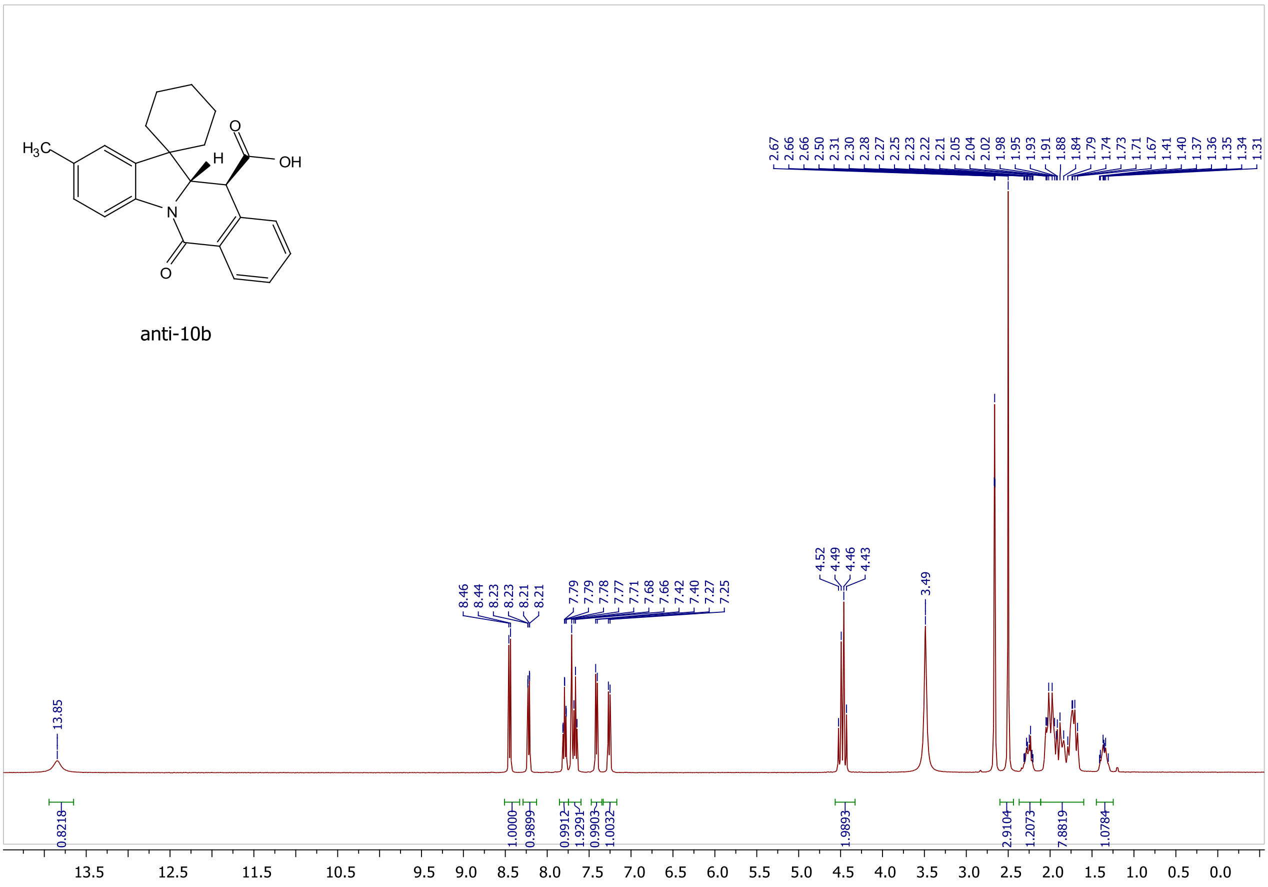
anti-10b
13.85
8.46 8.44 8.23 8.23 8.21 8.21
7.79 7.79 7.78 7.77 7.71 7.68 7.66 7.42 7.40 7.27 7.25
4.52 4.49 4.46 4.43
3.49
2.67 2.66 2.66 2.50 2.31 2.30 2.28 2.27 2.25 2.23 2.22 2.21 2.05 2.04 2.02 1.98 1.95 1.93 1.91 1.88 1.84 1.79 1.74 1.73 1.71 1.67 1.41 1.40 1.37 1.36 1.35 1.34 1.31
0.8218 1.0000 0.9899 0.9912 1.9291 0.9903 1.0032 1.9893 2.9104 1.2073 7.8819 1.0784

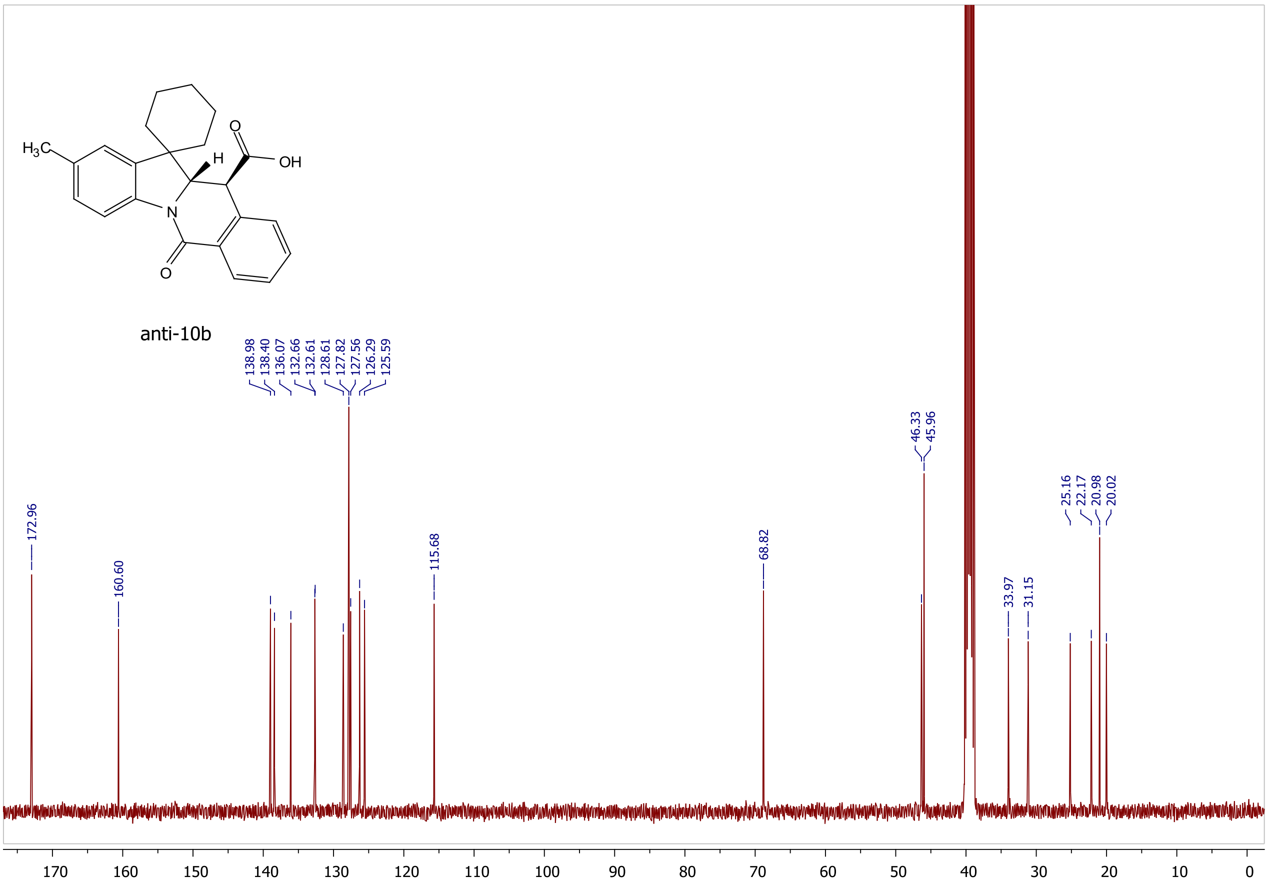
anti-10b
172.96
160.60
138.98 138.40 136.07 132.66 132.61 128.61 127.82 127.56 126.29 125.59
115.68
68.82
46.33 45.96
33.97 31.15
25.16 22.17 20.98 20.02

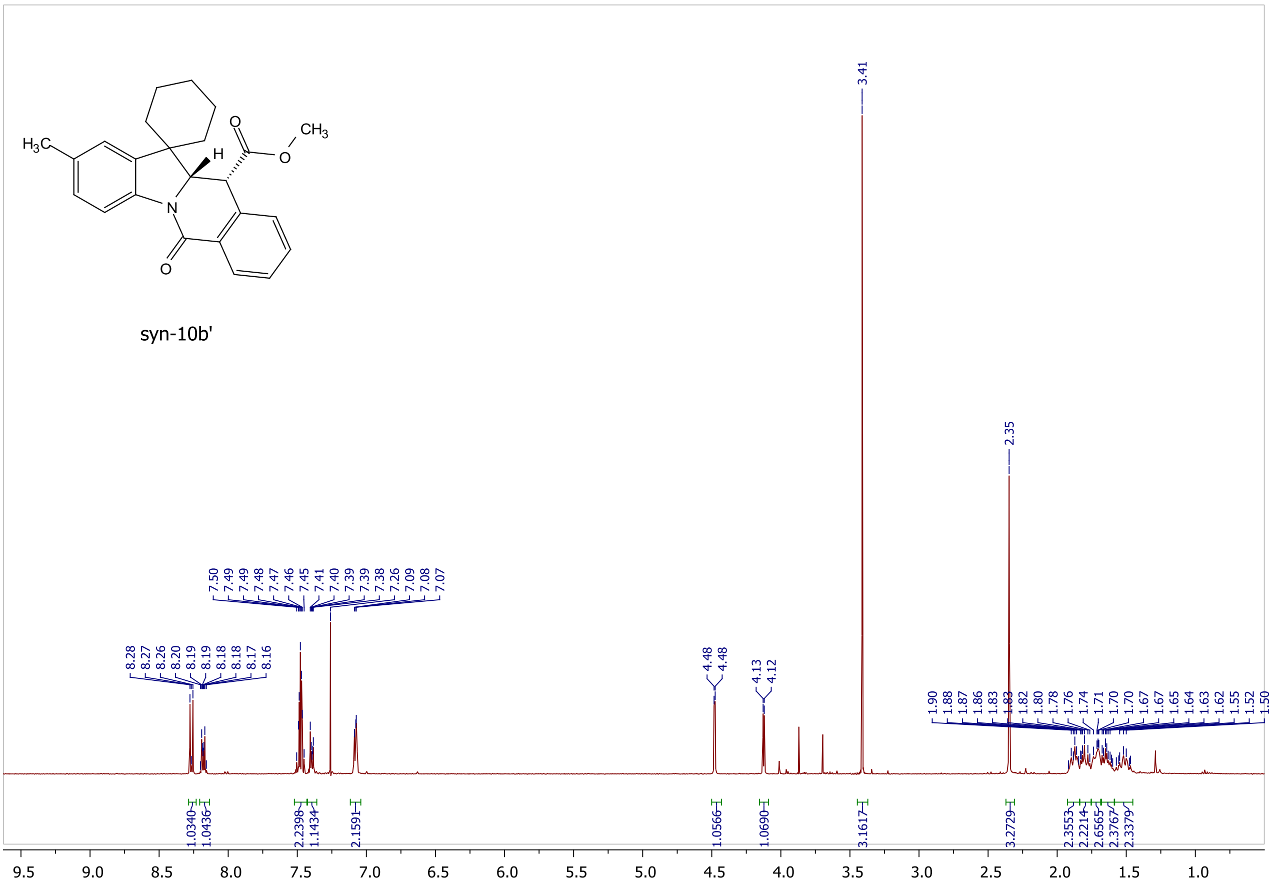
H3C
O
CH3
O
H
N
O
syn-10b'
3.41
2.35
7.50 7.49 7.49 7.48 7.47 7.46 7.45 7.41 7.40 7.39 7.39 7.38 7.26 7.09 7.08 7.07
8.28 8.27 8.26 8.20 8.19 8.19 8.18 8.18 8.17 8.16
4.48 4.48
4.13 4.12
1.90 1.88 1.87 1.86 1.83 1.83 1.82 1.80 1.78 1.76 1.74 1.71 1.70 1.70 1.67 1.67 1.65 1.64 1.63 1.62 1.55 1.52 1.50
1.0340 1.0436 2.2398 1.1434 2.1591 1.0566 1.0690 3.1617 3.2729 2.3553 2.2214 2.6565 2.3767 2.3379
9.5 9.0 8.5 8.0 7.5 7.0 6.5 6.0 5.5 5.0 4.5 4.0 3.5 3.0 2.5 2.0 1.5 1.0

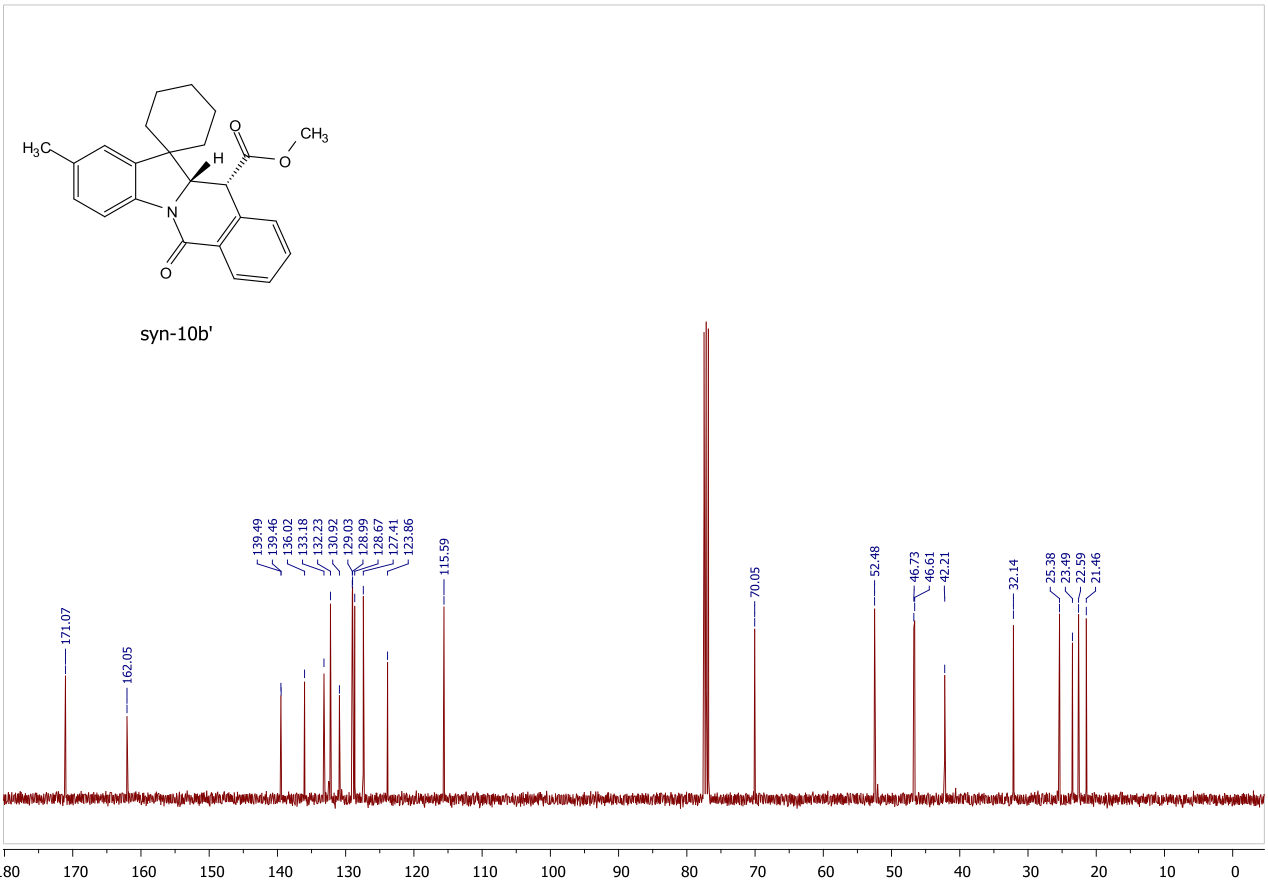
H3C
O
CH3
O
H
N
O
syn-10b'
171.07
162.05
139.49 139.46 136.02 133.18 132.23 130.92 129.03 128.99 128.67 127.41 123.86
115.59
70.05
52.48
46.73 46.61
42.21
32.14
25.38 23.49 22.59 21.46
180 170 160 150 140 130 120 110 100 90 80 70 60 50 40 30 20 10 0

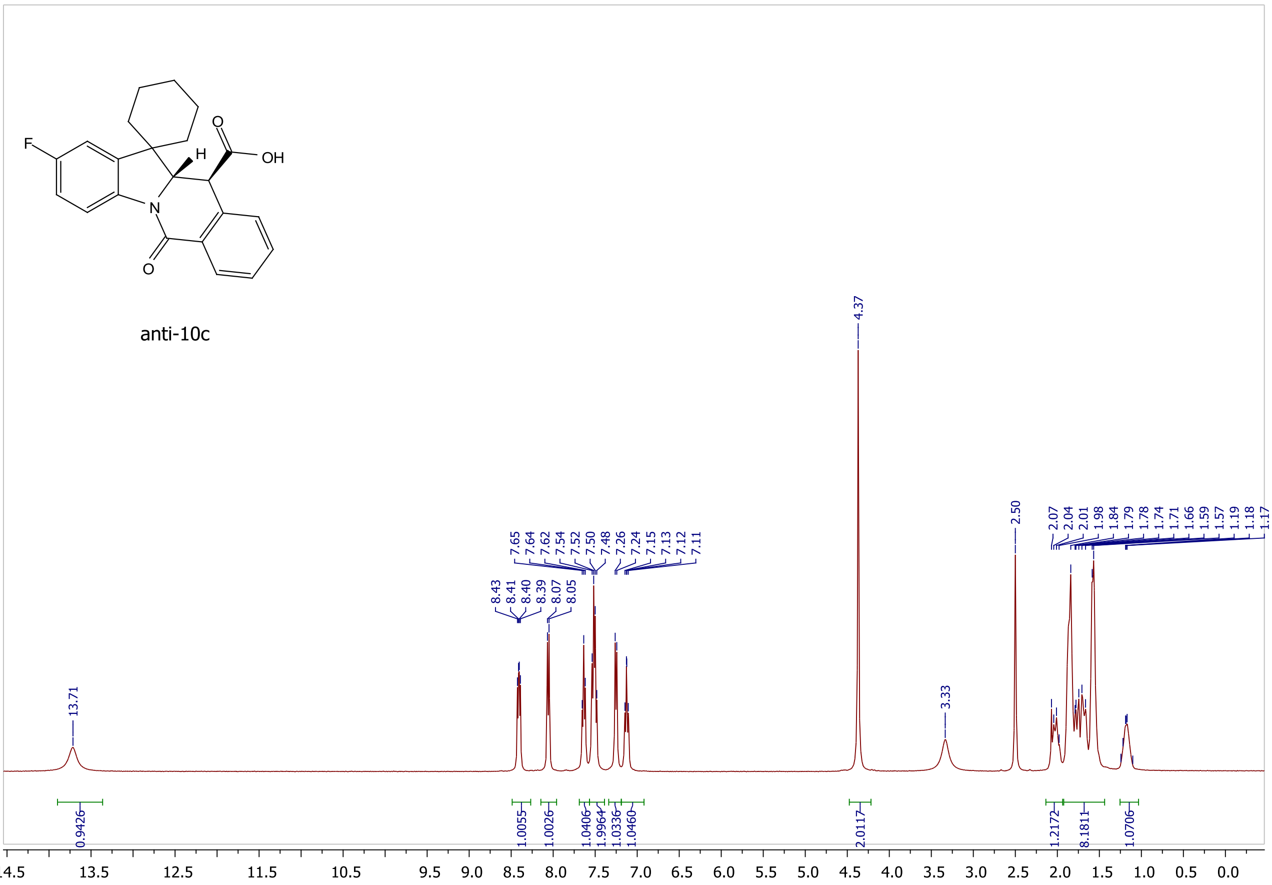
F
O
OH
H
N
O
anti-10c
13.71
8.43
8.41
8.40
8.39
8.07
8.05
7.65
7.64
7.62
7.54
7.52
7.50
7.48
7.26
7.24
7.15
7.13
7.12
7.11
4.37
3.33
2.50
2.07
2.04
2.01
1.98
1.84
1.79
1.78
1.74
1.71
1.66
1.59
1.57
1.19
1.18
1.17
0.9426
1.0055
1.0026
1.0406
1.9964
1.0336
1.0460
2.0117
1.2172
8.1811
1.0706
14.5 13.5 12.5 11.5 10.5 9.5 9.0 8.5 8.0 7.5 7.0 6.5 6.0 5.5 5.0 4.5 4.0 3.5 3.0 2.5 2.0 1.5 1.0 0.5 0.0

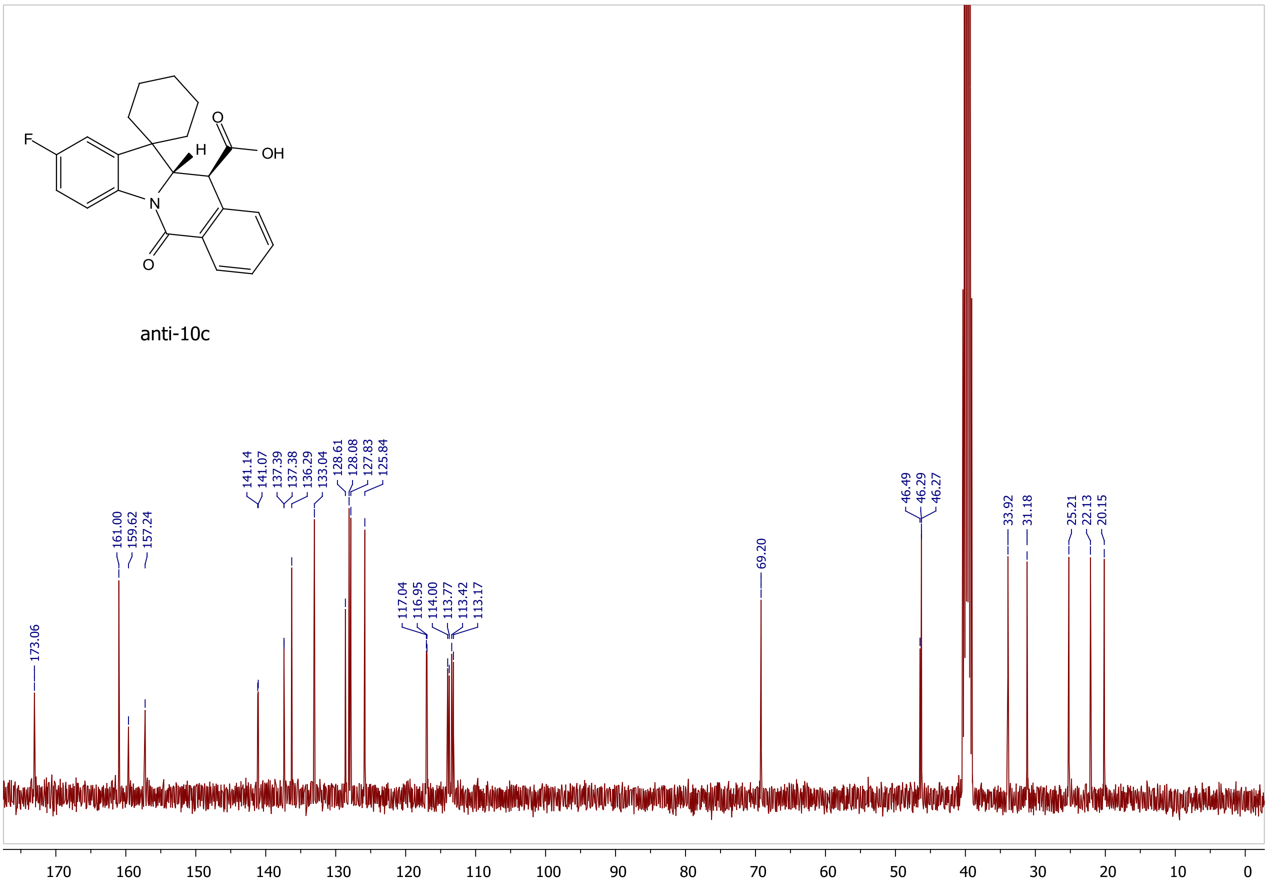
F
O
OH
H
N
O
anti-10c
173.06
161.00
159.62
157.24
141.14
141.07
137.39
137.38
136.29
133.04
128.61
128.08
127.83
125.84
117.04
116.95
114.00
113.77
113.42
113.17
69.20
46.49
46.29
46.27
33.92
31.18
25.21
22.13
20.15
170 160 150 140 130 120 110 100 90 80 70 60 50 40 30 20 10 0

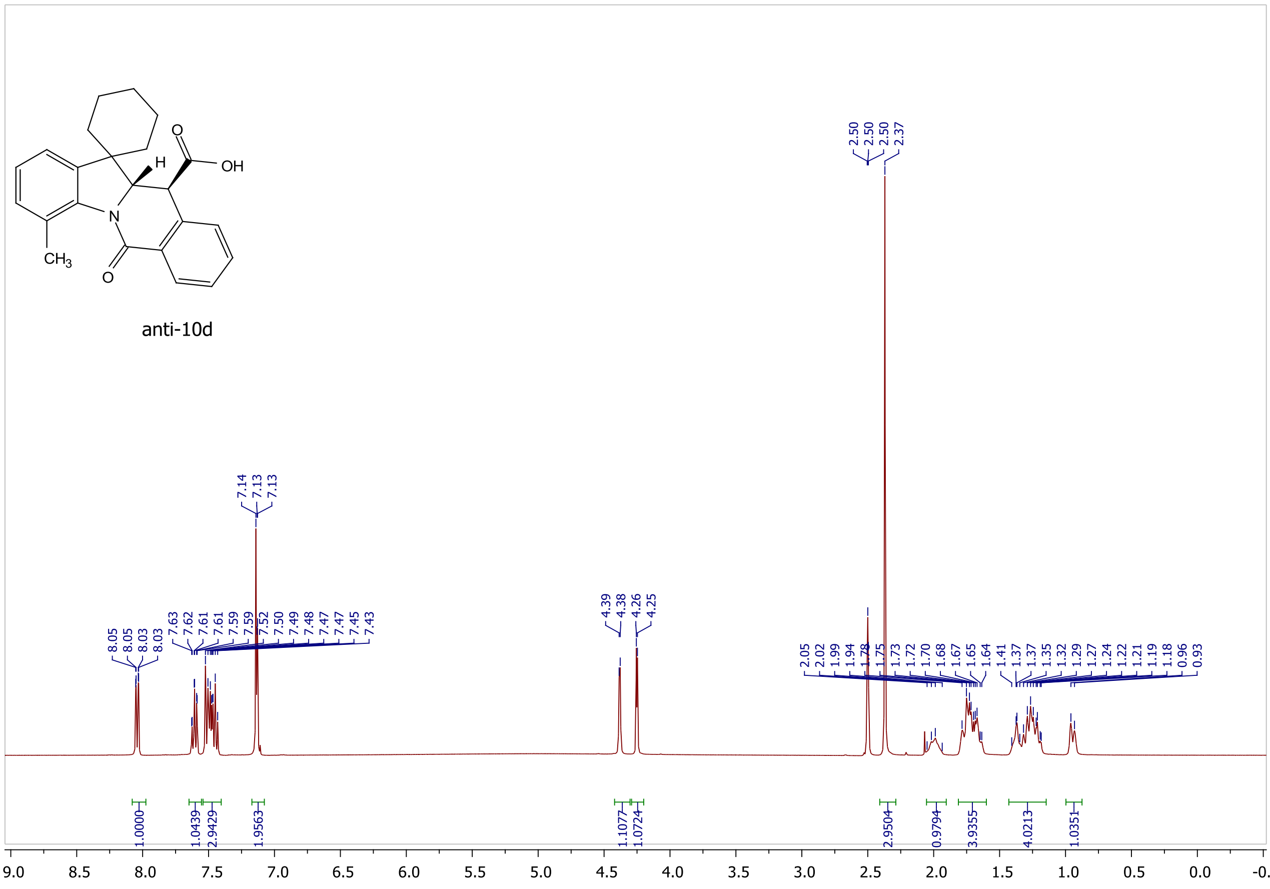

O
OH
H
N
CH3
O
anti-10d
8.05
8.05
8.03
8.03
7.63
7.62
7.61
7.61
7.59
7.59
7.52
7.50
7.49
7.48
7.47
7.47
7.45
7.43
7.14
7.13
7.13
4.39
4.38
4.26
4.25
2.50
2.50
2.50
2.37
1.0000
1.0439
2.9429
1.9563
1.1077
1.0724
2.9504
0.9794
3.9355
4.0213
1.0351
9.0
8.5
8.0
7.5
7.0
6.5
6.0
5.5
5.0
4.5
4.0
3.5
3.0
2.5
2.0
1.5
1.0
0.5
0.0
-0.5

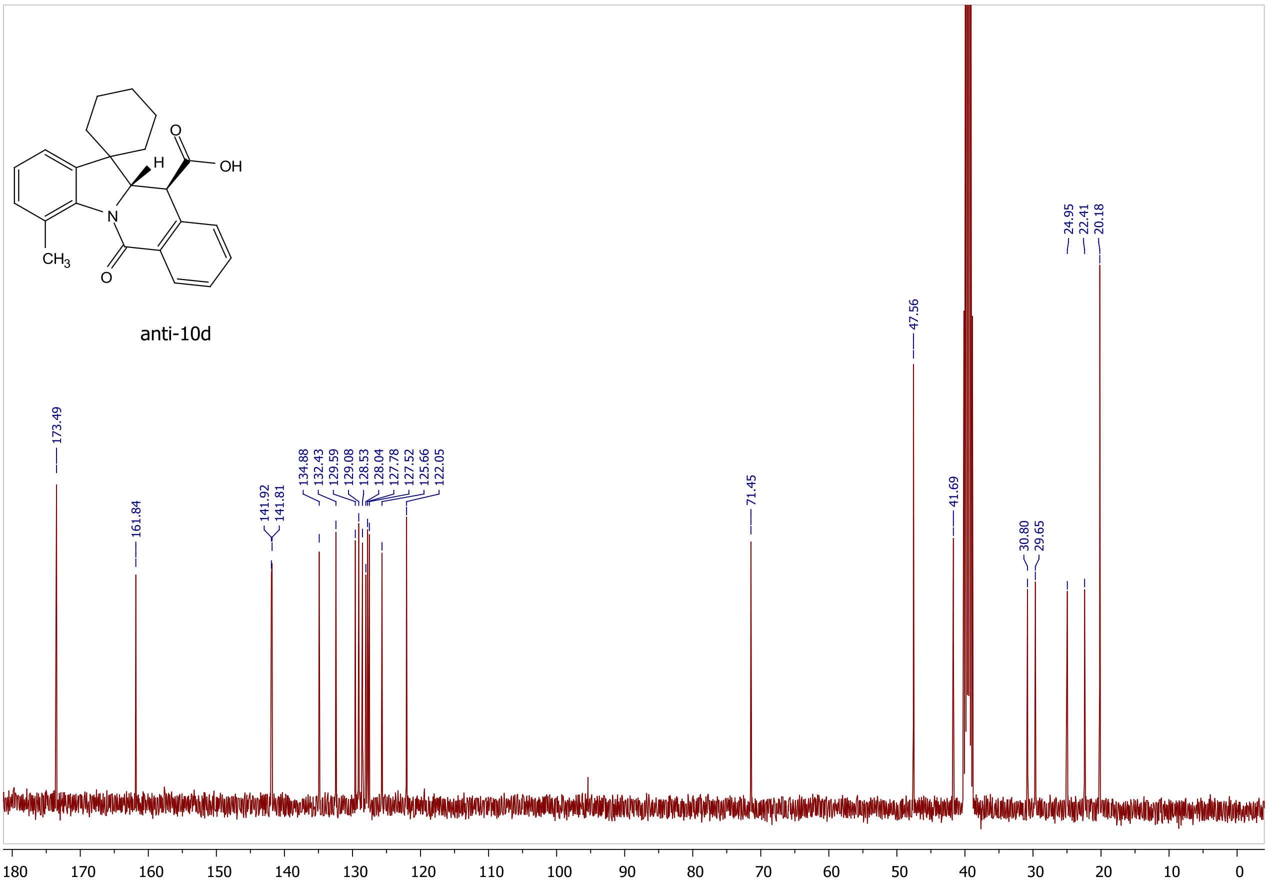

O
OH
H
N
CH3
O
anti-10d
173.49
161.84
141.92
141.81
134.88
132.43
129.59
129.08
128.53
128.04
127.78
127.52
125.66
122.05
71.45
47.56
41.69
30.80
29.65
24.95
22.41
20.18
180
170
160
150
140
130
120
110
100
90
80
70
60
50
40
30
20
10
0

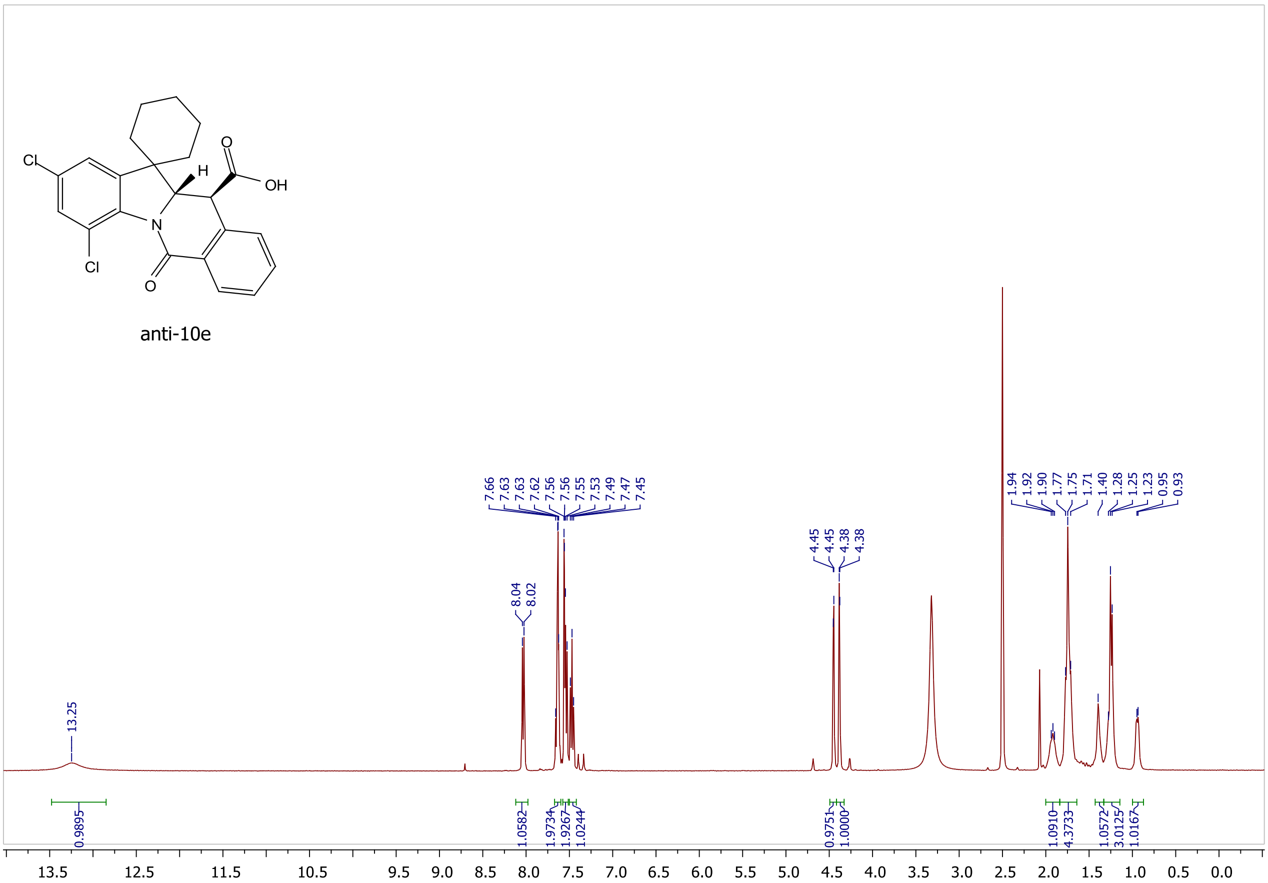
Cl
Cl
N
O
H
O
OH
anti-10e
13.25
8.04
8.02
7.66
7.63
7.63
7.62
7.56
7.56
7.55
7.53
7.49
7.47
7.45
4.45
4.45
4.38
4.38
1.94
1.92
1.90
1.77
1.75
1.71
1.40
1.28
1.25
1.23
0.95
0.93
0.9895
1.0582
1.9734
1.9267
1.0244
0.9751
1.0000
1.0910
4.3733
1.0572
3.0125
1.0167
13.5 12.5 11.5 10.5 9.5 9.0 8.5 8.0 7.5 7.0 6.5 6.0 5.5 5.0 4.5 4.0 3.5 3.0 2.5 2.0 1.5 1.0 0.5 0.0

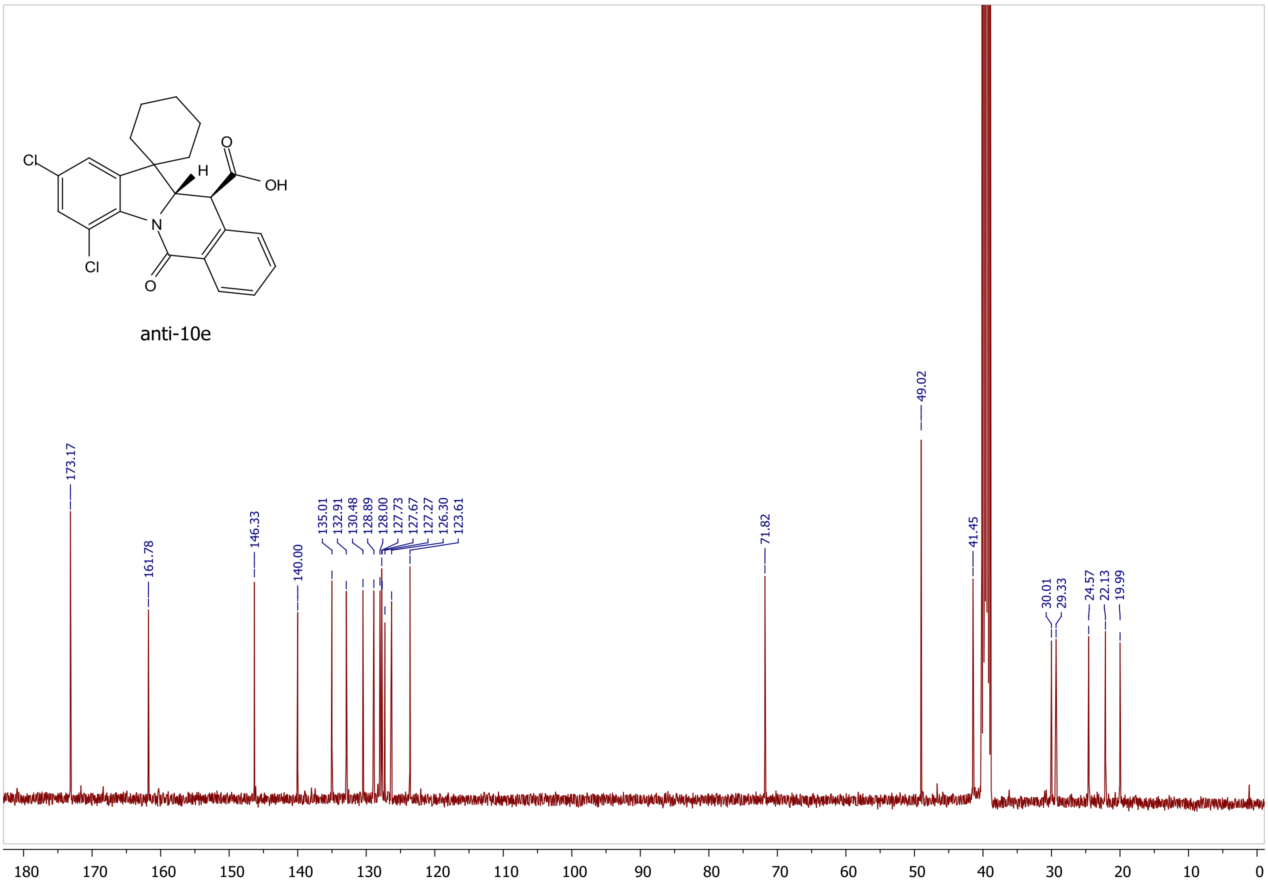
Cl
Cl
N
O
H
O
OH
anti-10e
173.17
161.78
146.33
140.00
135.01
132.91
130.48
128.89
128.00
127.73
127.67
127.27
126.30
123.61
71.82
49.02
41.45
30.01
29.33
24.57
22.13
19.99
180 170 160 150 140 130 120 110 100 90 80 70 60 50 40 30 20 10 0

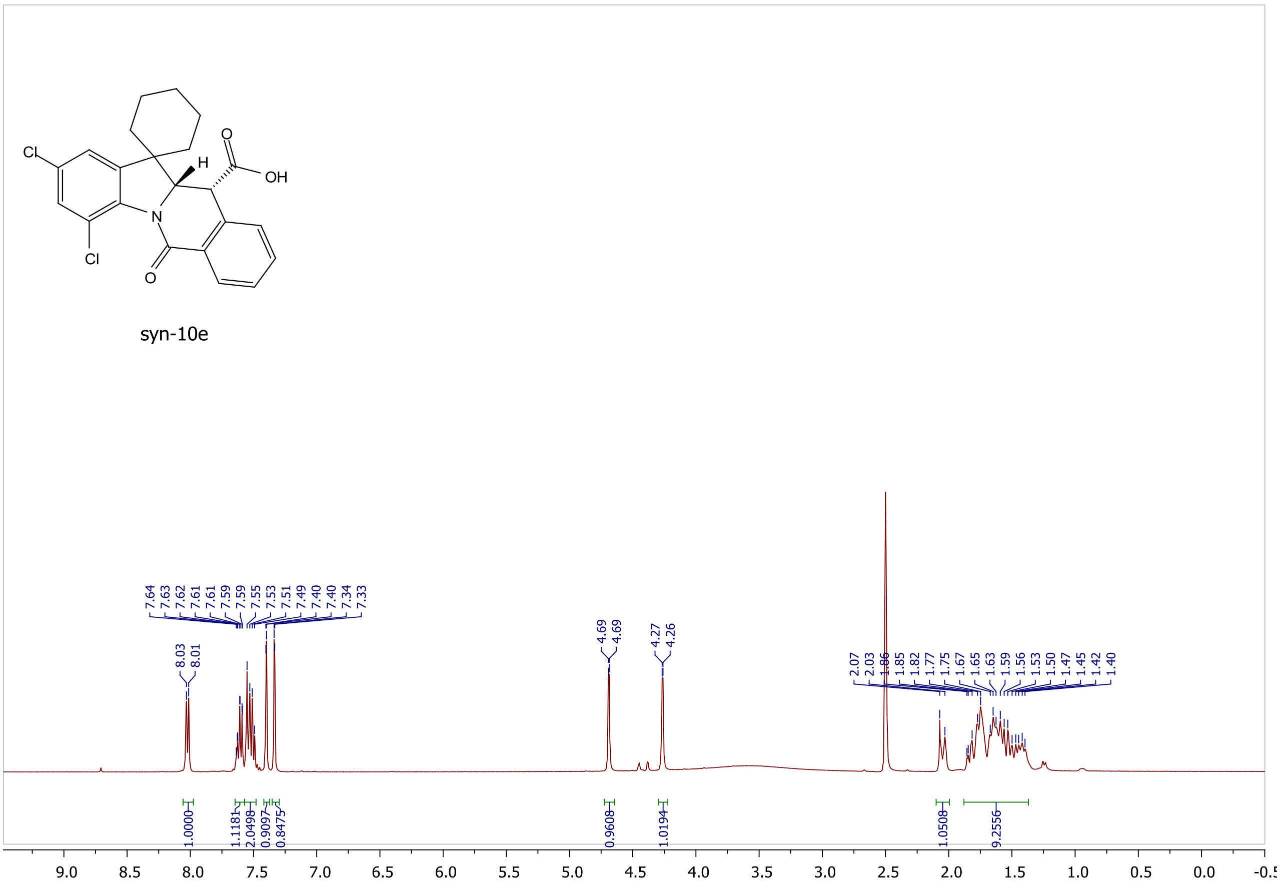
syn-10e
7.64 7.63 7.62 7.61 7.61 7.59 7.59 7.55 7.53 7.51 7.49 7.40 7.40 7.34 7.33
8.03 8.01
4.69 4.69
4.27 4.26
2.07 2.03 1.86 1.85 1.82 1.77 1.75 1.67 1.65 1.63 1.59 1.56 1.53 1.50 1.47 1.45 1.42 1.40
1.0000 1.1181 2.0498 0.9097 0.8475 0.9608 1.0194 1.0508 9.2556
9.0 8.5 8.0 7.5 7.0 6.5 6.0 5.5 5.0 4.5 4.0 3.5 3.0 2.5 2.0 1.5 1.0 0.5 0.0

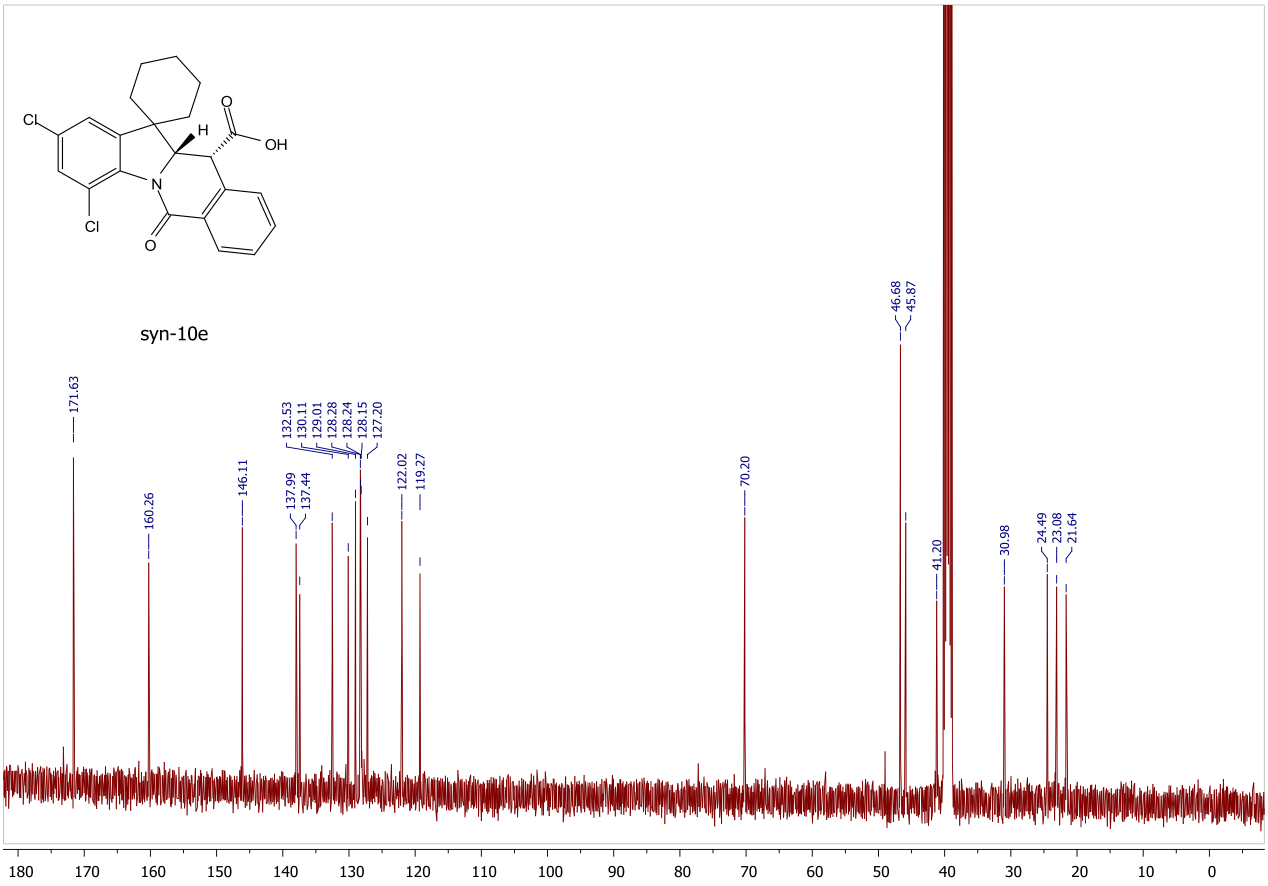
syn-10e
171.63
160.26
146.11
137.99 137.44
132.53 130.11 129.01 128.28 128.24 128.15 127.20
122.02 119.27
70.20
46.68 45.87
41.20
30.98
24.49 23.08 21.64
180 170 160 150 140 130 120 110 100 90 80 70 60 50 40 30 20 10 0

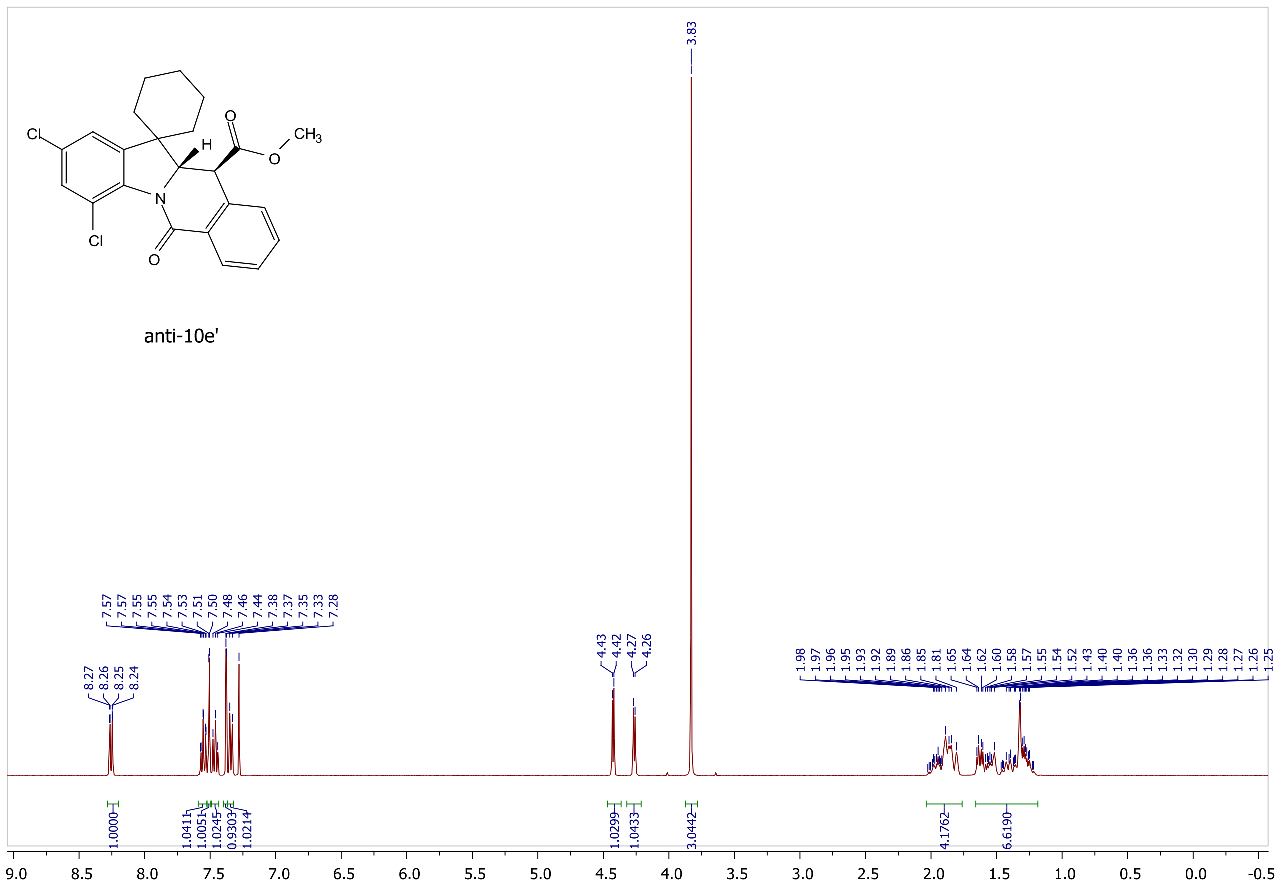

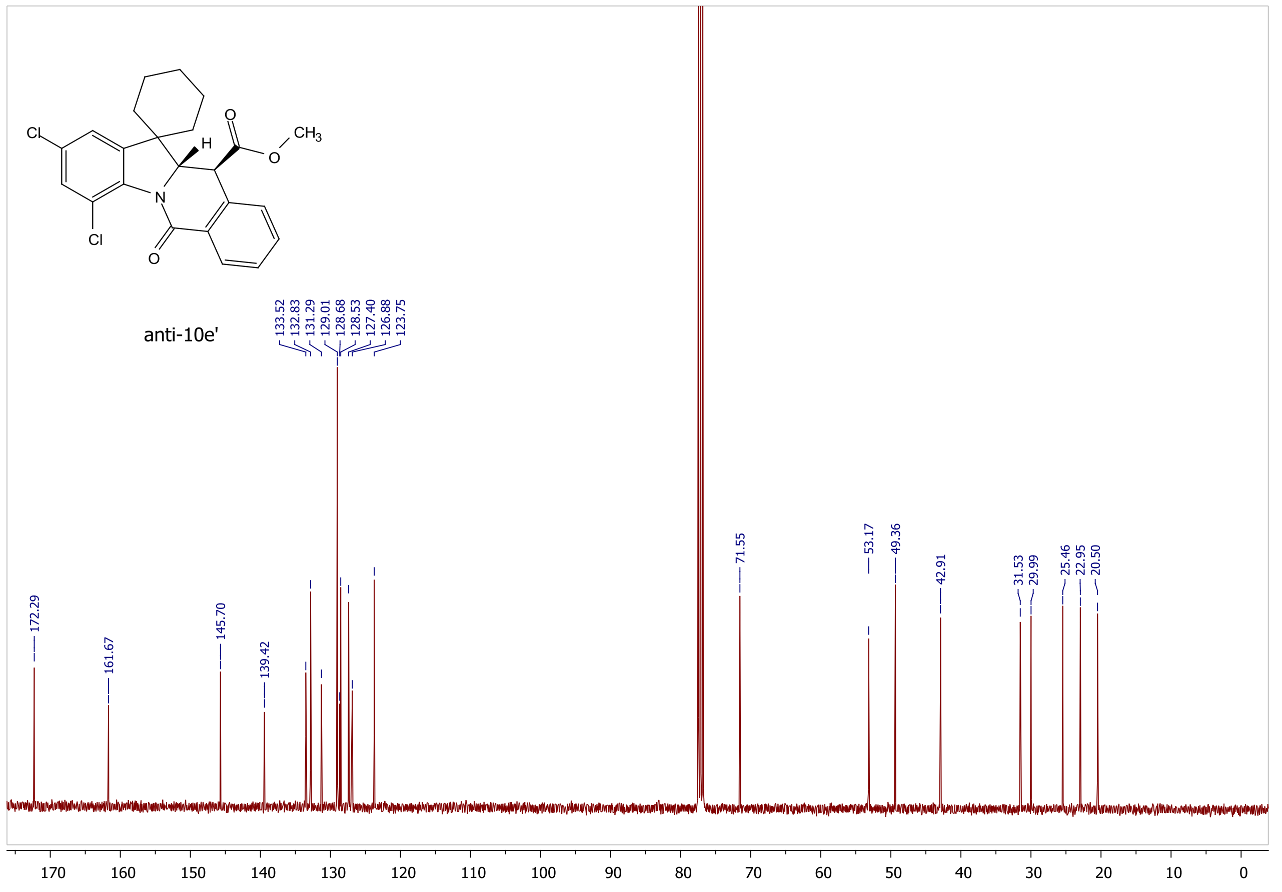

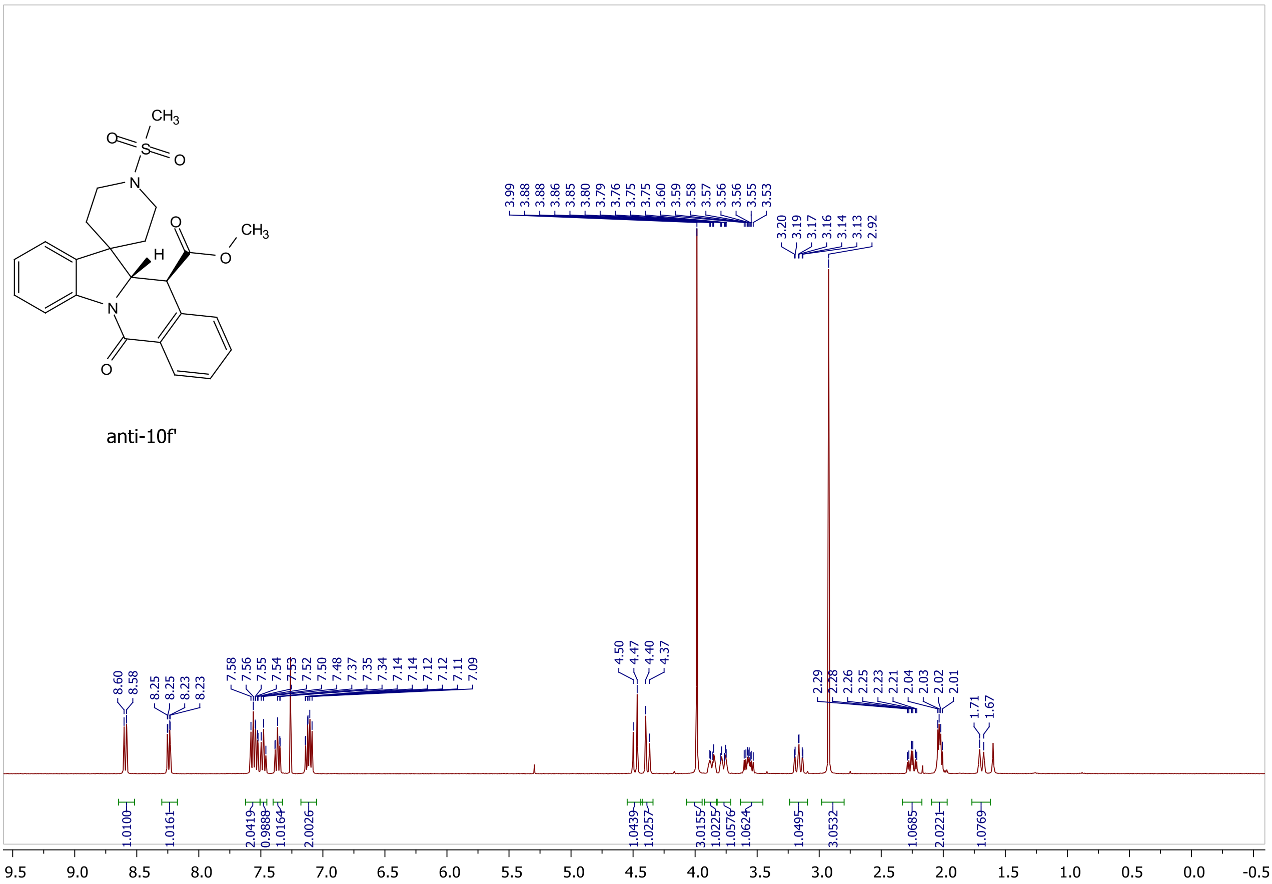
CH3
O
S
O
N
O
CH3
H
O
N
O
anti-10f'
3.99 3.88 3.88 3.86 3.85 3.80 3.79 3.76 3.75 3.75 3.60 3.59 3.58 3.57 3.56 3.56 3.55 3.53
3.20 3.19 3.17 3.16 3.14 3.13 2.92
8.60 8.58 8.25 8.25 8.23 8.23
7.58 7.56 7.55 7.54 7.53 7.52 7.50 7.48 7.37 7.35 7.34 7.14 7.14 7.12 7.12 7.11 7.09
4.50 4.47 4.40 4.37
2.29 2.28 2.26 2.25 2.23 2.21 2.04 2.03 2.02 2.01
1.71 1.67
1.0100 1.0161 2.0419 0.9888 1.0164 2.0026 1.0439 1.0257 3.0155 1.0225 1.0576 1.0624 1.0495 3.0532 1.0685 2.0221 1.0769
9.5 9.0 8.5 8.0 7.5 7.0 6.5 6.0 5.5 5.0 4.5 4.0 3.5 3.0 2.5 2.0 1.5 1.0 0.5 0.0 -0.5

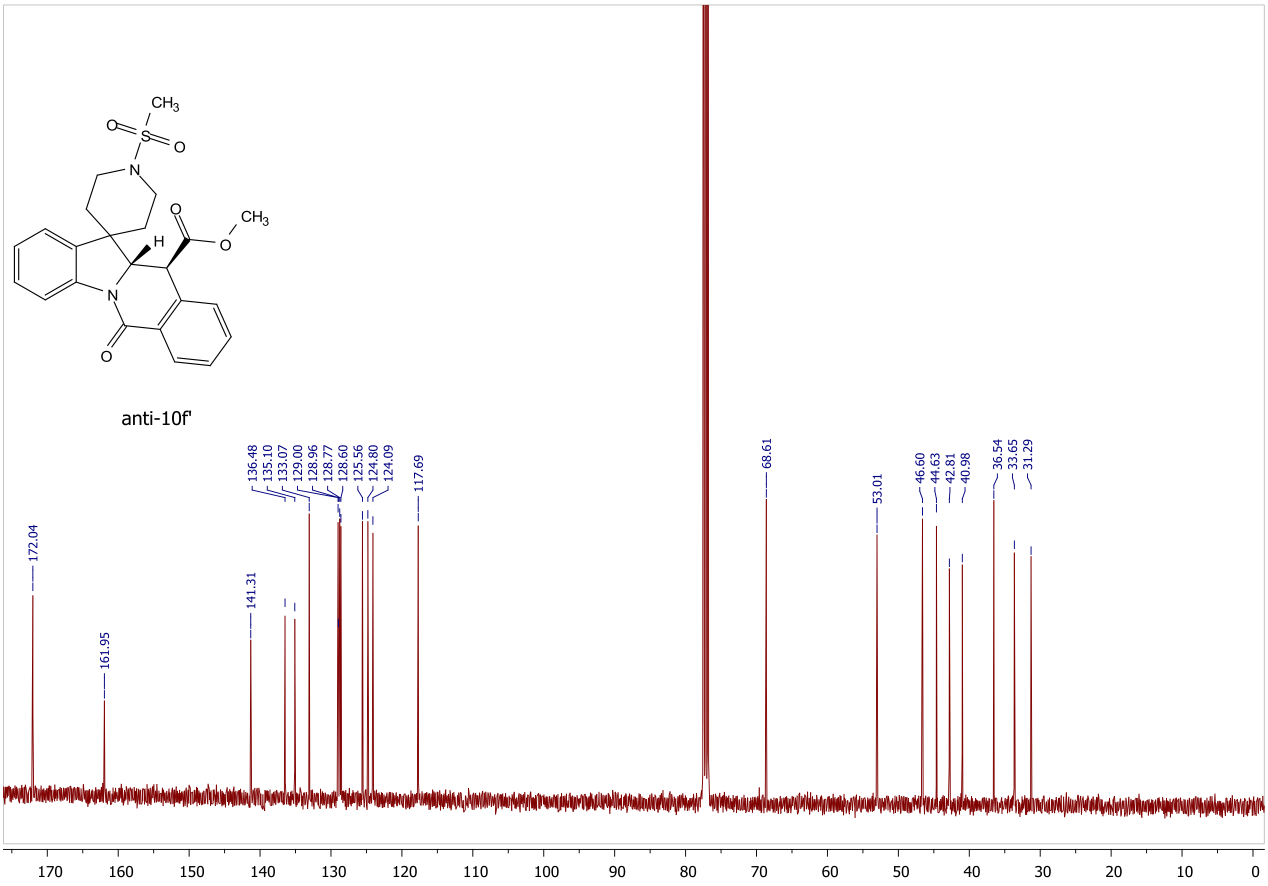
CH3
O
S
O
N
O
CH3
H
O
N
O
anti-10f'
172.04
161.95
141.31
136.48 135.10 133.07 129.00 128.96 128.77 128.60 125.56 124.80 124.09
117.69
68.61
53.01
46.60 44.63 42.81 40.98
36.54 33.65 31.29
170 160 150 140 130 120 110 100 90 80 70 60 50 40 30 20 10 0

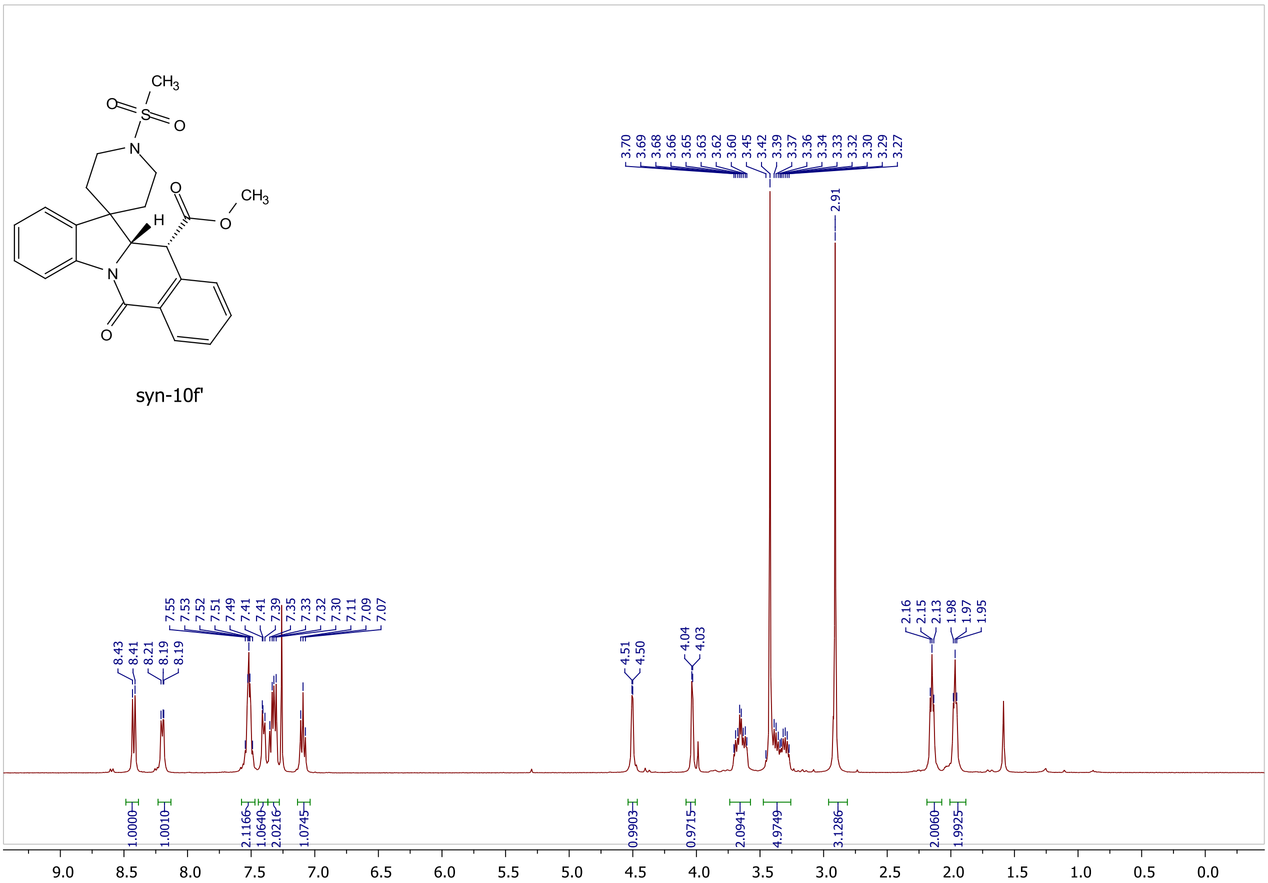
syn-10f'
3.70
3.69
3.68
3.66
3.65
3.63
3.62
3.60
3.45
3.42
3.39
3.37
3.36
3.34
3.33
3.32
3.30
3.29
3.27
2.91
8.43
8.41
8.21
8.19
8.19
7.55
7.53
7.52
7.51
7.49
7.41
7.41
7.39
7.35
7.33
7.32
7.30
7.11
7.09
7.07
4.51
4.50
4.04
4.03
2.16
2.15
2.13
1.98
1.97
1.95
1.0000
1.0010
2.1166
1.0640
2.0216
1.0745
0.9903
0.9715
2.0941
4.9749
3.1286
2.0060
1.9925

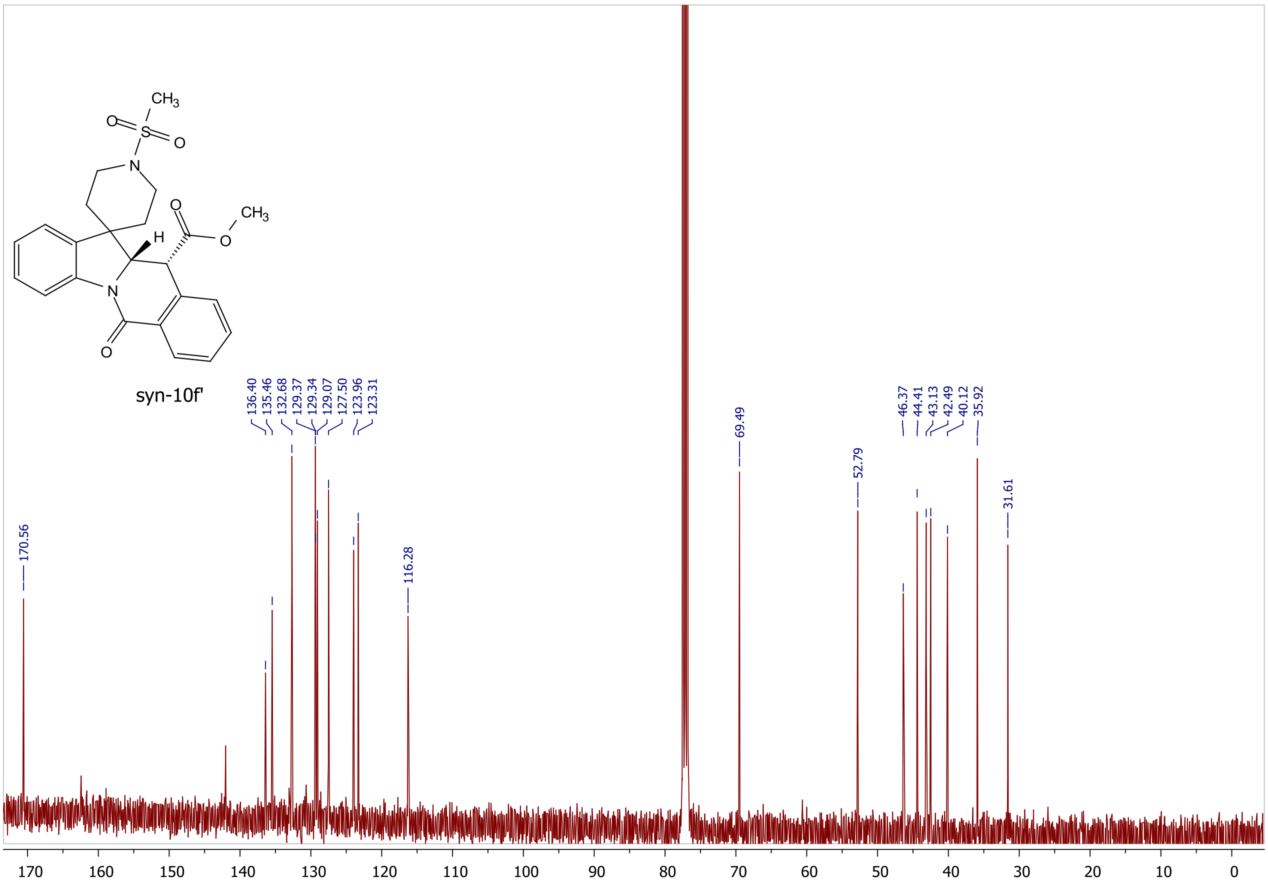
syn-10f'
170.56
136.40
135.46
132.68
129.37
129.34
129.07
127.50
123.96
123.31
116.28
69.49
52.79
46.37
44.41
43.13
42.49
40.12
35.92
31.61

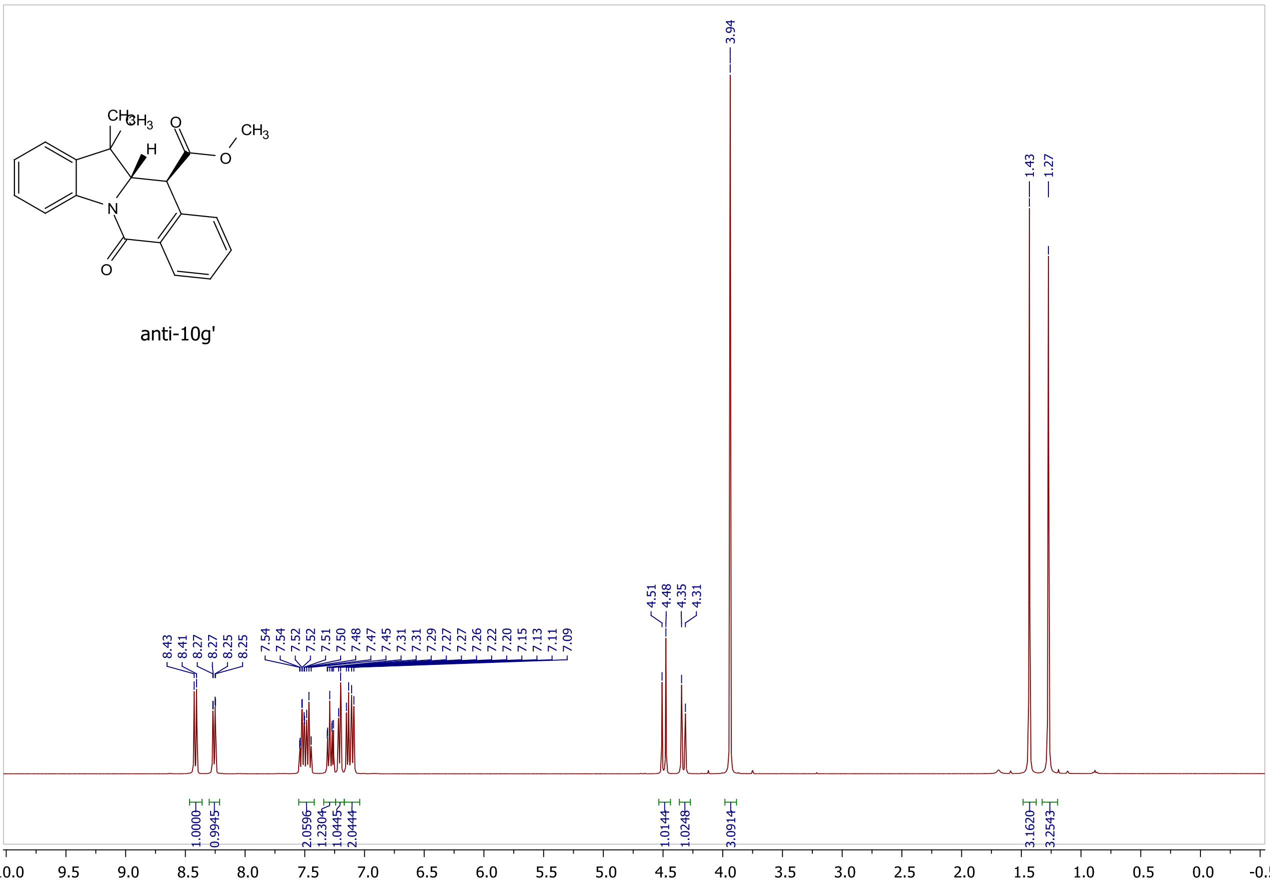
anti-10g'
3.94
1.43
1.27
4.51
4.48
4.35
4.31
8.43
8.41
8.27
8.27
8.25
8.25
1.0000
0.9945
2.0596
1.2304
1.0445
2.0444
1.0144
1.0248
3.0914
3.1620
3.2543

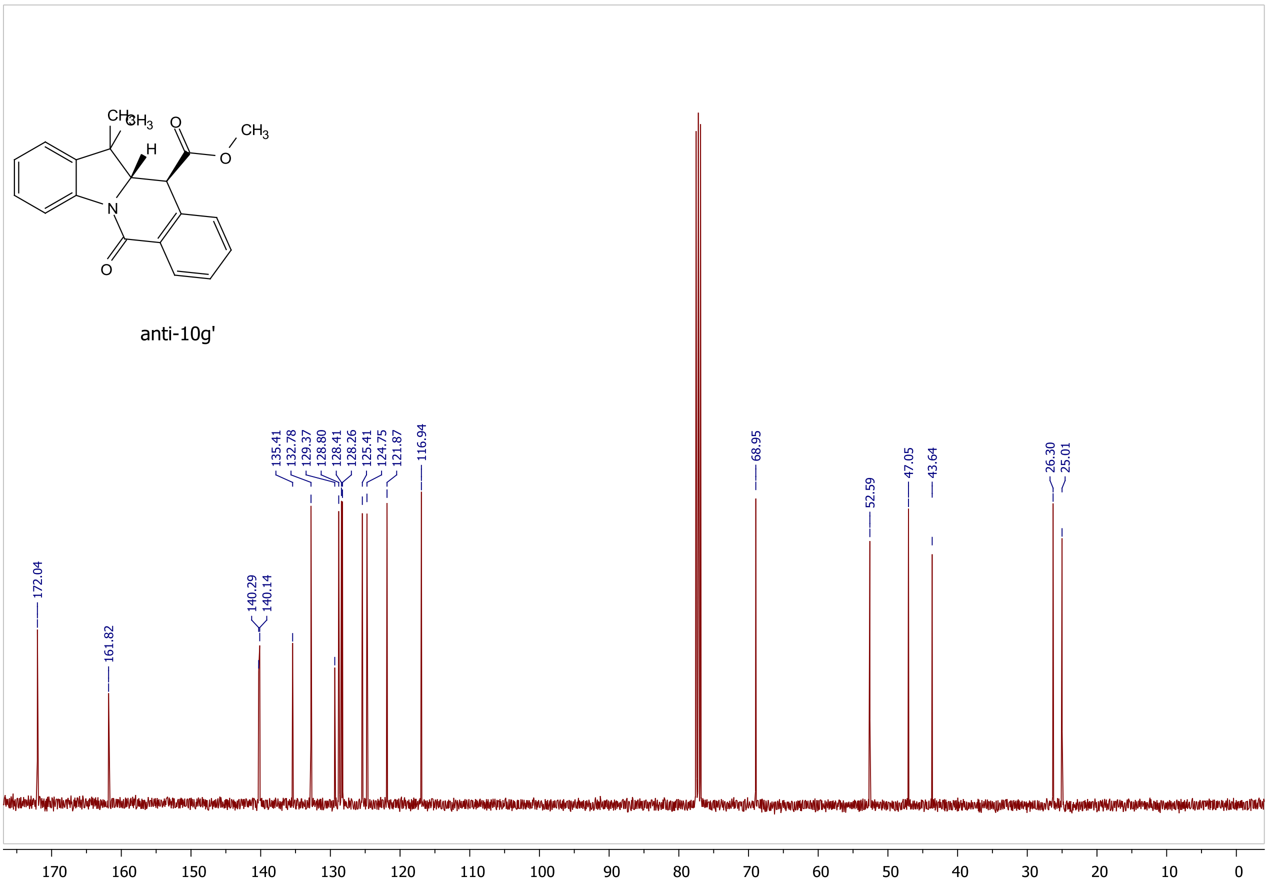
anti-10g'
172.04
161.82
140.29
140.14
135.41
132.78
129.37
128.80
128.41
128.26
125.41
124.75
121.87
116.94
68.95
52.59
47.05
43.64
26.30
25.01

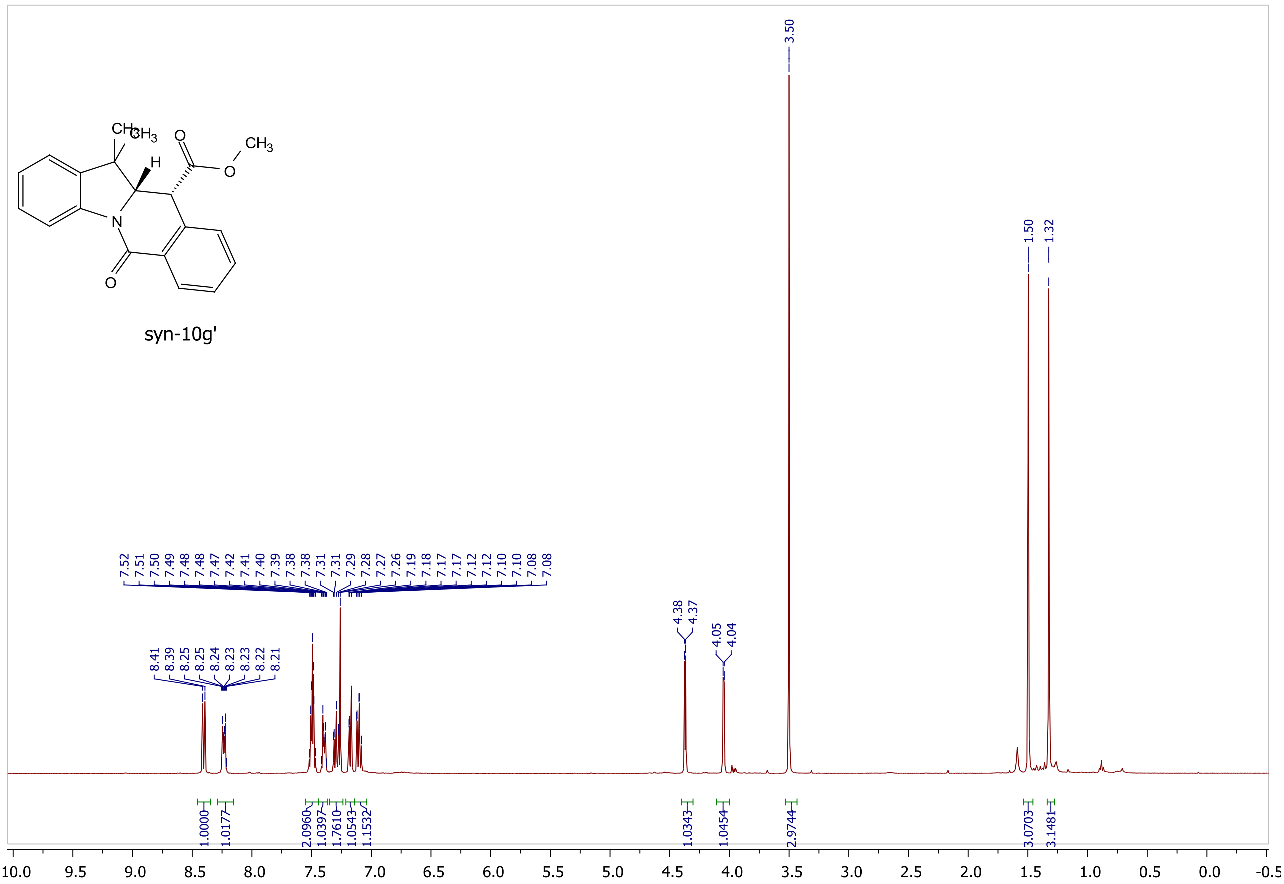
CH3
CH3
H
O
O
CH3
N
O
syn-10g'
8.41 8.39 8.25 8.25 8.24 8.23 8.23 8.22 8.21
7.52 7.51 7.50 7.49 7.48 7.48 7.47 7.42 7.41 7.40 7.39 7.38 7.38 7.31 7.31 7.29 7.28 7.27 7.26 7.19 7.18 7.17 7.17 7.12 7.12 7.10 7.10 7.08 7.08
4.38 4.37
4.05 4.04
3.50
1.50
1.32
1.0000 1.0177 2.0960 1.0397 1.7610 1.0543 1.1532 1.0343 1.0454 2.9744 3.0703 3.1481
10.0 9.5 9.0 8.5 8.0 7.5 7.0 6.5 6.0 5.5 5.0 4.5 4.0 3.5 3.0 2.5 2.0 1.5 1.0 0.5 0.0 -0.5

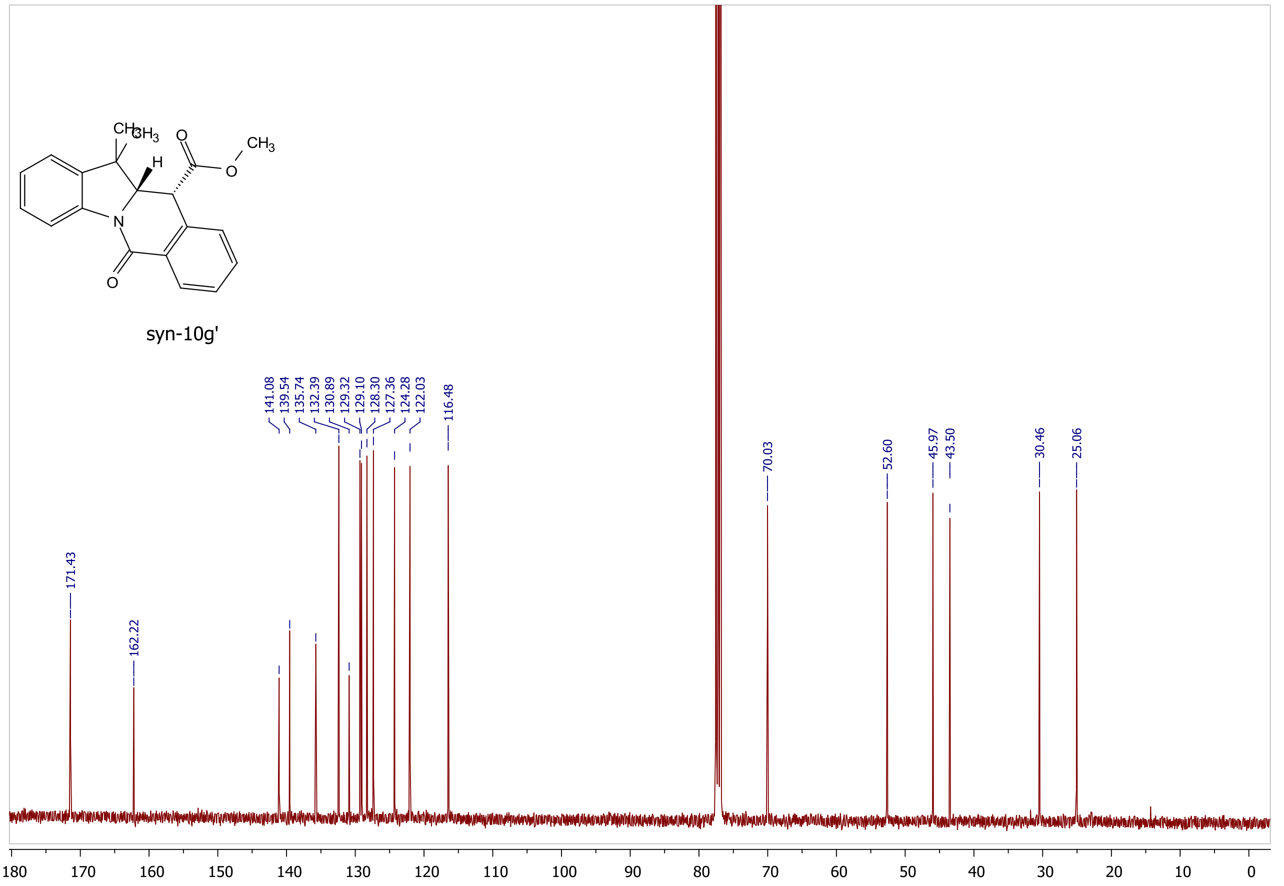
CH3
CH3
H
O
O
CH3
N
O
syn-10g'
171.43
162.22
141.08 139.54 135.74 132.39 130.89 129.32 129.10 128.30 127.36 124.28 122.03
116.48
70.03
52.60
45.97
43.50
30.46
25.06
180 170 160 150 140 130 120 110 100 90 80 70 60 50 40 30 20 10 0

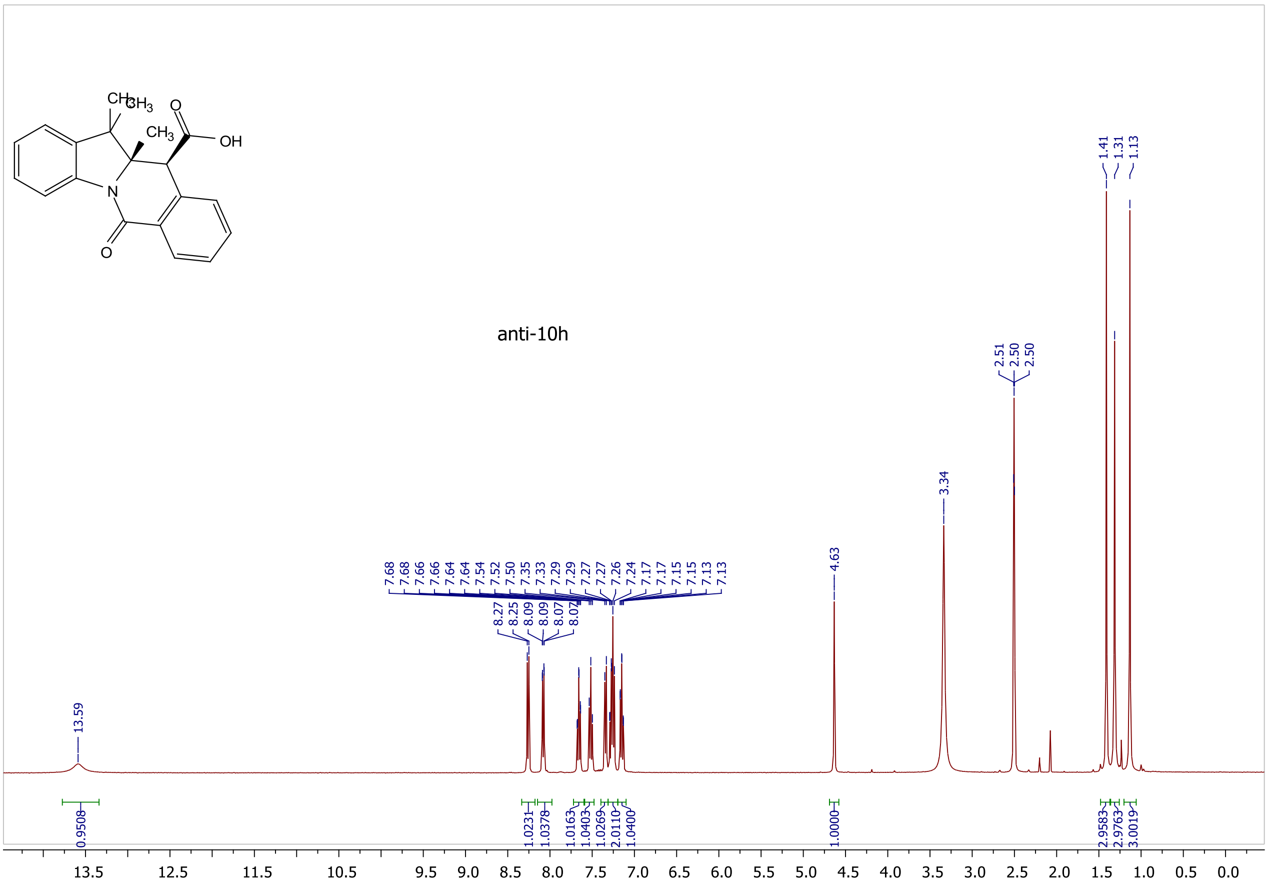
CH3
CH3
O
OH
N
O
anti-10h
1.41
1.31
1.13
2.51
2.50
2.50
3.34
4.63
7.68
7.68
7.66
7.66
7.64
7.64
7.54
7.52
7.50
7.35
7.33
7.29
7.29
7.27
7.27
7.26
7.24
7.17
7.17
7.15
7.15
7.13
7.13
8.27
8.25
8.09
8.09
8.07
8.07
13.59
0.9508
1.0231
1.0378
1.0163
1.0403
1.0269
2.0110
1.0400
1.0000
2.9583
2.9763
3.0019
13.5
12.5
11.5
10.5
9.5
9.0
8.5
8.0
7.5
7.0
6.5
6.0
5.5
5.0
4.5
4.0
3.5
3.0
2.5
2.0
1.5
1.0
0.5
0.0

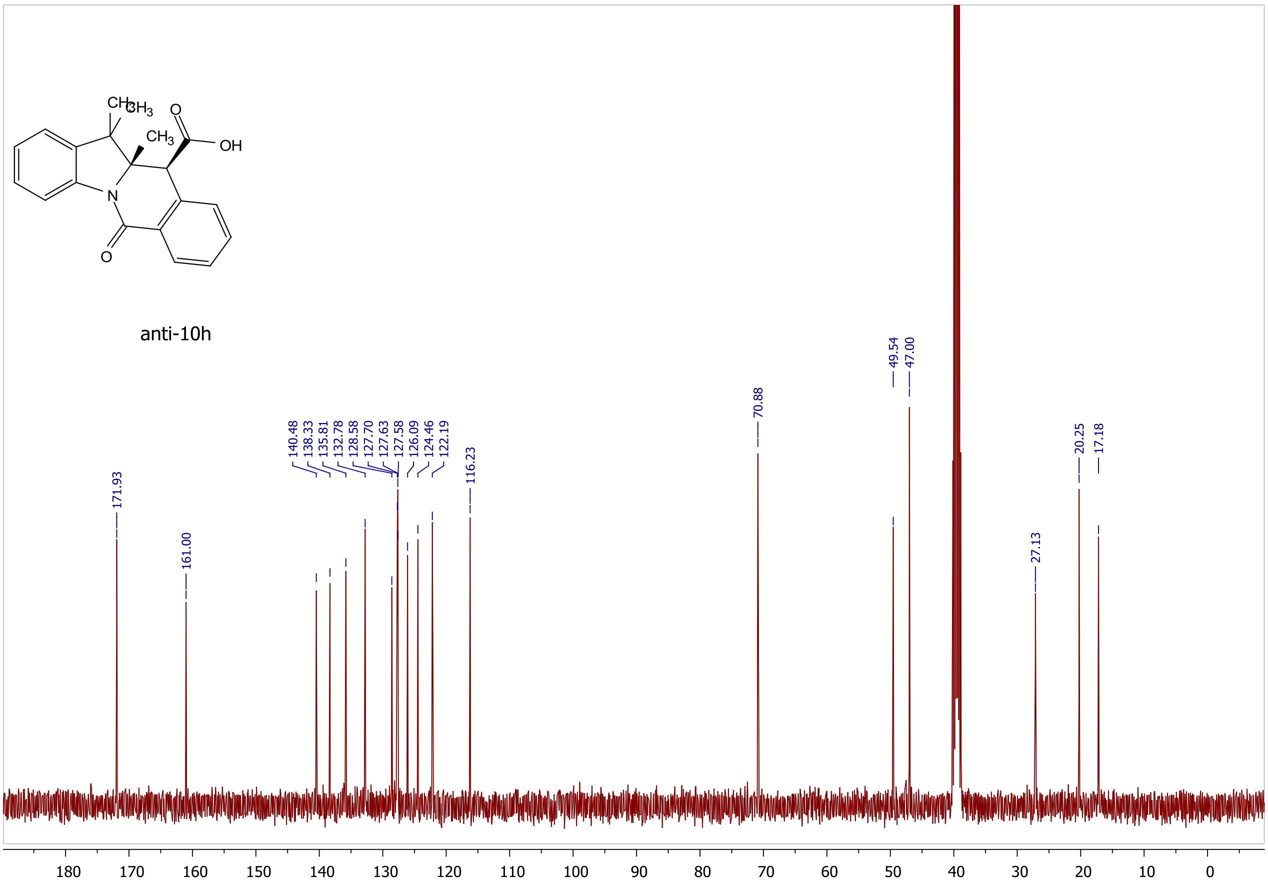
CH3
CH3
O
OH
N
O
anti-10h
171.93
161.00
140.48
138.33
135.81
132.78
128.58
127.70
127.63
127.58
126.09
124.46
122.19
116.23
70.88
49.54
47.00
27.13
20.25
17.18
180
170
160
150
140
130
120
110
100
90
80
70
60
50
40
30
20
10
0

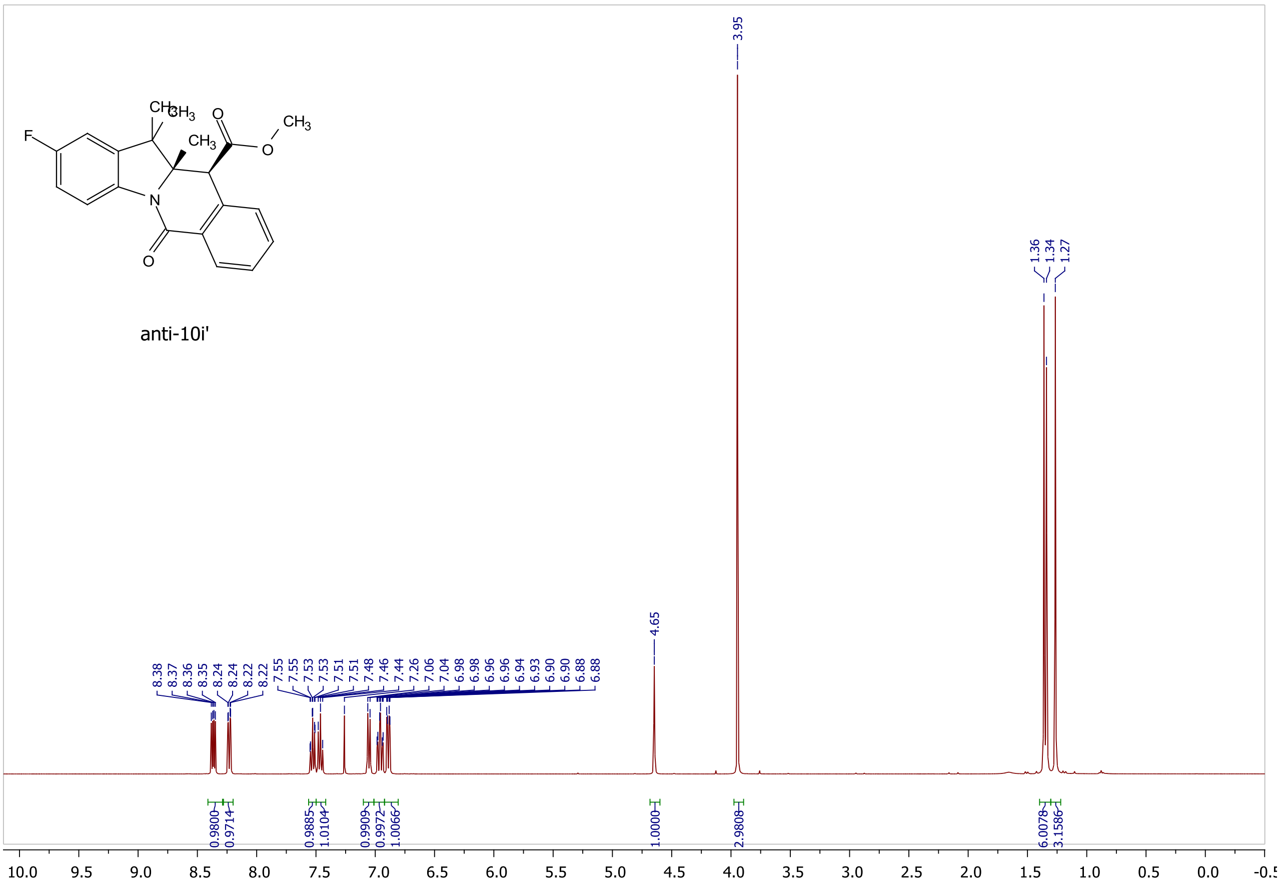
F
CH3 CH3
CH3
O
O
CH3
N
O
anti-10i'
8.38 8.37 8.36 8.35 8.24 8.24 8.22 8.22 7.55 7.55 7.53 7.53 7.51 7.51 7.48 7.46 7.44 7.26 7.06 7.04 6.98 6.98 6.96 6.96 6.94 6.93 6.90 6.90 6.88 6.88
4.65
3.95
1.36 1.34 1.27
0.9800 0.9714 0.9885 1.0104 0.9909 0.9972 1.0066 1.0000 2.9808 6.0078 3.1586
10.0 9.5 9.0 8.5 8.0 7.5 7.0 6.5 6.0 5.5 5.0 4.5 4.0 3.5 3.0 2.5 2.0 1.5 1.0 0.5 0.0 -0.5

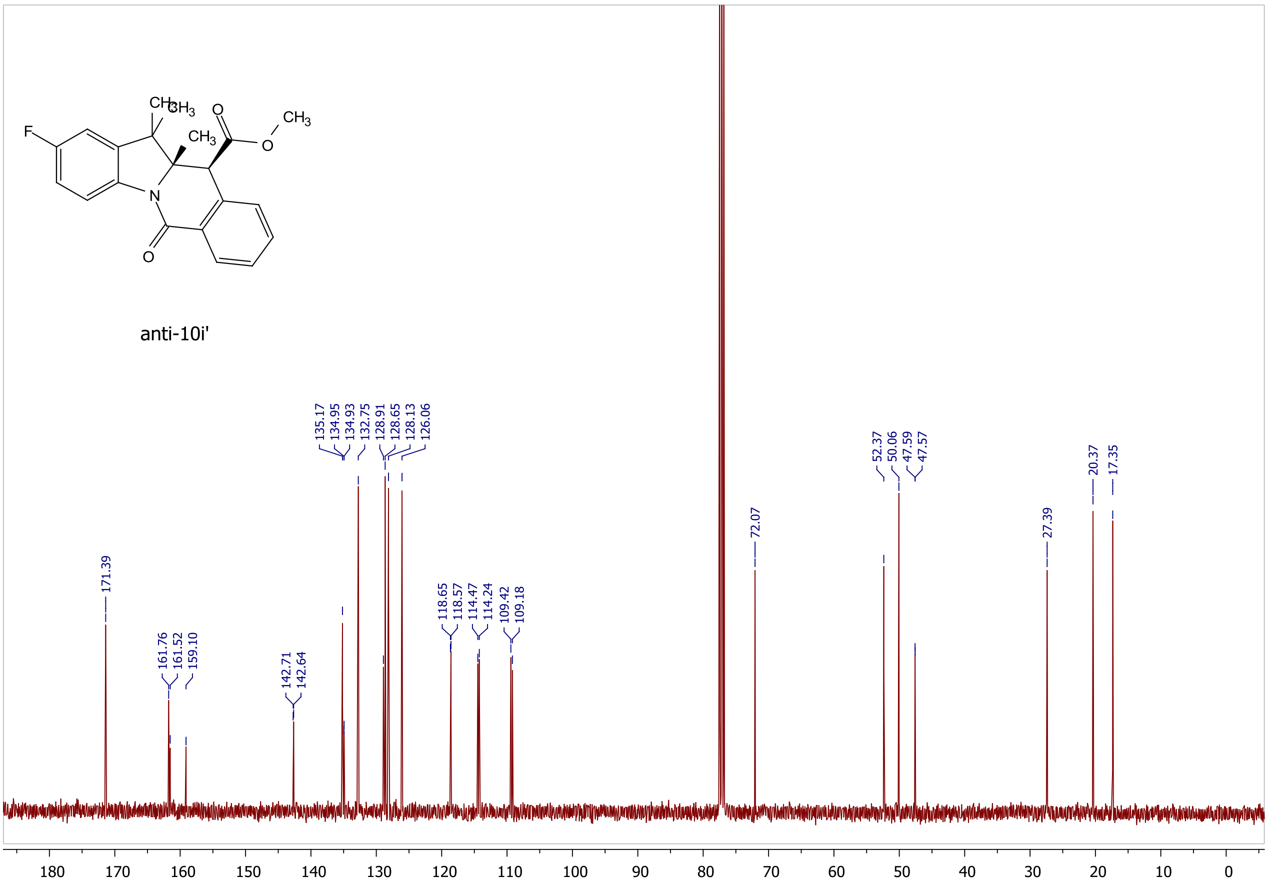
F
CH3 CH3
CH3
O
O
CH3
N
O
anti-10i'
171.39
161.76 161.52 159.10
142.71 142.64
135.17 134.95 134.93 132.75 128.91 128.65 128.13 126.06
118.65 118.57 114.47 114.24 109.42 109.18
72.07
52.37 50.06 47.59 47.57
27.39
20.37 17.35
180 170 160 150 140 130 120 110 100 90 80 70 60 50 40 30 20 10 0

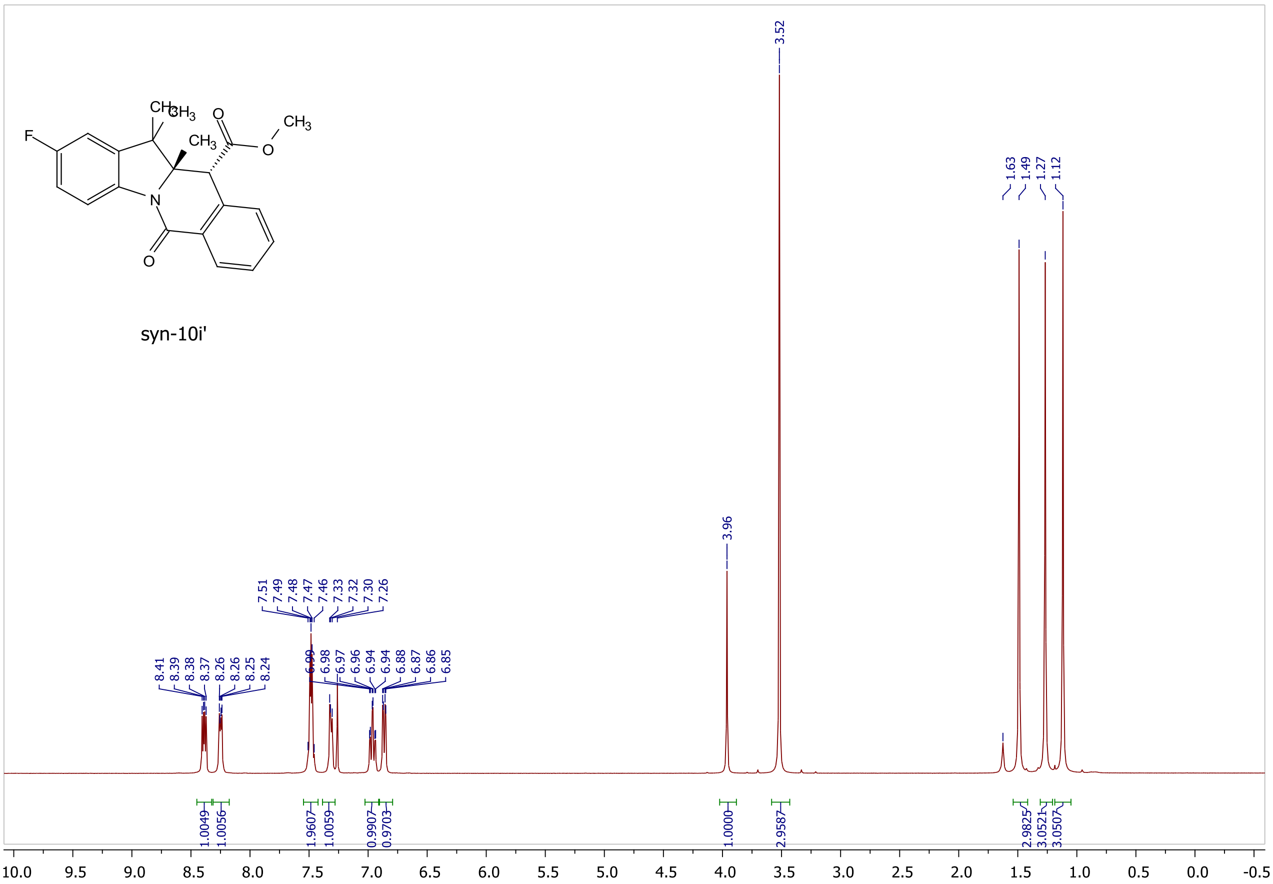

F
CH3
CH3
CH3
O
O
CH3
N
O
syn-10i'
8.41
8.39
8.38
8.37
8.26
8.26
8.25
8.24
7.51
7.49
7.48
7.47
7.46
7.33
7.32
7.30
7.26
6.99
6.98
6.97
6.96
6.94
6.94
6.88
6.87
6.86
6.85
3.96
3.52
1.63
1.49
1.27
1.12
1.0049
1.0056
1.9607
1.0059
0.9907
0.9703
1.0000
2.9587
2.9825
3.0521
3.0507
10.0 9.5 9.0 8.5 8.0 7.5 7.0 6.5 6.0 5.5 5.0 4.5 4.0 3.5 3.0 2.5 2.0 1.5 1.0 0.5 0.0 -0.5

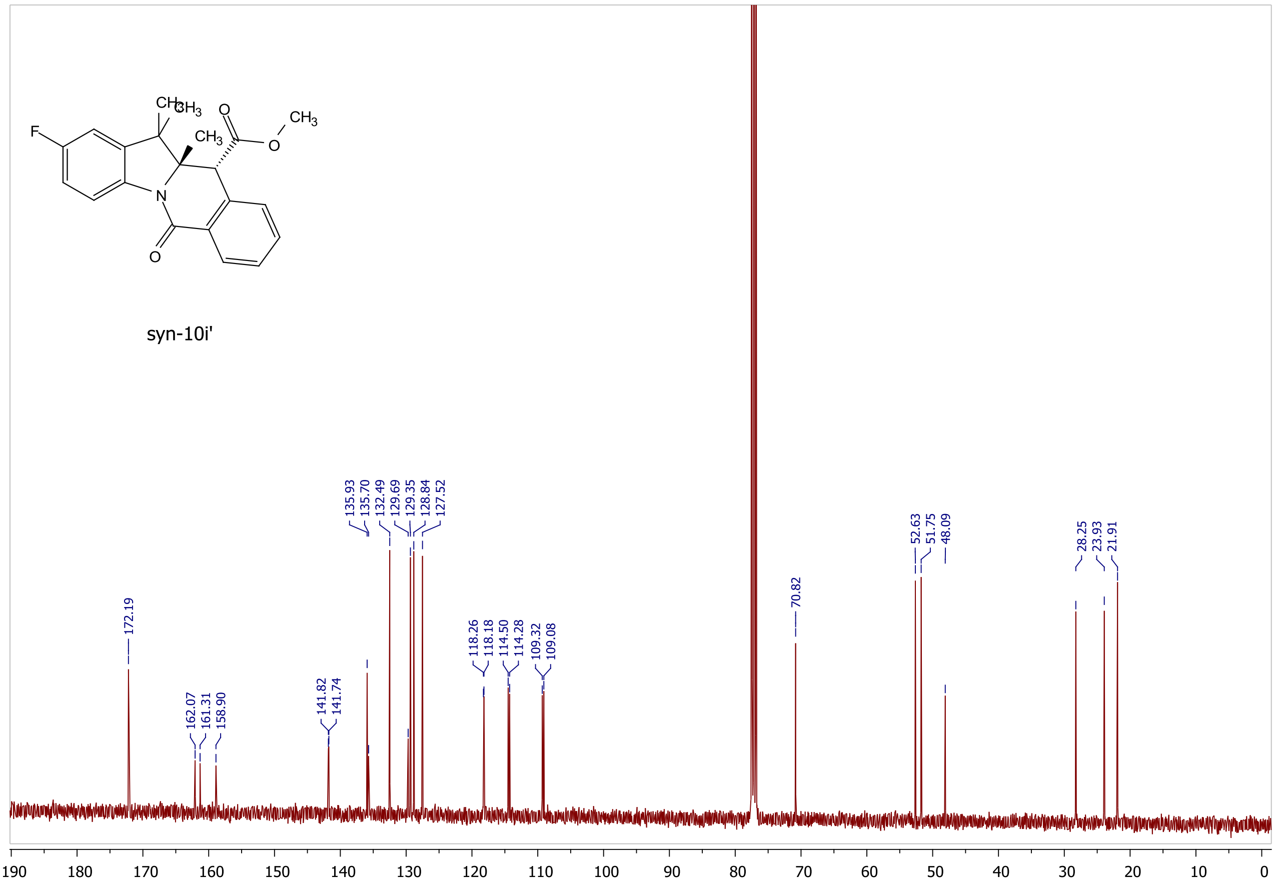

F
CH3
CH3
CH3
O
O
CH3
N
O
syn-10i'
172.19
162.07
161.31
158.90
141.82
141.74
135.93
135.70
132.49
129.69
129.35
128.84
127.52
118.26
118.18
114.50
114.28
109.32
109.08
70.82
52.63
51.75
48.09
28.25
23.93
21.91
190 180 170 160 150 140 130 120 110 100 90 80 70 60 50 40 30 20 10 0

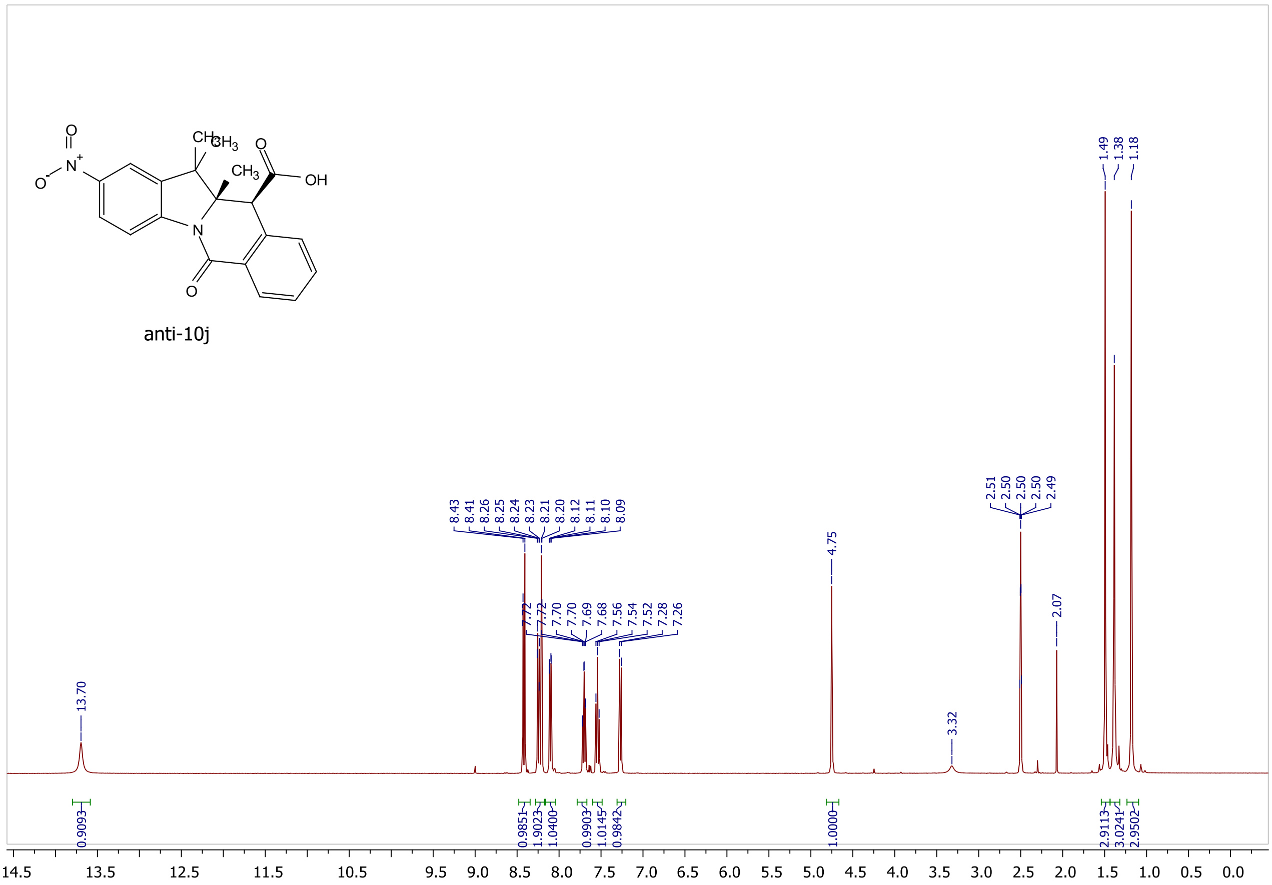
anti-10j
13.70
8.43
8.41
8.26
8.25
8.24
8.23
8.21
8.20
8.12
8.11
8.10
8.09
7.72
7.72
7.70
7.70
7.69
7.68
7.56
7.54
7.52
7.28
7.26
4.75
3.32
2.51
2.50
2.50
2.50
2.49
2.07
1.49
1.38
1.18
0.9093
0.9851
1.9023
1.0400
0.9903
1.0145
0.9842
1.0000
2.9113
3.0241
2.9502

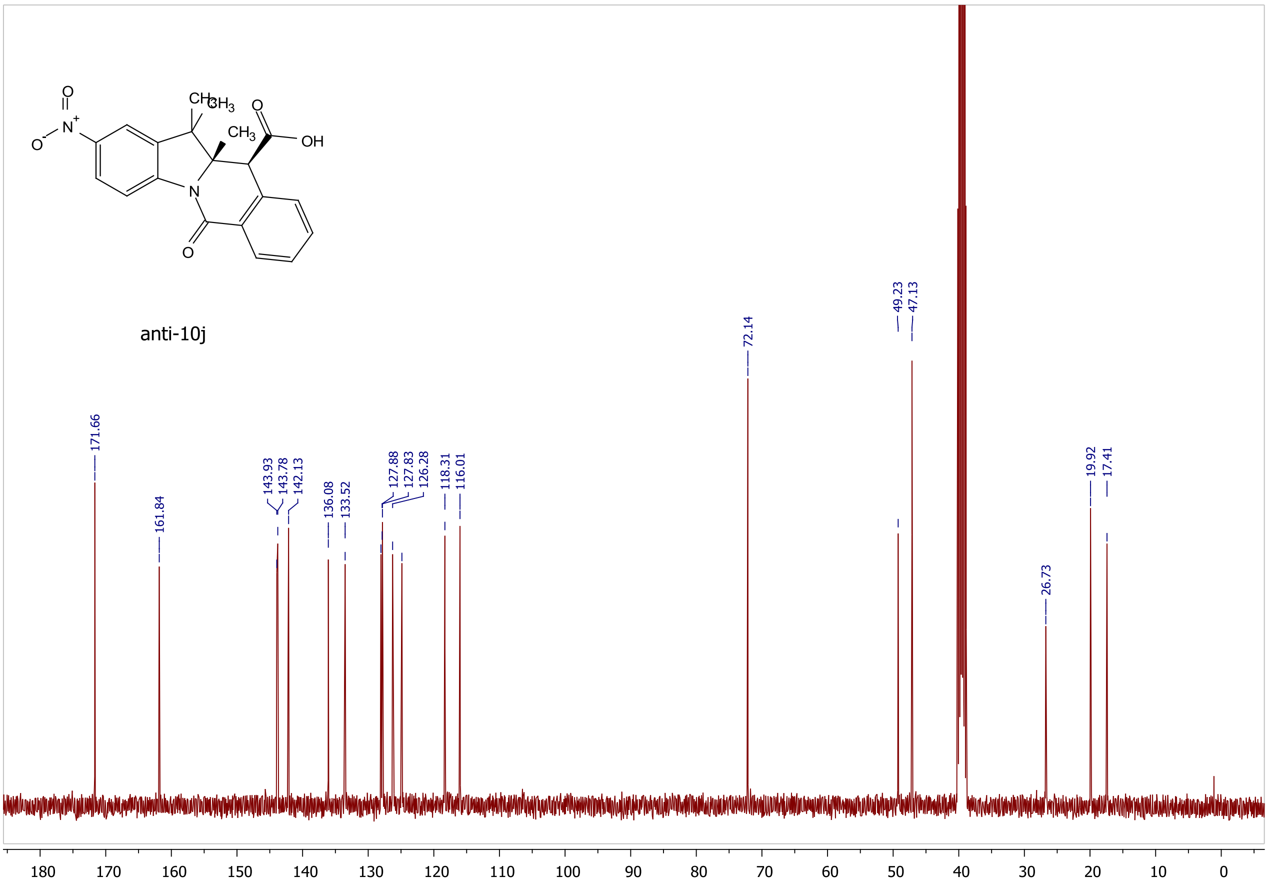
anti-10j
171.66
161.84
143.93
143.78
142.13
136.08
133.52
127.88
127.83
126.28
118.31
116.01
72.14
49.23
47.13
26.73
19.92
17.41

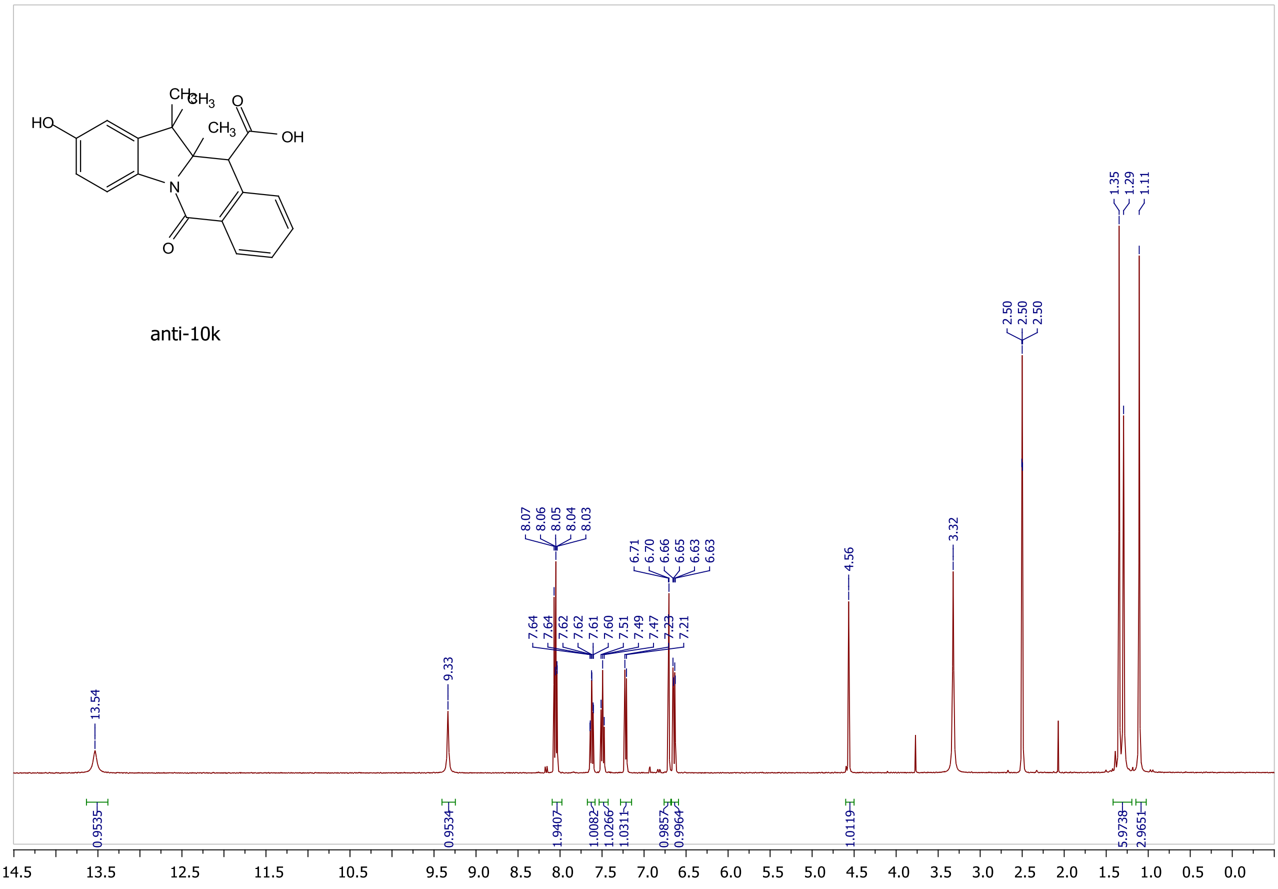
anti-10k

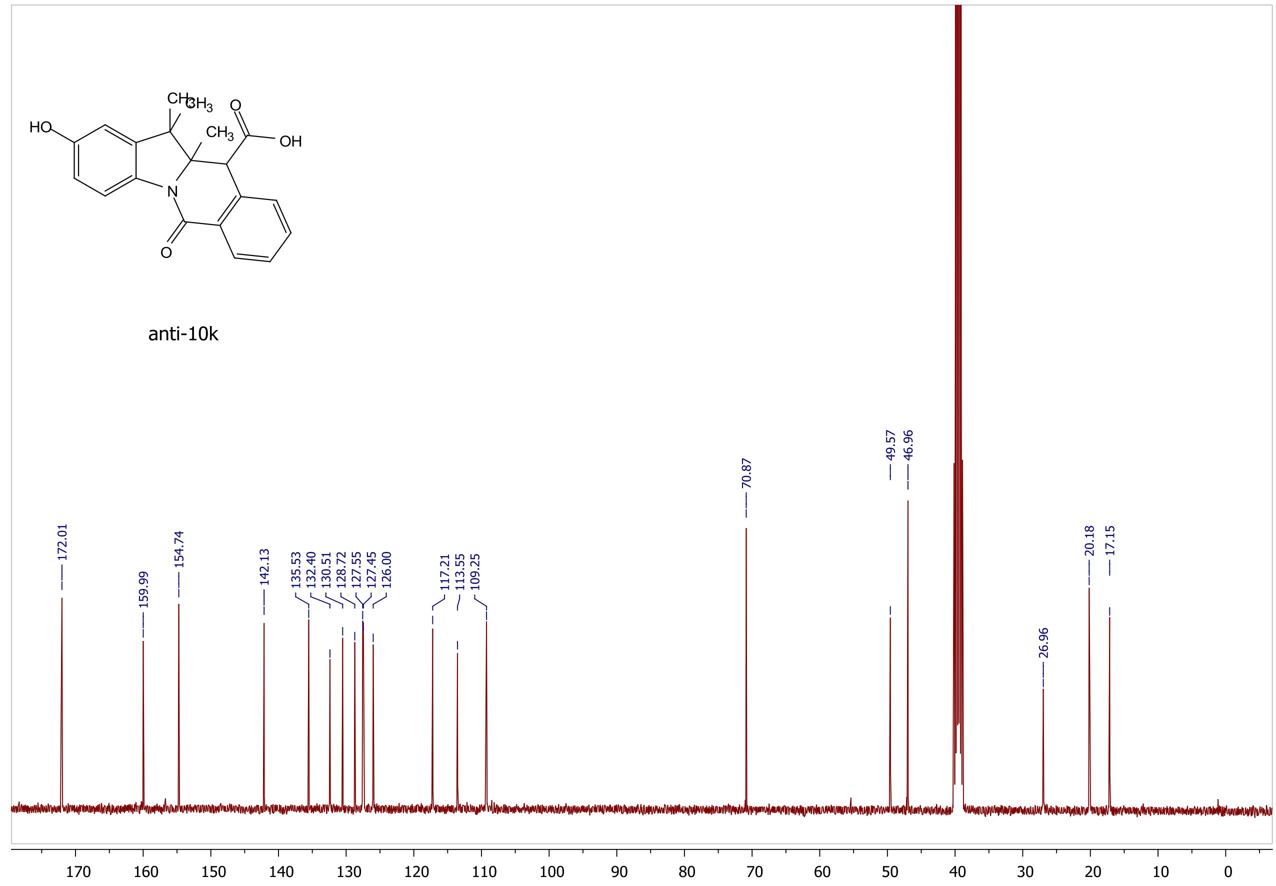
anti-10k

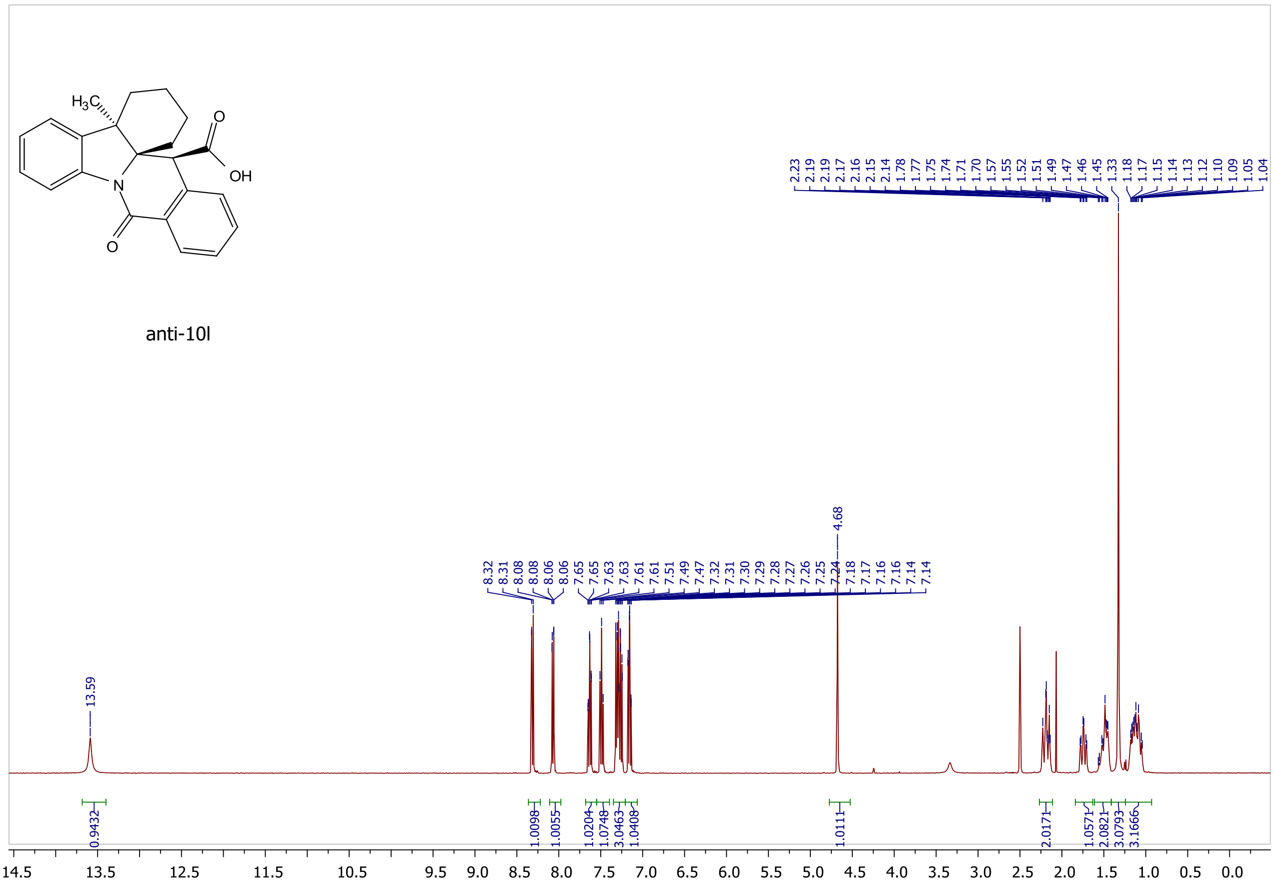
anti-10l
13.59
8.32
8.31
8.08
8.08
8.06
8.06
7.65
7.65
7.63
7.63
7.61
7.61
7.51
7.49
7.47
7.32
7.31
7.30
7.29
7.28
7.27
7.26
7.25
7.24
7.18
7.17
7.16
7.16
7.14
7.14
4.68
2.23
2.19
2.19
2.17
2.16
2.15
2.14
1.78
1.77
1.75
1.74
1.71
1.70
1.57
1.55
1.52
1.51
1.49
1.47
1.46
1.45
1.33
1.18
1.17
1.15
1.14
1.13
1.12
1.10
1.09
1.05
1.04
0.9432
1.0098
1.0055
1.0204
1.0748
3.0463
1.0408
1.0111
2.0171
1.0571
2.0821
3.0793
3.1666

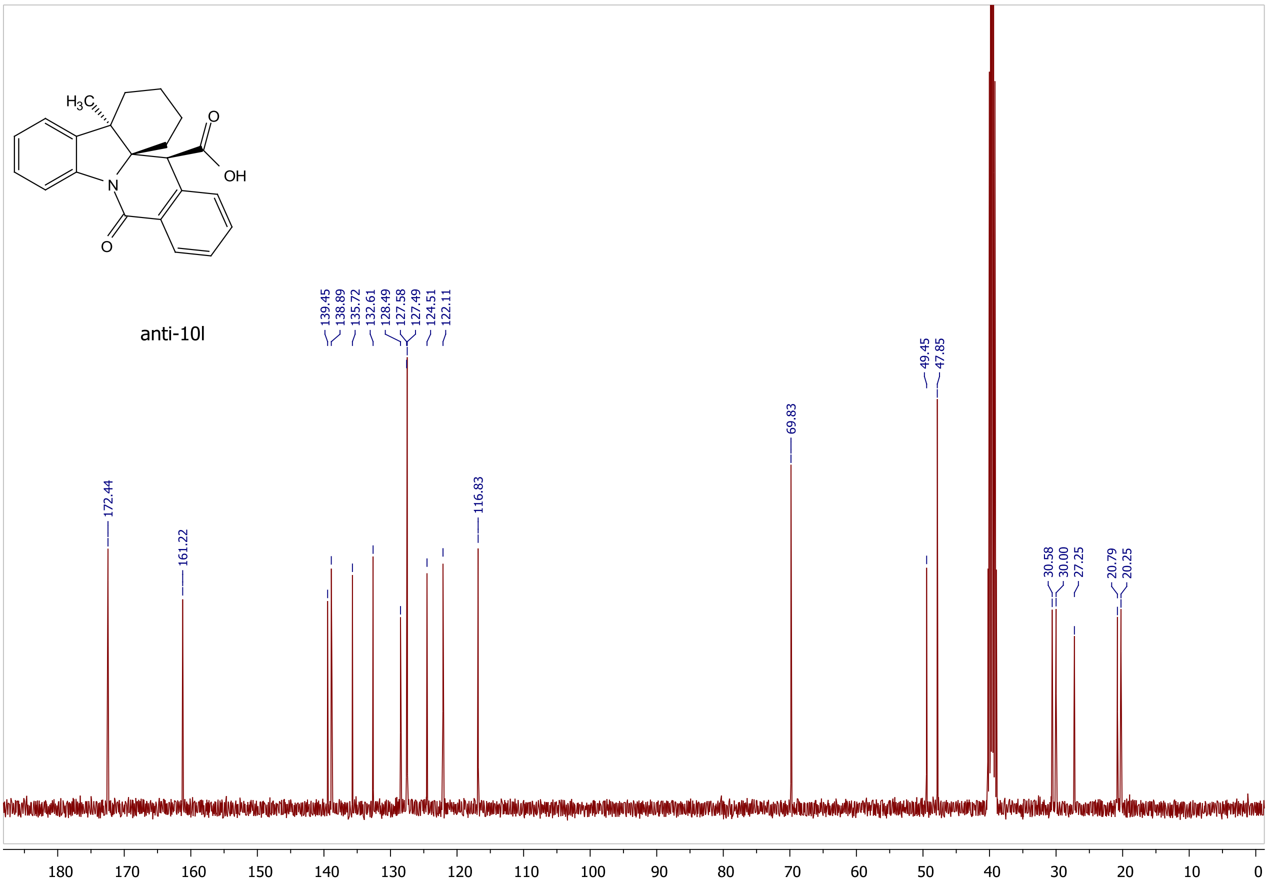
anti-10l
172.44
161.22
139.45
138.89
135.72
132.61
128.49
127.58
127.49
124.51
122.11
116.83
69.83
49.45
47.85
30.58
30.00
27.25
20.79
20.25

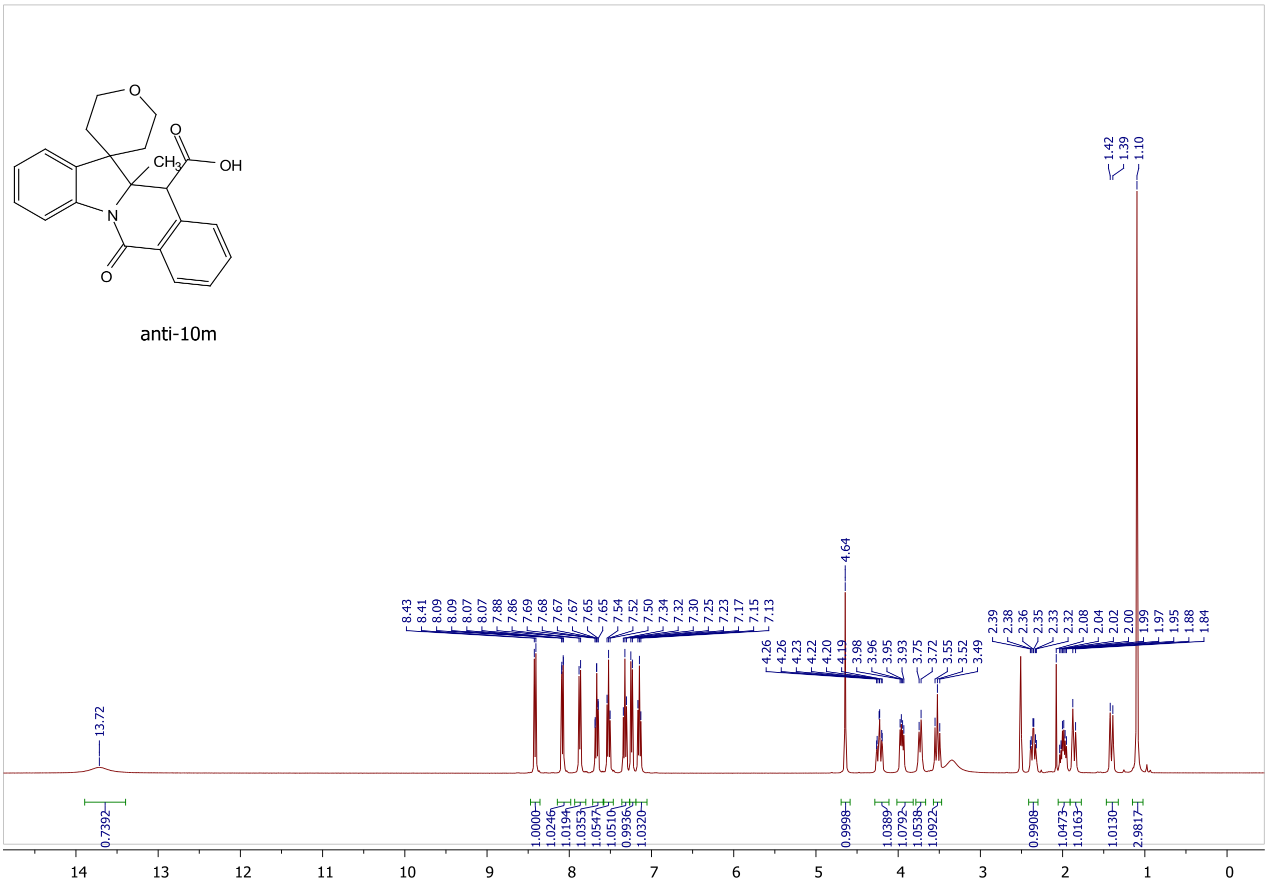

anti-10m
O
CH3
OH
N
O
13.72
8.43 8.41 8.09 8.09 8.07 8.07 7.88 7.86 7.69 7.68 7.67 7.65 7.65 7.54 7.52 7.50 7.34 7.32 7.30 7.25 7.23 7.17 7.15 7.13
4.64
4.26 4.26 4.23 4.22 4.20 4.19 3.98 3.96 3.95 3.93 3.75 3.72 3.55 3.52 3.49
2.39 2.38 2.36 2.35 2.33 2.32 2.08 2.04 2.02 2.00 1.99 1.97 1.95 1.88 1.84
1.42 1.39 1.10
0.7392 1.0000 1.0246 1.0194 1.0353 1.0547 1.0510 0.9936 1.0320 0.9998 1.0389 1.0792 1.0538 1.0922 0.9908 1.0473 1.0163 1.0130 2.9817
14 13 12 11 10 9 8 7 6 5 4 3 2 1 0

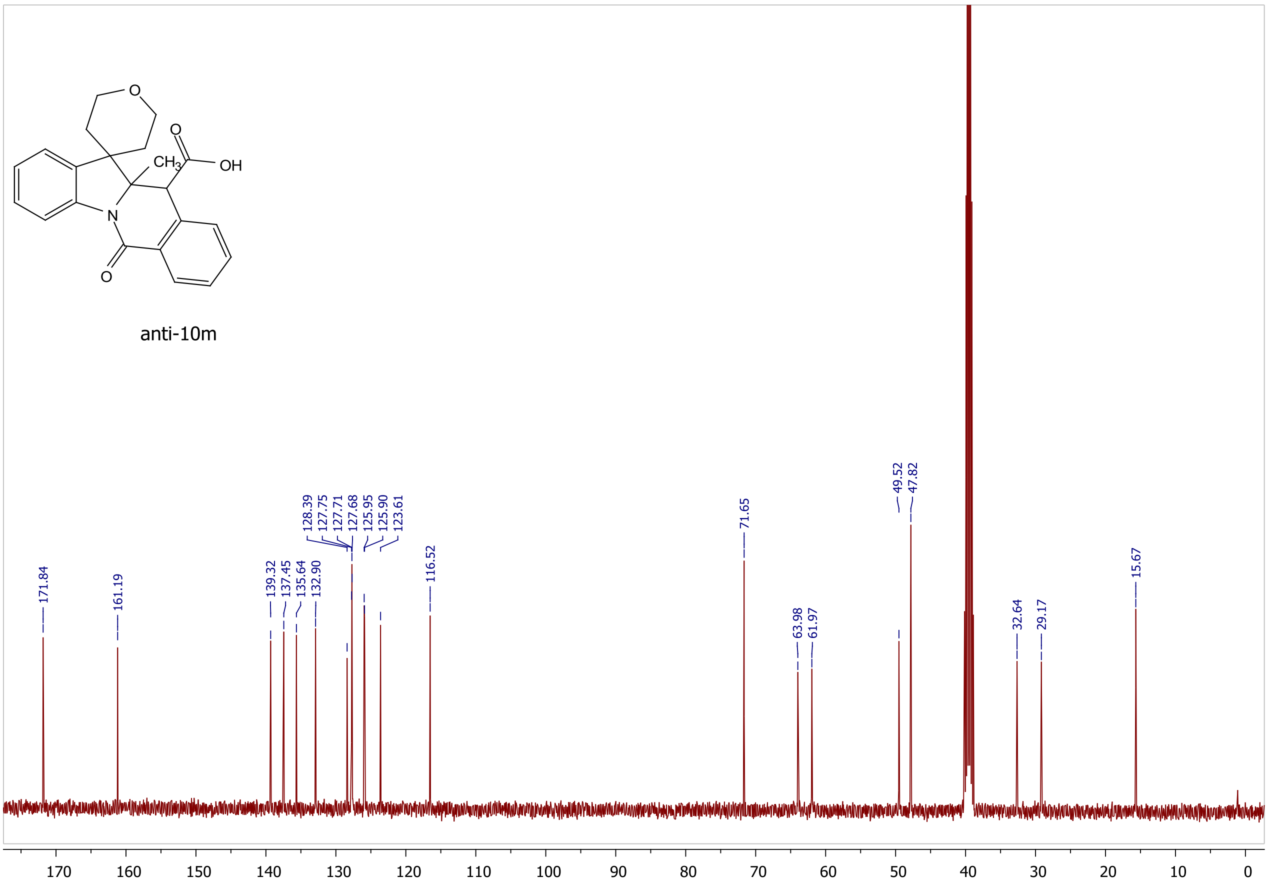

anti-10m
O
CH3
OH
N
O
171.84 161.19
139.32 137.45 135.64 132.90
128.39 127.75 127.71 127.68 125.95 125.90 123.61
116.52
71.65
63.98 61.97
49.52 47.82
32.64 29.17
15.67
170 160 150 140 130 120 110 100 90 80 70 60 50 40 30 20 10 0

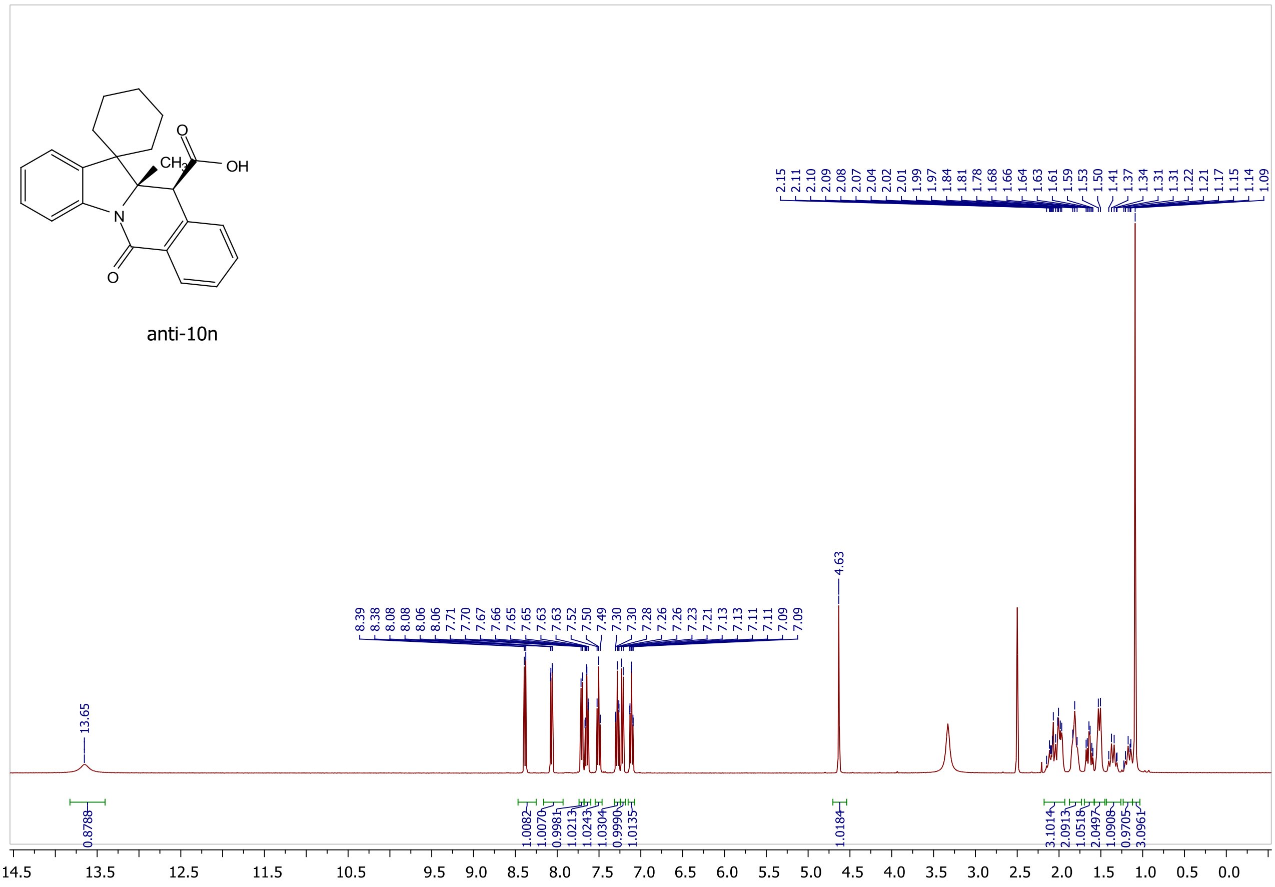
CH3
OH
O
N
O
anti-10n
13.65
8.39 8.38 8.08 8.08 8.06 8.06 7.71 7.70 7.67 7.66 7.65 7.65 7.63 7.63 7.52 7.50 7.49 7.30 7.30 7.28 7.26 7.26 7.23 7.21 7.13 7.13 7.11 7.11 7.09 7.09
4.63
2.15 2.11 2.10 2.09 2.08 2.07 2.04 2.02 2.01 1.99 1.97 1.84 1.81 1.78 1.68 1.66 1.64 1.63 1.61 1.59 1.53 1.50 1.41 1.37 1.34 1.31 1.22 1.21 1.17 1.15 1.14 1.09
0.8788
1.0082 1.0070 0.9981 1.0213 1.0243 1.0304 0.9990 1.0135
1.0184
3.1014 2.0913 1.0518 2.0497 1.0908 0.9705 3.0961
14.5 13.5 12.5 11.5 10.5 9.5 9.0 8.5 8.0 7.5 7.0 6.5 6.0 5.5 5.0 4.5 4.0 3.5 3.0 2.5 2.0 1.5 1.0 0.5 0.0

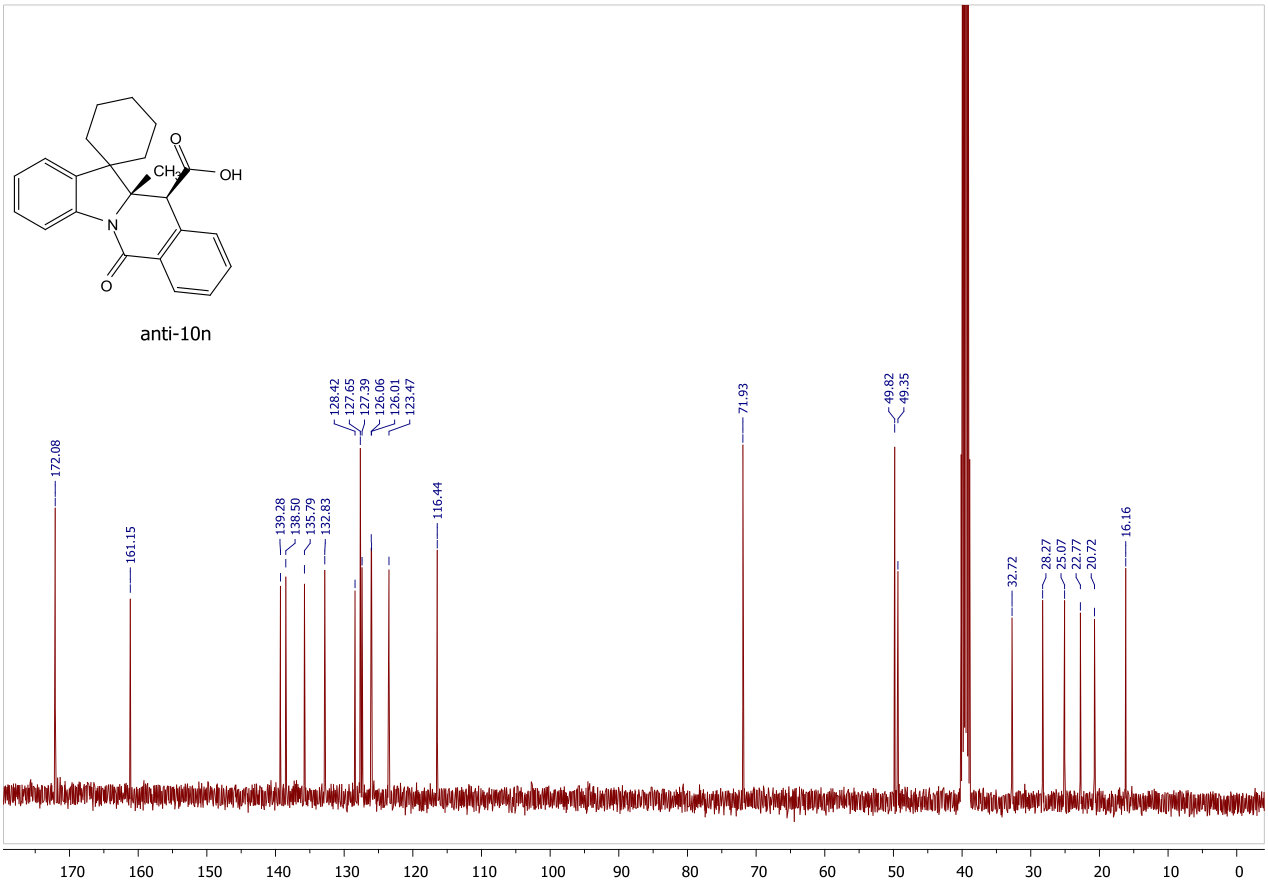
CH3
OH
O
N
O
anti-10n
172.08
161.15
139.28 138.50 135.79 132.83
128.42 127.65 127.39 126.06 126.01 123.47
116.44
71.93
49.82 49.35
32.72
28.27 25.07 22.77 20.72
16.16
170 160 150 140 130 120 110 100 90 80 70 60 50 40 30 20 10 0

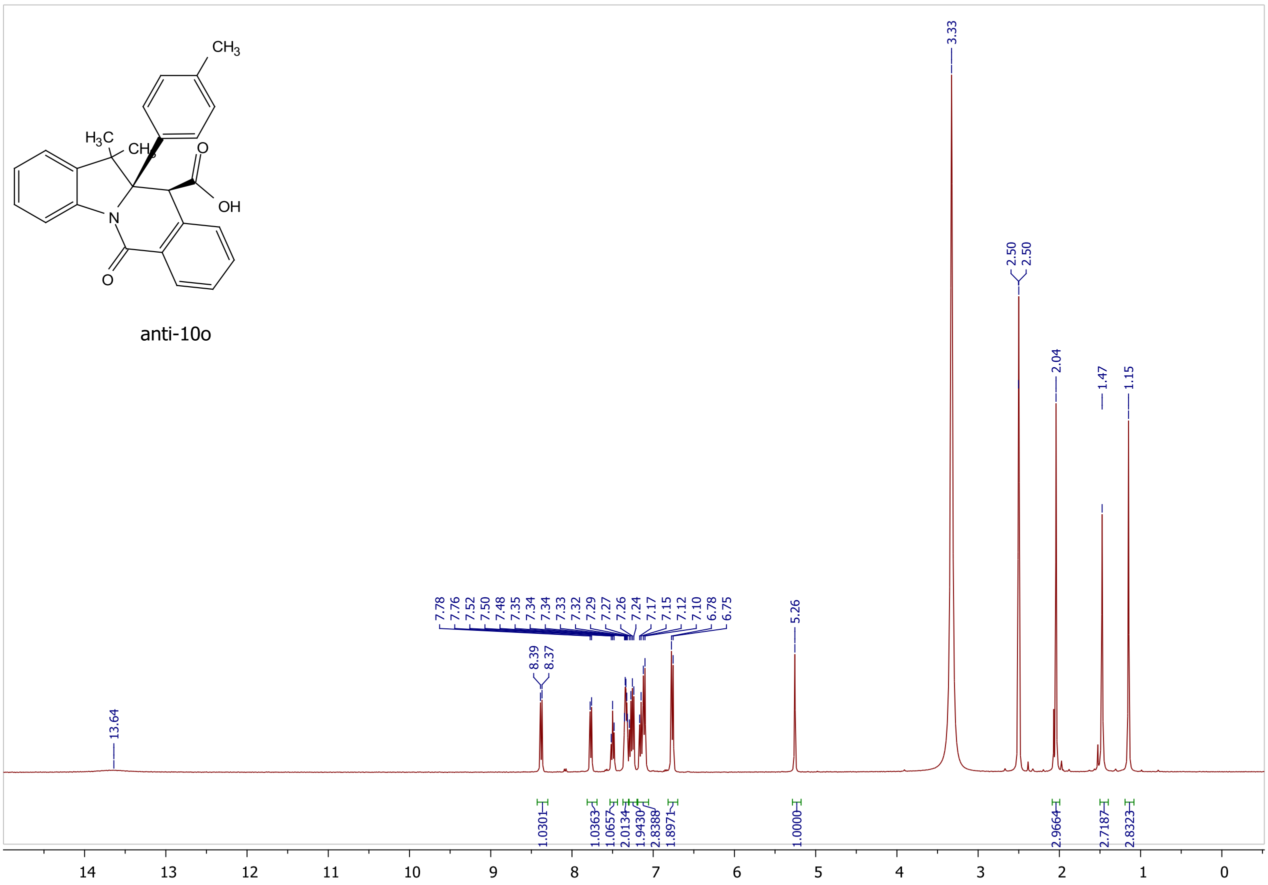
CH3
H3C
CH3
O
OH
N
O
anti-10o
13.64
8.39
8.37
7.78
7.76
7.52
7.50
7.48
7.35
7.34
7.34
7.33
7.32
7.29
7.27
7.26
7.24
7.17
7.15
7.12
7.10
6.78
6.75
5.26
3.33
2.50
2.50
2.04
1.47
1.15
1.0301
1.0363
1.0657
2.0134
1.9430
2.8388
1.8971
1.0000
2.9664
2.7187
2.8323
14
13
12
11
10
9
8
7
6
5
4
3
2
1
0

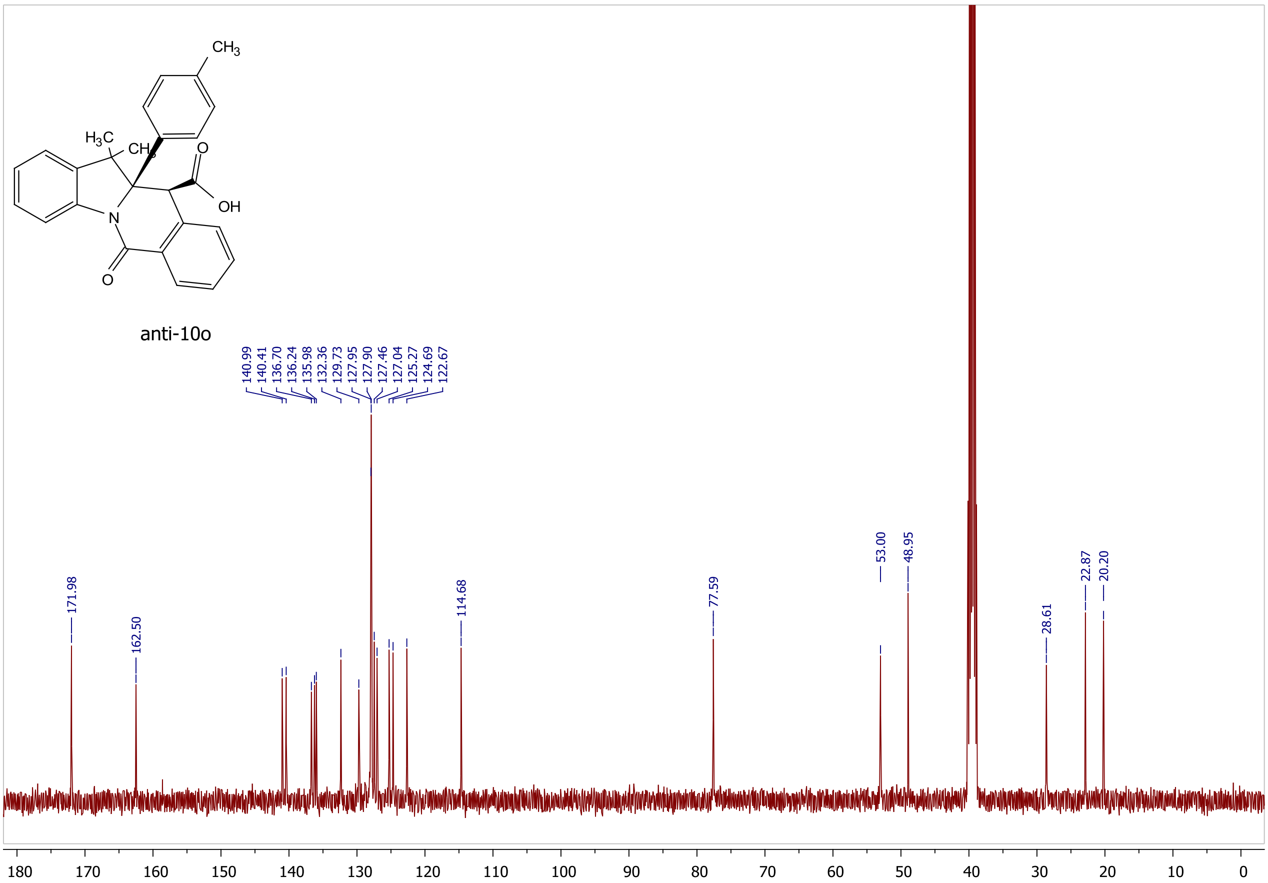
CH3
H3C
CH3
O
OH
N
O
anti-10o
140.99
140.41
136.70
136.24
135.98
132.36
129.73
127.95
127.90
127.46
127.04
125.27
124.69
122.67
171.98
162.50
114.68
77.59
53.00
48.95
28.61
22.87
20.20
180
170
160
150
140
130
120
110
100
90
80
70
60
50
40
30
20
10
0

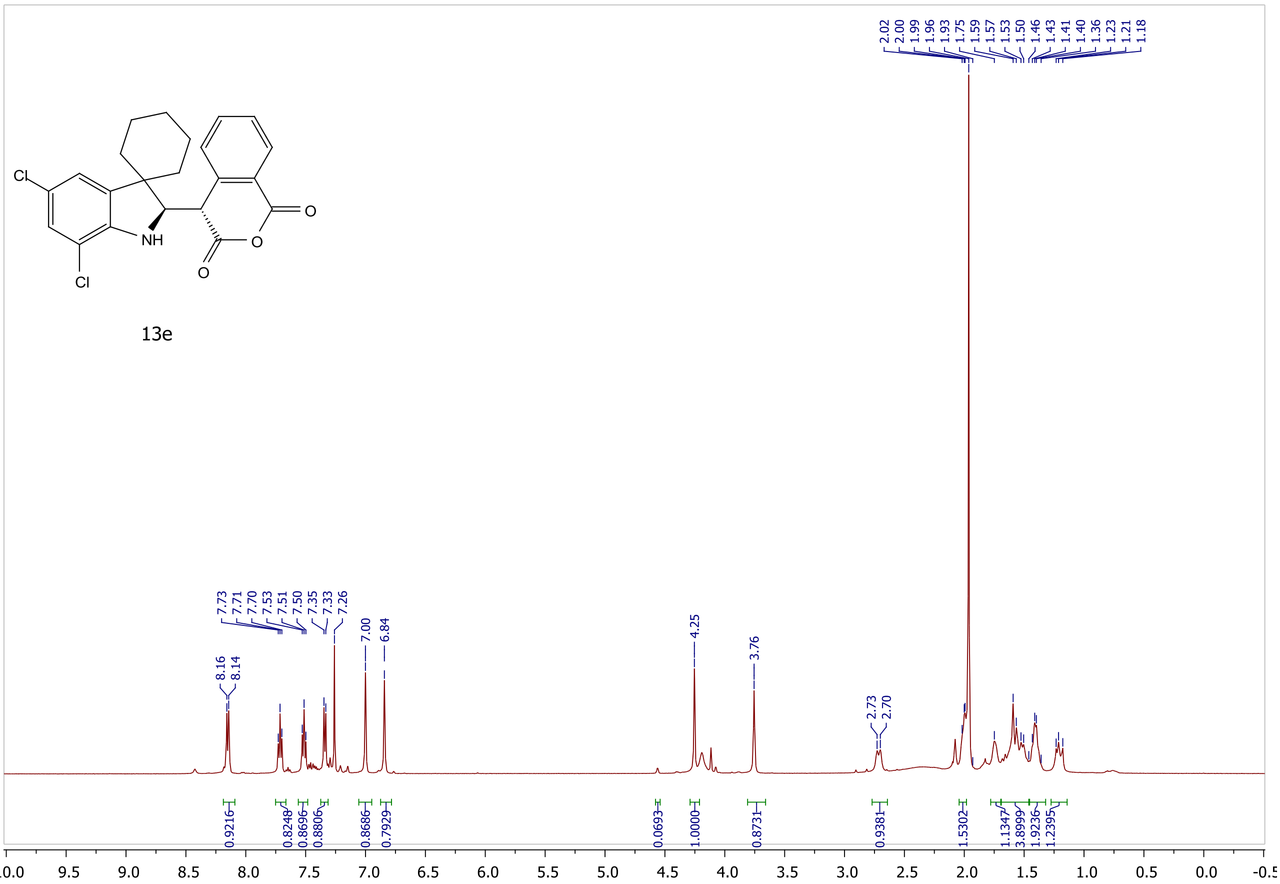
Cl
Cl
NH
O
O
O
13e
8.16
8.14
7.73
7.71
7.70
7.53
7.51
7.50
7.35
7.33
7.26
7.00
6.84
4.25
3.76
2.73
2.70
2.02
2.00
1.99
1.96
1.93
1.75
1.59
1.57
1.53
1.50
1.46
1.43
1.41
1.40
1.36
1.23
1.21
1.18
0.9216
0.8248
0.8696
0.8806
0.8686
0.7929
0.0693
1.0000
0.8731
0.9381
1.5302
1.1347
3.8999
1.9236
1.2395
10.0 9.5 9.0 8.5 8.0 7.5 7.0 6.5 6.0 5.5 5.0 4.5 4.0 3.5 3.0 2.5 2.0 1.5 1.0 0.5 0.0

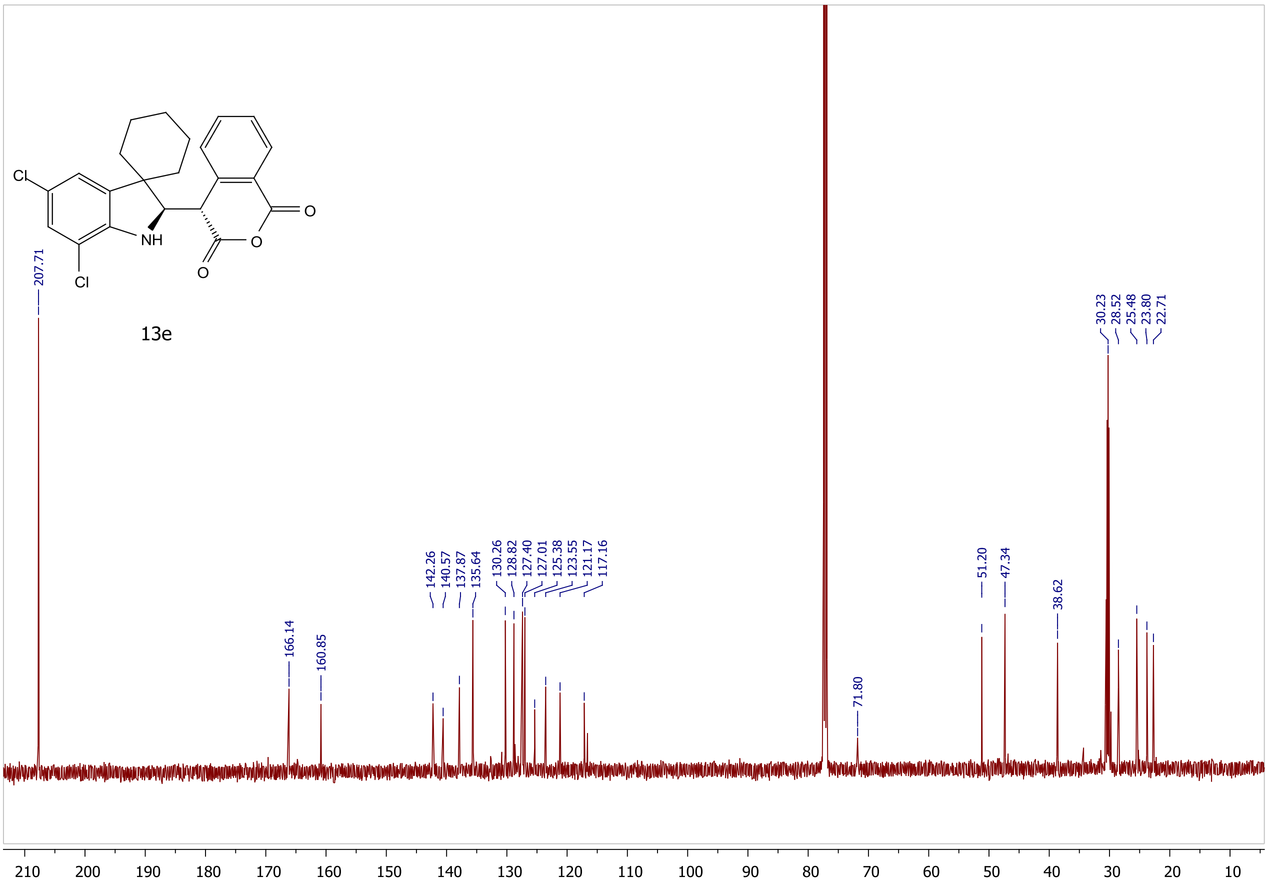
Cl
Cl
NH
O
O
O
13e
207.71
166.14
160.85
142.26
140.57
137.87
135.64
130.26
128.82
127.40
127.01
125.38
123.55
121.17
117.16
71.80
51.20
47.34
38.62
30.23
28.52
25.48
23.80
22.71
210 200 190 180 170 160 150 140 130 120 110 100 90 80 70 60 50 40 30 20 10

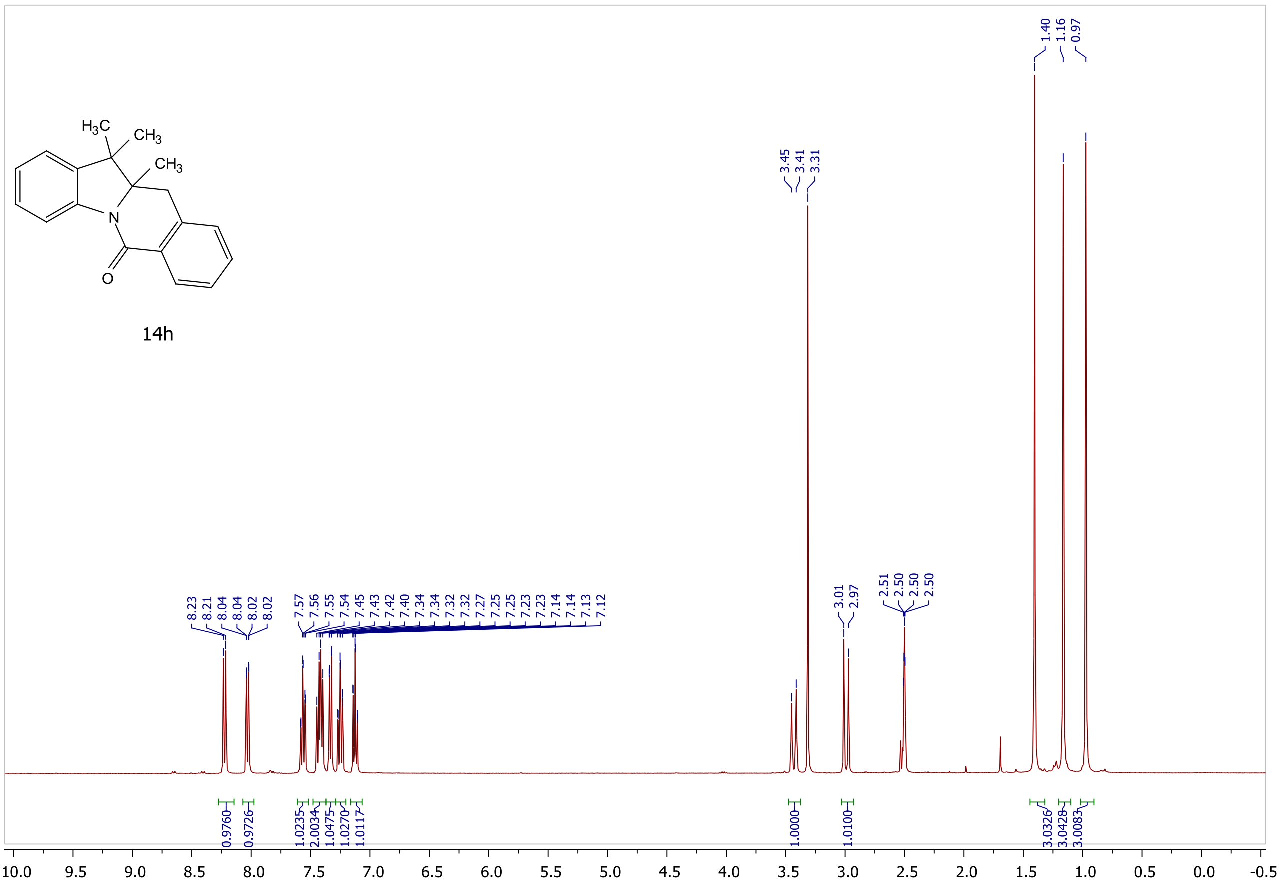
14h
8.23 8.21 8.04 8.04 8.02 8.02
7.57 7.56 7.55 7.54 7.45 7.43 7.42 7.40 7.34 7.34 7.32 7.32 7.27 7.25 7.25 7.23 7.23 7.14 7.14 7.13 7.12
3.45 3.41 3.31
3.01 2.97
2.51 2.50 2.50 2.50
1.40 1.16 0.97
0.9760 0.9726 1.0235 2.0034 1.0475 1.0270 1.0117 1.0000 1.0100 3.0326 3.0428 3.0083
10.0 9.5 9.0 8.5 8.0 7.5 7.0 6.5 6.0 5.5 5.0 4.5 4.0 3.5 3.0 2.5 2.0 1.5 1.0 0.5 0.0 -0.5

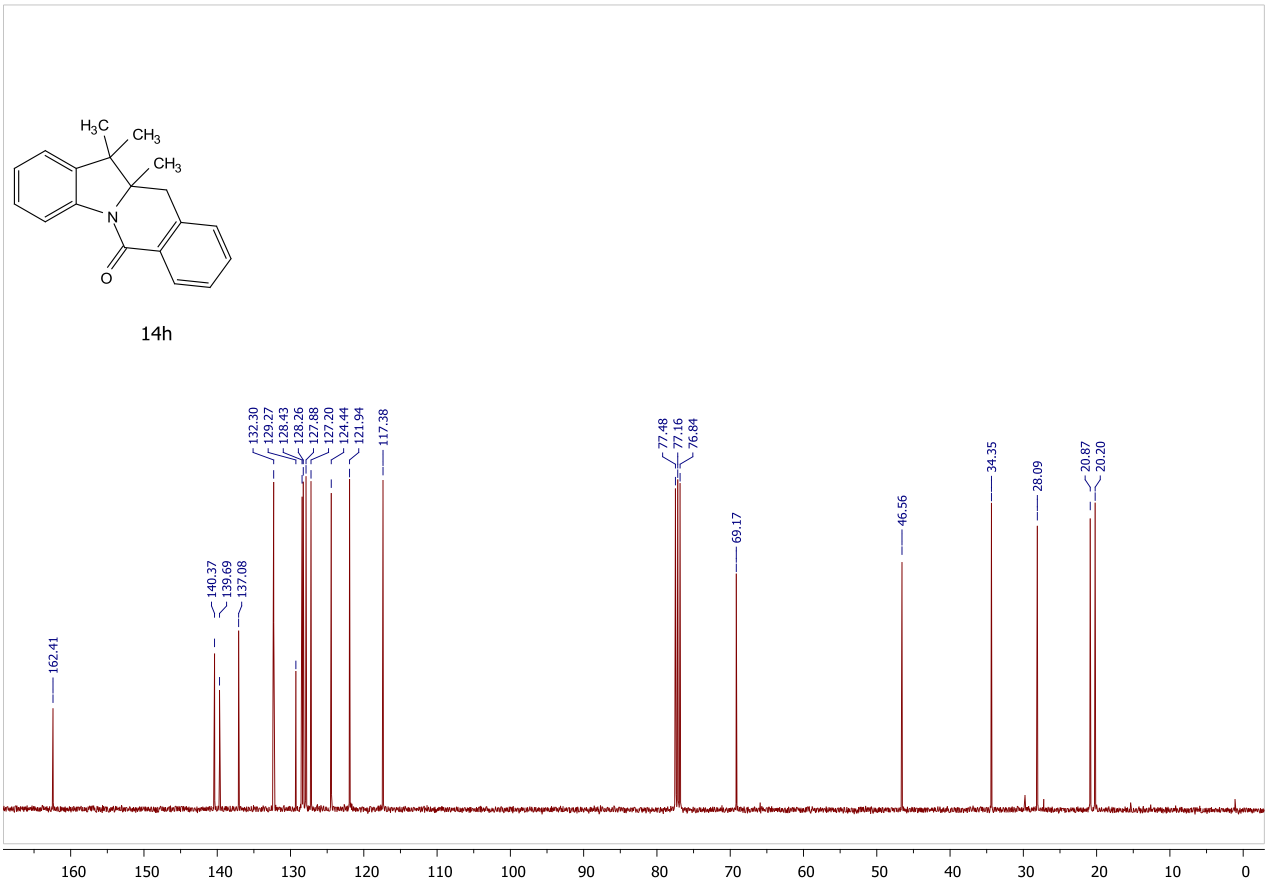
14h
162.41
140.37 139.69 137.08
132.30 129.27 128.43 128.26 127.88 127.20 124.44 121.94 117.38
77.48 77.16 76.84
69.17
46.56
34.35
28.09
20.87 20.20
160 150 140 130 120 110 100 90 80 70 60 50 40 30 20 10 0
